# A flexible count data model to fit the wide diversity of expression profiles arising from extensively replicated RNA-seq experiments

**DOI:** 10.1186/1471-2105-14-254

**Published:** 2013-08-21

**Authors:** Mikel Esnaola, Pedro Puig, David Gonzalez, Robert Castelo, Juan R Gonzalez

**Affiliations:** 1 Center for Research in Environmental Epidemiology (CREAL), Barcelona, Spain; 2 Department of Mathematics, Universitat Autònoma de Barcelona (UAB), Barcelona, Spain; 3 Center for Genomic Regulation (CRG), Barcelona, Spain; 4 Department of Experimental and Health Sciences, Research Program on Biomedical Informatics (GRIB), Universitat Pompeu Fabra, Barcelona, Spain; 5 Hospital del Mar Research Institute (IMIM), Barcelona, Spain; 6 CIBER Epidemiology and Public Health (CIBERESP), Barcelona, Spain

## Abstract

**Background:**

High-throughput RNA sequencing (RNA-seq) offers unprecedented power to capture the real dynamics of gene expression. Experimental designs with extensive biological replication present a unique opportunity to exploit this feature and distinguish expression profiles with higher resolution. RNA-seq data analysis methods so far have been mostly applied to data sets with few replicates and their default settings try to provide the best performance under this constraint. These methods are based on two well-known count data distributions: the Poisson and the negative binomial. The way to properly calibrate them with large RNA-seq data sets is not trivial for the non-expert bioinformatics user.

**Results:**

Here we show that expression profiles produced by extensively-replicated RNA-seq experiments lead to a rich diversity of count data distributions beyond the Poisson and the negative binomial, such as Poisson-Inverse Gaussian or Pólya-Aeppli, which can be captured by a more general family of count data distributions called the Poisson-Tweedie. The flexibility of the Poisson-Tweedie family enables a direct fitting of emerging features of large expression profiles, such as heavy-tails or zero-inflation, without the need to alter a single configuration parameter. We provide a software package for R called tweeDEseq implementing a new test for differential expression based on the Poisson-Tweedie family. Using simulations on synthetic and real RNA-seq data we show that tweeDEseq yields *P*-values that are equally or more accurate than competing methods under different configuration parameters. By surveying the tiny fraction of sex-specific gene expression changes in human lymphoblastoid cell lines, we also show that tweeDEseq accurately detects differentially expressed genes in a real large RNA-seq data set with improved performance and reproducibility over the previously compared methodologies. Finally, we compared the results with those obtained from microarrays in order to check for reproducibility.

**Conclusions:**

RNA-seq data with many replicates leads to a handful of count data distributions which can be accurately estimated with the statistical model illustrated in this paper. This method provides a better fit to the underlying biological variability; this may be critical when comparing groups of RNA-seq samples with markedly different count data distributions. The tweeDEseq package forms part of the Bioconductor project and it is available for download at http://www.bioconductor.org.

## Background

High-throughput gene expression profiling across samples constitutes one of the primary tools for characterizing phenotypes at molecular level. One of the main advantages of the rapidly evolving massive scale cDNA sequencing assay for this purpose (RNA-seq [1]), over the hybridization-based microarray technology, is a much larger dynamic range of detection. However, the extent to which this feature is fully exploited depends entirely on the way the resulting data is analyzed when addressing a particular biological question. For instance, in the identification of genes that significantly change their expression levels between groups of samples, also known as differential expression (DE).

For DE analysis, after some pre-processing steps that include the alignment of the sequenced reads to a reference genome and their summarization into features of interest (e.g., genes), raw RNA-seq data is transformed into an initial table of counts. This table should then be normalized [2-4] in order to adjust for both technical variability and the expression properties of the samples, such that the estimated normalization factors and offsets applied to the RNA-seq count data describe as accurately as possible the relative number of copies of each feature throughout every sample. As opposed to the continuous nature of log-scale fluorescence units in microarray data, RNA-seq expression levels are defined by discrete count data, and therefore, require specific DE analysis techniques.

**Figure 1:**
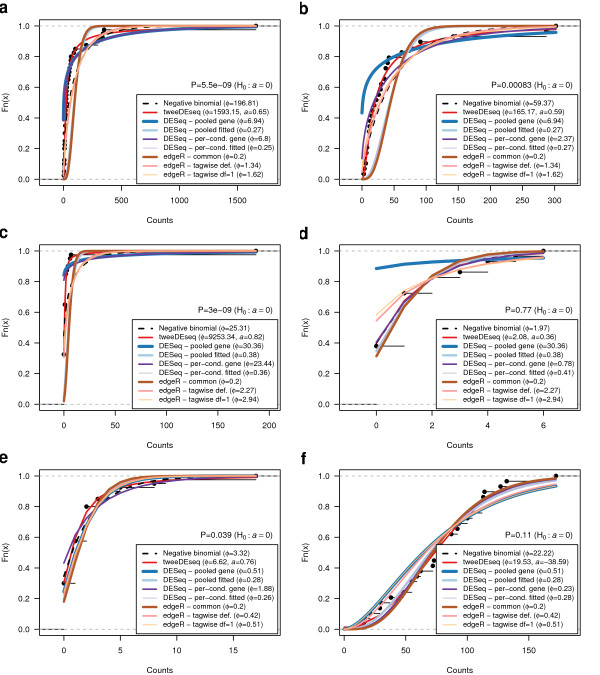
**Fit of different count data distributions to diverse RNA-seq gene expression profiles.** Fit of different count data distributions to the female **(a, c, e)** and male **(b, d, f)** RNA-seq expression profiles of genes *EEF1A2***(a, b)**, *SCT***(c, d)** and *NLGN4Y***(e, f)**. All plots show the empirical cumulative distribution function (CDF) of counts (black dots) and the CDF estimated by a pure negative binomial model (black dashed line), a Poisson-Tweedie model (red line) obtained with tweeDEseq and several moderated negative binomial models obtained with different parameter configurations of DESeq and edgeR. Estimated dispersions, and shape in the case of tweeDEseq, are indicated in the legend. Above the legend, the *P*-value of the test of goodness-of-fit to a negative binomial distribution is shown. According to this test, expression profiles in panels **(a, b, c** and **e)** do not follow a negative binomial distribution. Female samples display non-negative binomial features such as a heavy-tail **(a, c)** and zero-inflation **(c, e)**. Gene *NLGN4Y* is documented in the literature as a gene with sex-specific expression, while the other two are not (*EEF1A2* is a housekeeping gene and *SCT* is an endocrine hormone peptide in chromosome 11 that controls secretions in the duodenum).

Detection of DE genes using RNA-seq data was firstly based on using models assuming a Poisson distribution [5] with one single parameter, the mean, which simultaneously determines the variance of the distribution. Given that the observed variation in read counts is much larger than the mean (overdispersion), researchers have proposed the use of negative binomial (NB) distributions [6-8] which are defined by two parameters: the mean and the dispersion. However, the larger power of RNA-seq to capture biological variability can potentially introduce into count data not only overdispersion, but also oddities such as zero-inflation (i.e., in lowly expressed genes, the proportion of zero counts may be greater than expected under an NB distribution) and heavy tail behavior (i.e., a large dynamic range within the same expression profile), specially when many biological replicates are available. Under these circumstances even a two-parameter NB distribution may not provide an adequate fit to the data (see Figure 1). In turn, this may lead to incorrect statistical inferences resulting in lists of DE genes with a potentially increased number of false positive calls and poor reproducibility. To overcome this problem, methods based on the NB distribution [6-11] use sophisticated moderation techniques that borrow information across genes and exploit the mean-variance relationship in count data to improve the estimation of the NB dispersion parameter. This requires, however, that the parameter configuration is calibrated for the most appropriate moderation regime which may depend on features such as sample size, the magnitude of the fold-change, the variability of expression levels, the fraction of genes undergoing differential expression and the overall expression level.

In this paper we propose to approach this problem by using other count data distributions that fit expression profiles better than the NB without the need to alter configuration parameters. The rest of the paper is organized as follows. Using a large RNA-seq data set of HapMap lymphoblastoid cell lines (LCLs) derived from *n*=69 unrelated Nigerian (YRI) individuals [12], we start by assessing the goodness of fit of extensively replicated expression profiles to the NB distribution, showing a lack of fit for an important fraction of genes. We illustrate how a more flexible family of count-data probability distributions, called the Poisson-Tweedie, provides a better fit to these expression profiles. We provide data supporting the hypothesis that the lack of fit to NB distributions may be related to the dynamics of gene expression unveiled by RNA-seq technology. We then introduce a new test for differential expression analysis in RNA-seq data based on the Poisson-Tweedie family of distributions. We demonstrate with simulations on synthetic and real RNA-seq data how a single run of our approach provides *P*-values that are equally or more accurate than NB-based competing methods calibrated with a variety of configuration parameters. Finally, by surveying the tiny fraction of sex-specific gene expression changes in LCL samples, we approach the problem of assessing accuracy in DE analysis with real RNA-seq data and show that, in the context of extensively replicated RNA-seq experiments, tweeDEseq yields better performance than competing NB-based methods without the need to make an informed decision on the configuration of parameters. This improvement is reported in terms of precision and recall of DE genes and reproducibility of the significance levels with respect to matching microarray experiments.

## Results and discussion

**Figure 2:**
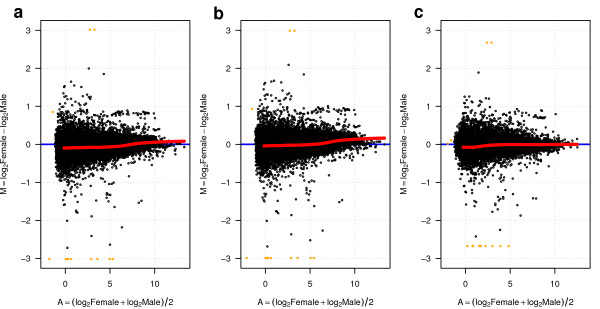
**Count data normalization.** MA-plots of the count data corresponding to the YRI samples from Pickrell [12]*et al.* (2010) after applying the following normalization methods: **(a)** raw count data without any normalization; **(b)** normalization with the edgeR[2] package; and **(c)** normalization with the cqn[4] package. The *x*-axis (A) shows the average expression throughout female and male samples in log2 scale and the *y*-axis (M) shows the magnitude of the log2-fold change between female and male samples.

The results we provide in this paper are based on data from a previously published large RNA-seq experiment [12] and on our own simulated count data. We downloaded and pre-processed the HapMap LCL raw RNA-seq data, consisting of *n*=69 samples from unrelated YRI individuals, with our own pipeline (see Methods). The resulting table of counts consists of 38,415 genes by 69 samples. We filtered out genes with very low expression levels and used different normalization methods [2,4] (see Methods) to ensure that the results described below do not depend on this fundamental step. In fact, we have observed that normalized counts can lead to quite different MA-plots depending on the normalization method, thus potentially affecting DE detection power and accuracy (Figure 2).

The statistical methods proposed in this paper are implemented in a package for the statistical software R, called tweeDEseq which forms part of the Bioconductor project [13] at http://www.bioconductor.org. We have also created an experimental data package, called tweeDEseqCountData, which contains the previously described data set and is also available at the same URL. All results presented in the paper were obtained using these and other packages from R version 2.15.1 and Bioconductor version 2.11, and can be reproduced through the scripts available as Additional file 1 to this article.

### Review of competing methods

There is currently a large body of literature on DE analysis methods for RNA-seq data [5-11,14], nearly all of them based on the NB distribution and developed to deliver their best performance with few replicates. Anders *et al.* (2010) [7] argued that for large number of individuals “... questions of data distribution could be avoided by using non-parametric methods, such as rank-based permutation tests”. However, rank-based methods require similar count data distributions between sample groups. Due to the large variability across groups [15] captured by RNA-seq data, this assumption will most likely be broken in this context. For example, panels e-f in Figure 1 illustrate the case of gene *NLGN4Y* (ENSG00000165246), a gene located in the male-specific region of chromosome Y and reported to have sex-specific expression, which shows remarkably different count data distributions between male and female samples. Permutation tests are also underpowered since distribution tails are not well estimated (due to the large dynamic range), which is important when correcting for multiple testing.

In this paper we will focus our comparisons on the two most widely used methods for DE analysis of RNA-seq data, edgeR[6,8,10] (version 3.0.8) and DESeq[7] (version 1.10.1) and explore those parameter configurations in these methods that we found most relevant for large RNA-seq data sets, according to the available documentation. Both, edgeR and DESEq, assume that expression profiles from RNA-seq data follow an NB distribution and borrow information across genes to first estimate a common dispersion parameter. Then, for each gene, they estimate its genewise dispersion and moderate it towards the common one. The way in which this moderation takes place depends on the method and its configuration parameters. DESeq[7] allows switching between common (sharingMode=~fit-only~) and genewise (sharingMode=~gene-est-only~) dispersions. It provides a straightforward strategy (sharingMode=~maximum~, default configuration) to choose between common and genewise dispersions by taking the largest value for each gene. edgeR allows one to calibrate, using the prior.df parameter, the transition from a purely genewise dispersion estimate (small values of prior.df) to the common one (large values of prior.df) by using an empirical Bayes approach. By default prior.df=20 which implies that a large weight is given to the common dispersion. However, according to the documentation, if the number of samples is large, the common dispersion becomes less important in the moderation step. Additional options in DESeq and edgeR that may be relevant in the context of large RNA-seq data sets are, in the case of DESeq, whether dispersions are estimated from the entire pool of samples (method=~pooled~, its default) or separately per sample group (method=~per-condition~). In the case of edgeR, whether the DE test is performed using a likelihood ratio test (glmLRT() function) or a quasi-likelihood F-test [8] (glmQLFTest() function), after dispersions are estimated. Table 1 summarizes these eight combinations of methods and parameter configurations and contains the key to the terms used in some figures to distinguish among them.

**Table 1:** Methods and parameter configurations compared in this paper

**Key**	**Software**	**Configuration parameters**
DESeqPgO	DESEq	method=~pooled~, sharingMode=~per-condition~
DESeqPmax	DESEq	method=~pooled~, sharingMode=~maximum~
DESeqCgO	DESEq	method=~per-condition~, sharingMode=~per-condition~
DESeqCmax	DESEq	method=~per-condition~, sharingMode=~maximum~
edgeRdf20	edgeR	common/trended/tagwise moderation regime with prior.df=20 (default)
edgeRdf1	edgeR	common/trended/tagwise moderation regime with prior.df=1
edgeRqlfDf20	edgeR	common/trended/tagwise moderation regime with prior.df=20 (default) and quasi-likelihood F-tests
edgeRqlfDf1	edgeR	common/trended/tagwise moderation regime with prior.df=1 and quasi-likelihood F-tests

### Different gene expression dynamics require different distributional assumptions on count data

**Figure 3:**
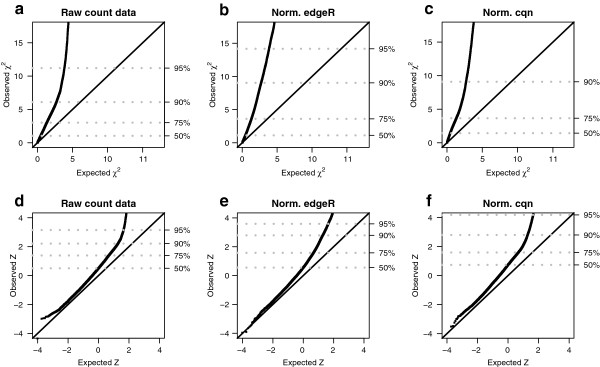
**Goodness of fit to the negative-binomial distribution.** Quantile-quantile (Q-Q) plots of the goodness-of-fit of RNA-seq expression profiles from Pickrell [12]*et al.* (2010) to a negative-binomial (NB) distribution. The right *y*-axis indicates the quantile of the observed distribution. Columns correspond to different normalization methods where **(a, d)** correspond to raw un-normalized counts, **(b, e)** normalization with edgeR and **(c, f)** normalization with cqn. The top row **(a, b, c)** contains the Q-Q plots of the *χ*^2^ goodness-of-fit statistic while the bottom row (d, e, f) contains the same Q-Q plot mapped to a normalized Z-statistic to improve the visibility of the left tail of the distribution. Independently on how count data are normalized, about 10% of the expression profiles show a substantial discrepancy to the NB distribution.

We assessed the goodness-of-fit of every expression profile in the LCL RNA-seq data to an NB distribution (see Methods) by means of quantile-quantile (Q-Q) plots (Figure 3) and about 10% of the genes show a substantial discrepancy with respect to the NB distribution in the counts (see right *y*-axis in Figure 3). Such a discrepancy is absent from data simulated from NB distributions with a similar number of genes including a small fraction of them changing between two conditions (Additional file 2: Figure S1).

This result suggests that NB distributions may be too restrictive for an important fraction of expression profiles in large RNA-seq data sets. Among the possible causes underlying the lack of fit of those genes to an NB distribution, a clear candidate is that the presence of many samples can potentially introduce features such as zero-inflation or heavy-tails (see Figure 1). So far, extensive biological replication in RNA-seq experiments has been the exception rather than the rule. However, it is becoming increasingly clear [15] that in the coming years larger RNA-seq data sets will be required to justify scientific conclusions and provide reproducible results. Therefore, we can expect to see more often gene expression profiles with emerging features, such as zero-inflation and heavy tails, that challenge RNA-seq methods based on the NB distribution.

We propose to address this problem by adopting the Poisson-Tweedie (PT) family of distributions [16] to model RNA-seq count data directly. PT distributions are described by a mean (*μ*), a dispersion (*ϕ*) and a shape (*a*) parameter (see Methods) and include Poisson and NB distributions, among others, as particular cases [16]. An important feature of this family is that, while the NB distribution only allows a quadratic mean-variance relationship, the PT distributions generalizes this relationship to any order [17]. We have implemented a maximum likelihood procedure for the estimation and simulation of these parameters from count data. These procedures are available in the tweeDEseq package through the functions mlePoissonTweedie(), dPT() and rPT().

Figure 1 illustrates the flexibility of the PT distribution to accurately fit different gene expression profiles obtained from the un-normalized LCL RNA-seq data set. Left and right panels correspond to female and male samples, respectively and each row corresponds to a different gene: *EEF1A2* (ENSG00000101210), *SCT* (ENSG00000070031) and *NLGN4Y* (ENSG00000165246), respectively. Among these three genes, only *NLGN4Y* has been reported in the literature to have sex specific expresssion, while the other two are likely to lack such property since *EEF1A2* is a housekeeping gene and *SCT* is an endocrine hormone peptide in chromosome 11 that controls secretions in the duodenum. Each plot shows the empirical cumulative distribution of observed counts as well as the parametric cumulative distributions obtained through the estimation of parameters of the methods compared in this paper under different configurations. Note that the estimated dispersion parameter *ϕ* is identical between the two sample groups for edgeR and DESeq (pooled) as these approaches estimate *ϕ* irrespective from the sample groups. The *P*-value for testing whether the data follow an NB distribution (*H*_0_:*a*=0), indicated above the legend, reveals that in several sample groups (panels a-c, e) this hypothesis is rejected (*P*<0.05). In those cases, methods based on the NB distribution produce dispersion parameters that do not fit the data as accurately as the PT distribution. More concretely, heavy-tails present in panels a,c severely hamper the estimation of the pure NB and the common dispersion. These can be improved using a parameter configuration more suited to large sample sizes. However, this results in a poor estimate of zero-inflation in panels c-e.

**Figure 4:**
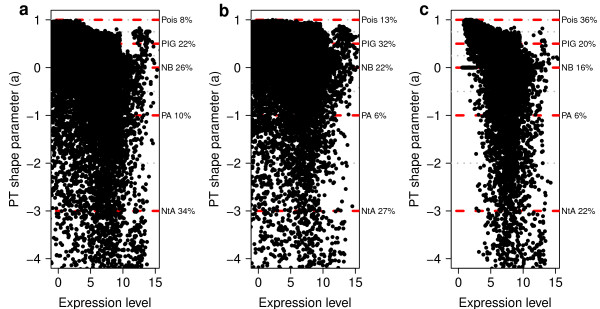
**Distribution of the Poisson-Tweedie shape parameter as function of the mean expression level.** Estimated Poisson-Tweedie shape parameter *a* as function of the mean expression level for each gene. Red dashed lines indicate the value of *a* corresponding to each specific distribution within the Poisson-Tweedie family, denoted by Pois (Poisson), PIG (Poisson-Inverse Gaussian), NB (negative binomial), PA (Pólya-Aeppli) and NtA (Neyman type A). The right *y*-axis indicates the percentage of genes around specific *a* values bounded by dotted grey lines. Data from Pickrell [12]*et al.* (2010) are shown without any normalization **(a)**, normalized with edgeR[2]**(b)**, and normalized with cqn[4]**(c)**.

The main difference between the PT and NB distributions lies in the additional “shape” parameter *a* of the PT distribution which provides further flexibility (see Methods). Using the LCL data processed with different normalization methods, we show in Figure 4 all values of the shape parameter *a* for every gene as function of its mean expression level, illustrating the huge variability of this parameter in RNA-seq count data. This wide range of values involves distinct possible distributional assumptions [16] beyond Poisson and NB, such as Poisson-Inverse Gaussian, Pólya-Aeppli or Neyman type A. Similarly to the MA-plots of Figure 2, the cqn normalization method seems to make the largest impact on count data and, in this case, on the shape parameter.

**Figure 5:**
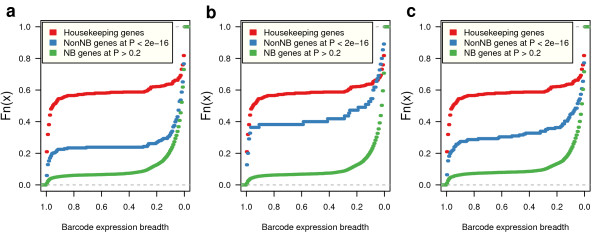
**Expression dynamics of genes with different count data distributions.** Empirical cumulative distributions of the breadth of expression estimated through the Barcode [18] database, for genes that do not reject the null hypothesis of a negative-binomial (NB) distribution in a test for the goodness of fit at *P*>0.2 (green lines), genes that do reject such a null hypothesis at *P*<2^−16^ (blue lines) and housekeeping genes retrieved from literature [19] (red lines). Data from Pickrell [12]*et al.* (2010) are shown without any normalization **(a)**, normalized with edgeR[2]**(b)** and normalized with cqn[4]**(c)**. These plots show that, independently of the normalization method, non-NB genes at such significance level of discrepancy with respect to the NB distribution approach closer the expression dynamics of housekeeping genes than genes with expression profiles following the NB distribution.

We have investigated whether this diversity of count distributions underlying RNA-seq data is related to different expression dynamics in genes. Using the test for the goodness of fit to an NB distribution (see Methods) we have considered as NB those genes that do not reject the null hypothesis at *P*>0.2 and as clear-cut non-NB genes those with *P*<2^−16^. By mapping all these genes to the Gene Expression Barcode catalog [18] (see Methods) we obtained an independent and unbiased estimation of their expression breadth. The results in Figure 5 suggest that the expression breadth of nonNB genes approaches that of housekeeping genes closer than NB genes do, irrespective of the normalization method.

In fact, Fisher’s exact tests for enrichment of non-NB genes among human housekeeping genes are significant (*P*<1.24^−6^) for every normalization method (see Additional file 2: Table S1). These observations suggest that genes with different expression dynamics can produce different count data distributions, and underscore the flexibility of the PT statistical model to capture these dynamics revealed by extensively-replicated RNA-seq experiments.

### Accurately testing differential expression

For the purpose of a DE analysis between two groups of samples, we have developed a two-sample PT-test for differences in means (see Methods) implemented through the function tweeDE() in the tweeDEseq package. We will assess the accuracy of this PT-based test using the LCL data as well as synthetic count data from two different simulation studies. The first simulation study with synthetic data provides an assessment of the type-I error rate under four different scenarios involving distinct count data distributions between sample groups (see Additional file 2: Table S2 for a description of them). Here we compare tweeDEseq with the configurations of edgeR and DESeq which are closer to a straightforward NB model. Additional file 2: Figures S2 to S5 show that tweeDEseq properly controls the nominal probability of a type-I error while edgeR, DESeq and non-parametric tests (U Mann-Withney and permutation) fail to do so when data are not simulated from NB distributions. As expected, these methods perform correctly when data are generated under an NB model (see Additional file 2: Figure S5) as expected. Additional file 2: Figure S6 also shows that in the calculation of very low *P*-values, tweeDEseq clearly outperforms permutations tests. In order to provide a practical recommendation on the minimum sample size required by tweeDEseq to yield accurate results we have estimated the probability of a type-I error across different sample sizes. Additional file 2: Figure S7 indicates that 15 samples per group should be sufficient for tweeDEseq to correctly control for a nominal significance level *α*=0.05.

In the second simulation study we have first assessed the accuracy of the *P*-value distribution under the null hypothesis of no differential expression with real RNA-seq data by making repeatedly two-sample group comparisons within males and within females samples such that we recreate the null hypothesis of no DE with real RNA-seq data and no DE gene should be expected to be found. The raw *P*-value distributions from such analysis should ideally be uniform.

**Figure 6:**
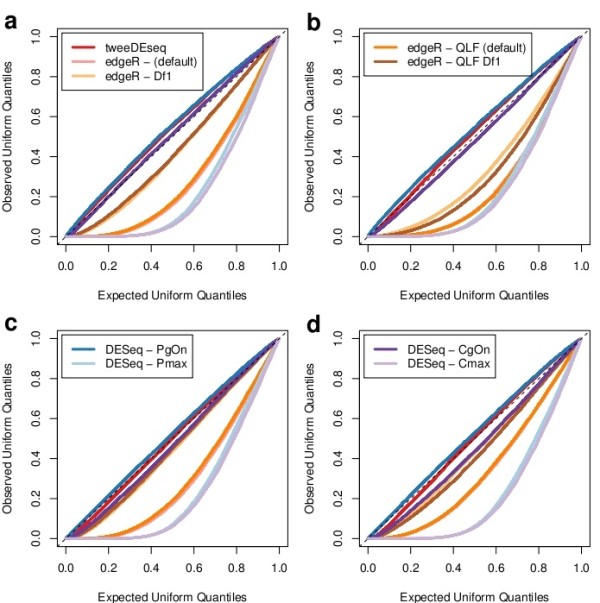
**Quantile-quantile (Q-Q) plots for the goodness-of-fit of null-hypothesis *P*-values to an uniform distribution.** Using the results displayed in Additional file 2: Figure S8 and performing as described by Leek *et al.* (2007) [20], for each gene, the distribution of *P*-values throughout the 100 simulations was tested for its goodness-of-fit to an uniform distribution using a Kolmogorov-Smirnov test. Q-Q plots in this figure show for all genes the resulting *P*-values of the previous test which, under the null hypothesis of no differential expression, should be uniformly distributed too and lead to lines lying on the diagonal. Panels **a-b** show results from female vs female comparisons and **c-d** from male vs male comparisons, while a,c correspond to un-normalized data and b,d to data normalized with the cqn[4]. The method introduced in this paper, tweeDEseq, is on average closer to the diagonal throughout the four simulations, closely followed by DESeq when sharingMode=~gene-est-only~ and either method=~per-condition~ or method=~pooled~.

We have formally tested this hypothesis for every gene by means of a Kolmogorov-Smirnov (KS) goodness-of-fit test to a uniform distribution and examine the resulting *P*-value distribution by means of Q-Q plots displayed in Figure 6. Under the null hypothesis that all genes are not DE, the KS *P*-values should lie along the diagonal of the Q-Q plot. The figure, however, shows large discrepancies to this criterion by some of the methods and configuration parameters, indicating that they may not be adequate for large RNA-seq data sets.

The method introduced in this paper, tweeDEseq, is consistently closer to the diagonal than every other method throughout the two male and female comparisons and the two normalization methods. More informally, the visual inspection of the histograms of raw *P*-values given in Additional file 2: Figure S8 also reveals that tweeDEseq provides *P*-value distributions closer to the uniform under the null hypothesis of no DE simulated from extensively replicated real RNA-seq data.

As other authors have shown, in the context of analysis of RNA-seq data with very limited sample size [8], small deviations from uniformity of *P*-values under the null hypothesis can substantially affect FDR estimates of DE genes. We have also assessed the calibration of *P*-values and false discovery rates (FDR) with synthetic count data of similar dimensions to the RNA-seq LCL data set, concretely with *p*=20,000 genes and *n*=70 samples. Working with this type of data allows to assess FDR estimates for a known subset of DE genes under a variety of simulated scenarios, which we defined by considering the combination of three different amounts of DE genes (100, 1000 and 2000) and three different magnitudes of fold-change (1.5, 2 and 4-fold). Similarly to [8], data were simulated from a hierarchical gamma-Poisson model with and without simulated library factors (see Methods).

**Figure 7:**
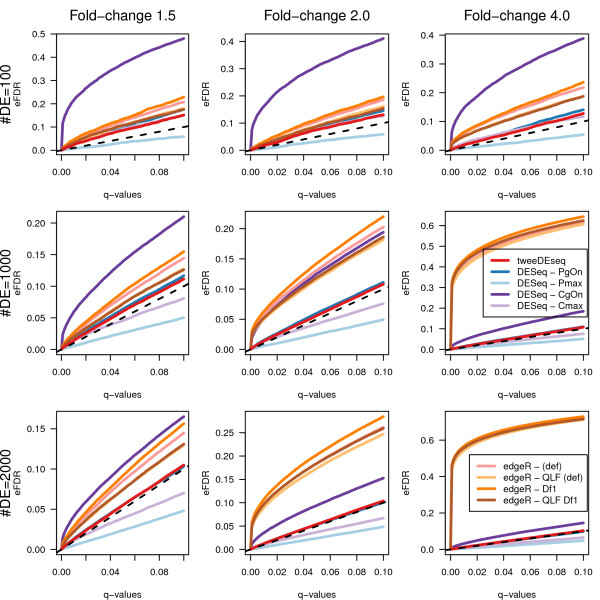
**Empirical FDR values for simulated data with constant library factors.** Empirical FDR values on the *y*-axis as function of nominal *q*-values on the *x*-axis calculated from data simulated with *p*=20,000 genes, *n*=70 samples and constant library factors. Each row and column corresponds, respectively, to a different number of DE genes and magnitude of the fold-change. The method introduced in this paper, tweeDEseq, is consistently closer to the diagonal than other methods throughout the different simulations.

**Figure 8:**
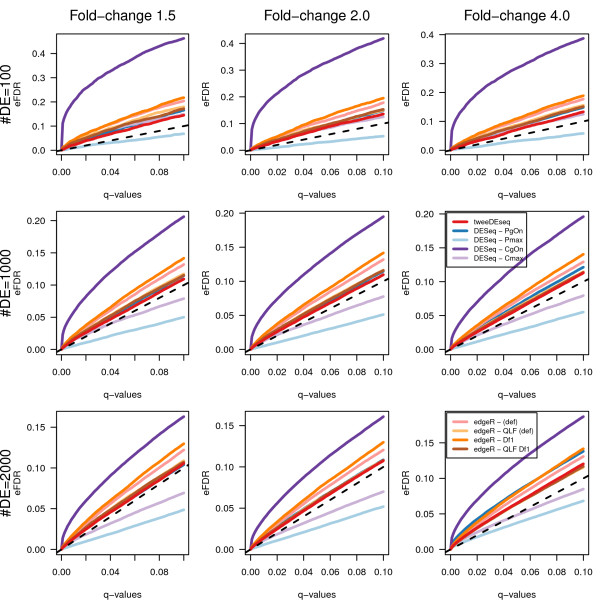
**Empirical FDR values for simulated data with variable library factors.** Empirical FDR values on the *y*-axis as function of nominal *q*-values on the *x*-axis calculated from data simulated with *p*=20,000 genes, *n*=70 samples and variable library factors. Each row and column corresponds, respectively, to a different number of DE genes and magnitude of the fold-change. The method introduced in this paper, tweeDEseq, is consistently closer to the diagonal than other methods throughout the different simulations.

From every simulated data set, raw *P*-values for the two-sample DE test were obtained with each method and configuration parameters. Using the qvalue Bioconductor package [21] we estimated *q*-values and the fraction of DE genes from each *P*-value distribution. Q-values provide a nominal estimation of the FDR for each gene and in Figures 7 and 8 we show the empirical FDR (eFDR) as a function of the nominal *q*-values for the simulations with constant and variable library factors, respectively. The dashed diagonal line indicates a correct calibration of *P*-values whose nominal FDR equals the observed eFDR. Lines above the diagonal correspond to liberal DE analysis methodologies and below to conservative ones.

To facilitate the comparison of methods across all simulated data sets we have calculated the mean squared error (MSE) between the eFDR and the nominal FDR and ranked the methods by increasing MSE. In Tables 2 and 3 we can find the MSE values and in Tables 4 and 5 the corresponding ranks of the methods according to the MSE values. As it follows from the rankings in Tables 4 and 5, tweeDEseq provides the best calibrated *P*-values in most of the simulated data sets.

**Table 2:** Mean squared error of false discovery rates under constant library factors

**#DE**	**Rank**	**1.5-fold change**		**2-fold change**		**4-fold change**	
		**MSE**	**Method**	**MSE**	**Method**	**MSE**	**Method**
	1	0.050	DESeq - Pmax	0.050	DESeq - Pmax	0.031	tweeDEseq
	2	0.122	tweeDEseq	0.053	tweeDEseq	0.037	DESeq - Cmax
	3	0.155	DESeq - Cmax	0.065	DESeq - Cmax	0.070	DESeq - Pmax
	4	0.257	DESeq - PgOn	0.104	DESeq - PgOn	0.072	DESeq - PgOn
100	5	0.306	edgeR - QLF Df1	0.138	edgeR - QLF Df1	0.430	edgeR - QLF Df1
	6	0.313	edgeR - QLF (def)	0.177	edgeR - QLF (def)	0.452	edgeR - QLF (def)
	7	0.558	edgeR - (def)	0.336	edgeR - (def)	0.790	edgeR - (def)
	8	0.755	edgeR - Df1	0.431	edgeR - Df1	0.957	edgeR - Df1
	9	9.688	DESeq - CgOn	6.232	DESeq - CgOn	5.133	DESeq - CgOn
	1	0.008	tweeDEseq	0.004	tweeDEseq	0.004	tweeDEseq
	2	0.008	DESeq - Cmax	0.008	DESeq - PgOn	0.007	DESeq - PgOn
	3	0.016	DESeq - PgOn	0.015	DESeq - Cmax	0.014	DESeq - Cmax
	4	0.043	edgeR - QLF Df1	0.087	DESeq - Pmax	0.081	DESeq - Pmax
1000	5	0.045	edgeR - QLF (def)	0.413	edgeR - QLF (def)	0.429	DESeq - CgOn
	6	0.082	DESeq - Pmax	0.459	edgeR - QLF Df1	21.358	edgeR - QLF (def)
	7	0.105	edgeR - (def)	0.532	DESeq - CgOn	22.208	edgeR - (def)
	8	0.155	edgeR - Df1	0.639	edgeR - (def)	23.401	edgeR - QLF Df1
	9	0.735	DESeq - CgOn	0.835	edgeR - Df1	25.004	edgeR - Df1
	1	0.002	DESeq - PgOn	0.001	DESeq - PgOn	0.000	DESeq - PgOn
	2	0.002	tweeDEseq	0.001	tweeDEseq	0.001	tweeDEseq
	3	0.025	DESeq - Cmax	0.031	DESeq - Cmax	0.036	DESeq - Cmax
	4	0.053	edgeR - QLF (def)	0.090	DESeq - Pmax	0.093	DESeq - Pmax
2000	5	0.056	edgeR - QLF Df1	0.183	DESeq - CgOn	0.140	DESeq - CgOn
	6	0.093	DESeq - Pmax	1.444	edgeR - QLF (def)	34.551	edgeR - QLF (def)
	7	0.113	edgeR - (def)	1.702	edgeR - QLF Df1	35.365	edgeR - (def)
	8	0.169	edgeR - Df1	1.724	edgeR - (def)	35.468	edgeR - QLF Df1
	9	0.271	DESeq - CgOn	2.219	edgeR - Df1	36.929	edgeR - Df1

Data in this table correspond to the mean squared error (MSE) values between the empirical false discovery rates (eFDR) and the nominal *q*-values obtained from the simulation study shown in Figure 7 in which library factors were held constant.

**Table 3:** Mean squared error of false discovery rates under variable library factors

**#DE**	**Rank**	**1.5-fold change**		**2-fold change**		**4-fold change**	
		**MSE**	**Method**	**MSE**	**Method**	**MSE**	**Method**
	1	0.030	DESeq - Pmax	0.059	DESeq - Cmax	0.046	tweeDEseq
	2	0.099	tweeDEseq	0.064	tweeDEseq	0.055	DESeq - Pmax
	3	0.189	DESeq - Cmax	0.072	DESeq - Pmax	0.057	DESeq - Cmax
	4	0.194	DESeq - PgOn	0.116	DESeq - PgOn	0.106	DESeq - PgOn
100	5	0.258	edgeR - QLF Df1	0.129	edgeR - QLF Df1	0.124	edgeR - QLF Df1
	6	0.348	edgeR - QLF (def)	0.129	edgeR - QLF (def)	0.153	edgeR - QLF (def)
	7	0.581	edgeR - (def)	0.273	edgeR - (def)	0.290	edgeR - (def)
	8	0.667	edgeR - Df1	0.420	edgeR - Df1	0.380	edgeR - Df1
	9	8.882	DESeq - CgOn	6.429	DESeq - CgOn	5.217	DESeq - CgOn
	1	0.005	tweeDEseq	0.006	tweeDEseq	0.008	tweeDEseq
	2	0.009	DESeq - Cmax	0.012	DESeq - Cmax	0.010	DESeq - Cmax
	3	0.013	DESeq - PgOn	0.012	DESeq - PgOn	0.011	edgeR - QLF Df1
	4	0.016	edgeR - QLF Df1	0.016	edgeR - QLF Df1	0.013	edgeR - QLF (def)
1000	5	0.019	edgeR - QLF (def)	0.017	edgeR - QLF (def)	0.024	DESeq - PgOn
	6	0.054	edgeR - (def)	0.051	edgeR - (def)	0.045	edgeR -(def)
	7	0.083	DESeq - Pmax	0.082	DESeq - Pmax	0.067	DESeq - Pmax
	8	0.087	edgeR - Df1	0.083	edgeR - Df1	0.077	edgeR - Df1
	9	0.700	DESeq - CgOn	0.545	DESeq - CgOn	0.529	DESeq - CgOn
	1	0.003	tweeDEseq	0.004	tweeDEseq	0.005	DESeq - Cmax
	2	0.003	DESeq - PgOn	0.006	edgeR - QLF Df1	0.017	edgeR - QLF (def)
	3	0.006	edgeR - QLF Df1	0.006	edgeR - QLF (def)	0.018	edgeR - QLF Df1
	4	0.007	edgeR - QLF (def)	0.007	DESeq - PgOn	0.023	tweeDEseq
2000	5	0.025	DESeq - Cmax	0.024	DESeq - Cmax	0.029	DESeq - Pmax
	6	0.028	edgeR - (def)	0.026	edgeR - (def)	0.053	edgeR - (def)
	7	0.049	edgeR - Df1	0.047	edgeR - Df1	0.088	edgeR - Df1
	8	0.091	DESeq - Pmax	0.077	DESeq - Pmax	0.092	DESeq - PgOn
	9	0.267	DESeq - CgOn	0.238	DESeq - CgOn	0.465	DESeq - CgOn

Data in this table correspond to the mean squared error (MSE) values between the empirical false discovery rates (eFDR) and the nominal *q*-values obtained from the simulation study shown in Figure 8 in which library factors were variable.

**Table 4:** Rankings of methods by the mean squared error of false discovery rates under constant library factors

**Method**	**#DE = 100**			**#DE = 1000**			**#DE = 2000**		
	**1.5 FC**	**2 FC**	**4 FC**	**1.5 FC**	**2 FC**	**4 FC**	**1.5 FC**	**2 FC**	**4 FC**
tweeDEseq	2	2	1	1	1	1	2	2	2
DESeq - PgOn	4	4	4	3	2	2	1	1	1
DESeq - Pmax	1	1	3	6	4	4	6	4	4
DESeq - CgOn	9	9	9	9	7	5	9	5	5
DESeq - Cmax	3	3	2	2	3	3	3	3	3
edgeR - (def)	7	7	7	7	8	7	7	8	7
edgeR - QLF (def)	6	6	6	5	5	6	4	6	6
edgeR - Df1	8	8	8	8	9	9	8	9	9
edgeR - QLF Df1	5	5	5	4	6	8	5	7	8

Data in this table correspond to the rankings of every method by the mean squared error (MSE) values shown in Table 1.

**Table 5:** Rankings of methods by the mean squared error of false discovery rates under variable library factors

**Method**	**#DE = 100**			**#DE = 1000**			**#DE = 2000**		
	**1.5 FC**	**2 FC**	**4 FC**	**1.5 FC**	**2 FC**	**4 FC**	**1.5 FC**	**2 FC**	**4 FC**
tweeDEseq	2	2	1	1	1	1	1	1	4
DESeq - PgOn	4	4	4	3	3	5	2	4	8
DESeq - Pmax	1	3	2	7	7	7	8	8	5
DESeq - CgOn	9	9	9	9	9	9	9	9	9
DESeq - Cmax	3	1	3	2	2	2	5	5	1
edgeR - (def)	7	7	7	6	6	6	6	6	6
edgeR - QLF (def)	6	6	6	5	5	4	4	3	2
edgeR - Df1	8	8	8	8	8	8	7	7	7
edgeR - QLF Df1	5	5	5	4	4	3	3	2	3

Data in this table correspond to the rankings of every method by the mean squared error (MSE) values shown in Table 2.

**Figure 9:**
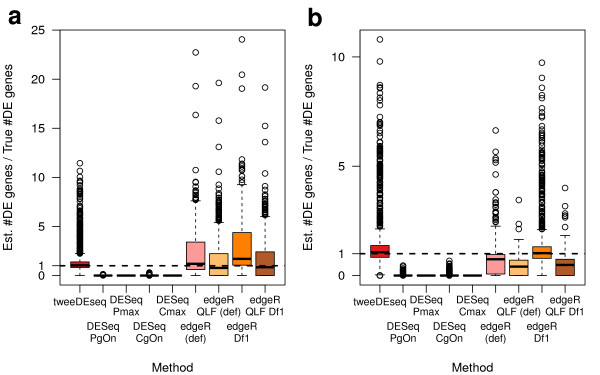
**Estimation of the number of differentially expressed (DE) genes from simulated data.** Boxplots of ratios of estimated to true numbers of DE genes obtained from data simulated from a hierarchical gamma-Poisson model with constant **(a)** and variable **(b)** library factors. This figure summarizes the results in Additional file 2: Figures S9 and S10 reporting estimated numbers of DE genes under different simulated scenarios of number or true DE genes and fold-change. The horizontal dash line at ratio one corresponds to the best performance where the estimated number of DE genes matches the true number.

The previous calculations of *q*-values with the qvalue package [21] provide us also with estimates ${\hat{\pi }}_{0}$ of the fraction of genes under the null hypothesis of no differential expression. This, in turn, allows one to derive an estimated number of DE genes as $p(1-{\hat{\pi }}_{0})$ with *p* being the total number of genes. In principle, more precise *P*-values both under the null and the alternative hypotheses should provide more accurate estimates of the number of DE genes. We show such an assessment for the previous simulations in Additional file 2: Figures S9 and S10. To summarize those results we have divided each estimate of the number of DE genes by their actual simulated number of DE genes and aggregate those ratios throughout the different simulation scenarios to ease the comparison among the methods. We find this comparison in Figure 9 and it follows that tweeDEseq produces *P*-values that lead to the most accurate estimation of the number of DE genes, closely followed by edgeR with prior.df=1 when library factors are not held constant. In both settings, DESeq leads to extremely conservative estimates of the number of DE genes.

### Identification of sex-specific gene expression in lymphoblastoid cell lines

Assessing performance of DE analysis methods without using simulated data is a challenging problem due to the difficulty of knowing or ensuring the exact differential concentration of RNA molecules in the analysed samples. In this respect, sex-specific expression constitutes a useful system to assess the accuracy of DE detection methods due to the vast literature on genes contributing to gender-specific traits. For this reason, in order to illustrate the accuracy of tweeDEseq with real RNA-seq data, we have searched for genes changing significantly their expression between female and male individuals of the RNA-seq experiments on LCLs analyzed in this paper. Again, we have compared different normalization procedures and parameter configurations of edgeR and DESeq. Next to considering the raw un-normalized data and the data normalized with cqn, TMM normalization was used for edgeR and tweeDEseq, while DESeq was used with its own normalization method. We have used a single significance cutoff of FDR <0.1 at which genes were called DE. Since LCLs come from a non-sexually dimorphic tissue and are outside their original biological context, the fraction of sex-specific expression changes we could expect should be rather small.

In an attempt to verify the accuracy of these lists of DE genes between female and male individuals, we searched for genes reported in the literature to be potential contributors to sexually dimorphic traits. This list of genes with documented sex-specific expression was obtained from genes in chromosome X that escape X-inactivation [22] and from genes in the male-specific region of the Y chromosome [23] (see Methods). This resulted in a gold-standard set of 95 genes mapping to Ensembl Gene Identifiers (release 63), which we shall denote by XiE and MSY genes, depending on their origin. For every predicted set of DE genes by each combination of DE detection method and normalized data set, we calculated precision and recall with respect to the gold-standard, and the *F*-measure which summarizes the trade-off between these two diagnostics.

**Figure 10:**
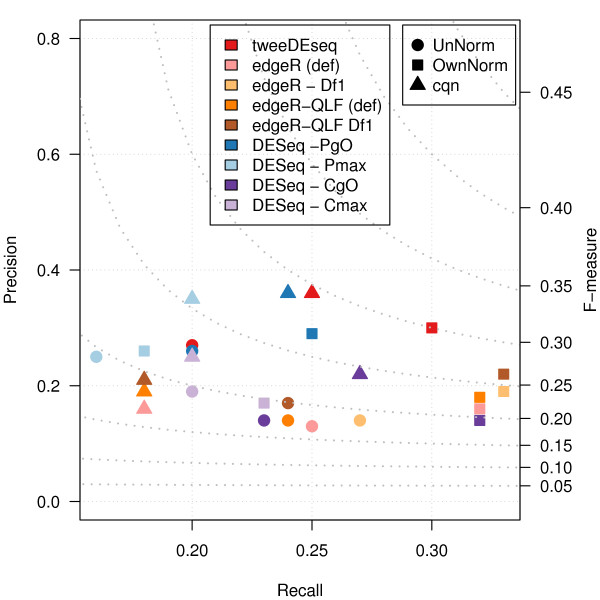
**Precision and recall comparison on the LCL RNA-seq data.** Precision (*y*-axis) and recall (*x*-axis) values for genes called DE at 1% FDR by different DE detection methods and configuration parameters. The right *y*-axis indicates values of the *F*-measure shown by dot lines. As the figure shows, tweeDEseq provides higher *F*-measure values than other methods indicating a better precision-recall tradeoff.

In Figure 10 we can see that tweeDEseq provides better performance than the other competing methods under different parameter configurations. The improvement is small with respect to the second best-performing method and parameter configuration but we would like to emphasize that tweeDEseq does not require any informed decision on a parameter configuration, as opposed to edgeR and DESeq. To assess the robustness of this figure, we have run this comparative assessment with a more stringent filter on lowly expressed genes and, as Additional file 2: Figure S11 shows, tweeDEseq keeps performing better than the other methods, this time however only when data are normalized.

In Additional file 2: Table S3 we report the complete list of 55 DE genes detected by tweeDEseq from the data normalized with cqn, which is when it yields the best precision-recall tradeoff. More than a half of genes in this list (32) are located in either the X or Y chromosomes and where the first 10 with largest fold-change contain 7 from the gold-standard set of MSY and XiE genes. Among the other 3, we find *TTTY15*, a testis-specific non-coding transcript from the Y chromosome and the other two lack functional annotation in Ensembl release 63.

### Reproducibility with respect to microarray data

The YRI LCL samples we have analyzed have been previously assayed using microarray chips [24] and this enables a comparison between the gene expression read out of both technologies. In particular, we wanted to assess the degree of reproducibility of the significance levels of DE. While there may be many aspects from both technologies that can potentially bound the extent to which we can reproduce rankings of DE, we postulate that more accurate *P*-values in DE genes should lead to higher reproducibility of significance levels of DE genes.

With this purpose, we applied limma[25] on the microarray data and called genes DE at 10% FDR, just as we did with RNA-seq data, and then compared the − log10 units of the raw *P*-values from DE genes called in RNA-seq by each DE detection method to the − log10*P*-value units from genes called DE by limma. In Additional file 2: Figure S12 we show this comparison for every gene that is called DE either by limma in microarray data or by the other compared method in RNA-seq data. Although we can observe a significant linear relationship between *P*-values in every compared method, the low fraction of variability explained by the fitted linear model (*R*^2^<0.25) in every of those comparisons indicates a rather poor level of reproducibility for every method.

A closer look to genes in that figure indicates that the lack of reproducibility mostly comes from genes called DE by one method and technology but not by the other (dots close to zero in either the *x* or the *y*-axis). There may be many reasons, unrelated to the DE detection method itself, why a gene is not called simultaneously DE in two completely independent RNA-seq and microarray experiments on the same biological material, such as different isoforms being probed in the microarray and summarized in RNA-seq or differences in sample preparation. Therefore, for our current goal of assessing reproducibility of DE detection methods, we believe it makes sense to restrict this comparison to those genes that are called DE by both, limma in microrray data and the corresponding method in RNA-seq data.

**Figure 11:**
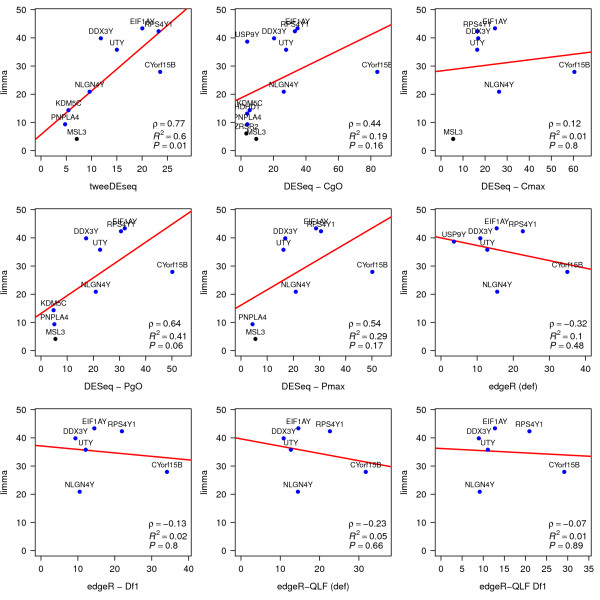
**Reproducibility of differential expression (DE) between microarray and RNA-seq.** Raw *P*-values of differential expression in − log10 scale for DE genes called at 10% FDR by both, limma (*y*-axis), from microarray data, and the other compared DE detection method applied on RNA-seq data (*x*-axis). A regression line is depicted in red. On the bottom-right corner of each panel, *ρ* indicates the Pearson correlation whereas *R*^2^ and *P* indicate, respectively, the coefficient of determination and *P*-value of the test for zero regression coefficient, of the − log10*p*-values of limma as function of those from the compared RNA-seq method. Only tweeDEseq provides a significant (*p*<0.05) level of reproducibility between *P*-values of DE genes reported by both, limma on microarray data and the compared RNA-seq method, attaining also the highest *ρ* and *R*^2^ values. Blue dots indicate genes with documented sex-specific expression.

We can find this restricted comparison in Figure 11 which reveals that in this case only tweeDEseq attains a significant (*P*<0.05) linear fit with respect to the *P*-values from limma with a level of reproduciblity (*R*^2^=0.6) substantially larger (46% increase) than the second best method (DESeq - PgO) with *R*^2^=0.41.

**Figure 12:**
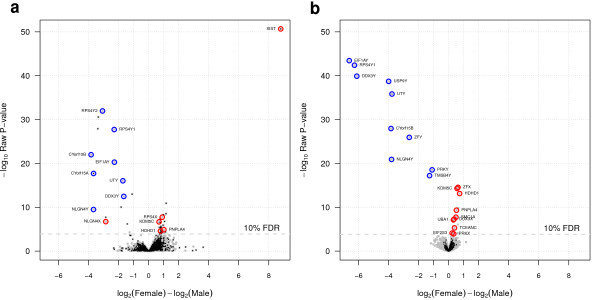
**Comparison of DE analyses between microarray and RNA-seq.** Volcano plots of DE analyses performed on matching LCL samples profiled with RNA-seq **(a)** and gene expression microarrays **(b)**. The *x*-axis corresponds to log2 fold-changes between female and male individuals while the *y*-axis corresponds to − log10*p*-value of significance. RNA-seq data were analysed with tweeDEseq while microarray data were analysed with limma. Grey dots indicate genes common to both, the RNA-seq and the microarray gene expression matrices, while black dots indicate genes occurring exclusively in one of the two data sets. Blue and red circles indicate genes documented in the literature with sex-specific expression, concretely belonging to the male-specific region of chromosome Y and escaping X-chromosome inactivation in females, respectively.

Finally, we have carried out a comparison between the entire output of DE genes obtained with tweeDEseq in RNA-seq data with the entire output DE genes obtained with limma in microarray data. In Figure 12 we show the resulting volcano plots where we have highlighted with black dots those genes that are exclusively profiled by each technology. As the figure suggests, many more of these genes occur in RNA-seq than in microrray, one remarkable case being the *XIST* gene which shows the largest fold-change and significance level and corresponds to the X-inactive specific non-coding RNA gene which acts as one of the key regulators in silencing one of the copies of chromosome X in females. Blue and red circles denote MSY and XiE genes, respectively. As expected, all MSY and XiE DE genes report significantly higher expression in males and females, respectively, except for the XiE gene *NLGN4X* in RNA-seq, likely due to low expression from the inactive X chromosome in female samples [26]. Surprisingly the volcano plots show that limma on this microarray data set is able to detect a few more such genes than tweeDEseq on RNA-seq data. Last, but not least, an important difference between the volcano plots of Figure 12 is the fact that expression changes larger than 2-fold in these microarray data are nearly synonymous of statistical significance while with RNA-seq a sizeable fraction of genes with 2-fold or larger changes show very poor significance levels. This is likely due to the larger variability of gene expression measurements in RNA-seq experiments with many samples and underscores the importance of using methods that properly assess the statistical significance of the observed changes.

## Conclusions

The increased amount of biological variability revealed by extensive replication in RNA-seq experiments brings new challenges to the task of identifying genes whose change in expression is both, biologically and statistically significant. In microarray data, large fold-changes derived from large data sets were nearly synonymous of statistical significance. The volcano plots in Figure 12 and the examples of specific genes in Figure 1 illustrate why this is not true anymore with RNA-seq count data. Those figures unveil that one of these new challenges is to distinguish statistically significant changes among those that are already large in magnitude. In this paper we provide an approach to this problem by using the PT family of distributions, showing that it captures a much richer diversity of expression dynamics in RNA-seq count data than the statistical models based in the NB distributions alone (see Figures 4 and 5). We have implemented a two-sample PT-test in a software package for R, called tweeDEseq, for detecting DE genes and demonstrated with simulations that produces more accurate *P*-value distributions that lead to better calibrated *q*-values and FDR estimates.

We have made an attempt to assess DE detection accuracy with real RNA-seq data by comparing male and female LCL samples normalized with three different methods and comparing the results to a gold-standard set of genes with documented sex-specific expression. This assessment also shows that tweeDEseq provides a better precision-recall tradeoff than the compared NB-based methods (see Figure 10 and Additional file 2: Figure S11). We have also made a comparison with matching samples hybridised on microarray chips which allowed us to verify that tweeDEseq yields a higher degree of reproducibility of significance levels with respect to microrray data.

All these different comparative assessments have been performed against two of the most widely currently used methods for DE analysis of RNA-seq data, edgeR and DESeq, under four different parameter configurations each, since their default parametrisation is tailored towards very limited sample size. Making an informed decision on what is the most appropriate setup is not trivial for the non-expert user and, for this reason, it is important to underscore that tweeDEseq is competitive with all of the methodologies that follow from the different configurations of edgeR and DESeq without the need to set a single parameter.

The fact that the volcano plots from tweeDEseq and limma, derived from RNA-seq and microarray data, reveal that limma is able to find a larger number of DE genes from the gold-standard, suggests a long way still ahead of us to fully exploit the RNA-seq technology for DE. Not only regarding experimental aspects, but also statistical ones such as properly detecting and adjusting for unwanted sources of non-biological variability, for which there is currently no well-established available techniques, as in the case of microarray data.

Other applications of high-throughput sequencing technology that output counts of molecules, like in Copy Number Variation (CNV) analysis, could potentially benefit of models based on the PT-distribution. It is our perception that richer count data models of this kind will become increasingly necessary to draw accurate conclusions from data as technology brings us closer the actual biology of the cell.

## Methods

### Pre-processing of RNA-seq data

We have analyzed data from Pickrell *et al.* (2010) [12] that sequenced RNA from LCLs in 69 Nigerian (YRI) [12] individuals. Raw reads were downloaded from http://eqtl.uchicago.edu/RNA_Seq_data/unmapped_reads and pre-processed using the GRAPE pipeline [27]. This pipeline consists of first mapping the reads to the human genome version hg19 using the GEM mapper software [28]. Second, mapped reads were summarized into gene-level counts according to the GENCODE annotation version 3c [29] with Ensembl release 63 gene identifiers, by selecting those reads that mapped either completely within an exon or spanning a junction. This resulted in an initial table of counts of 38,415 Ensembl genes. This table of counts form part of the experimental data package tweeDEseqCountData available at http://www.bioconductor.org under the name pickrell1.

The table of counts was filtered to discard lowly expressed genes by keeping only those with an average of more than 0.1 counts per million (CPM) throughout the samples. The results shown in Additional file 2: Figure S11 were obtained by applying a more stringent minimum cutoff of 0.5 CPM. When we applied a normalization method that adjusted for gene length and G+C content (see below), genes without this information were also discarded. When the minimum CPM was 0.1, then 31,226 genes were kept when no normalization method or edgeR-TMM was applied and when cqn was applied then 27,438 were kept (see pg. 5 and 6 from Additional file 1). When the minimum CPM was 0.5 then these numbers decreased to 19,166 and 18,009 genes, respectively.

Three approaches to normalizing the table of counts from the LCL data have been considered. The first one is to work with the initial table of raw counts without any kind of normalization, the second one is to apply TMM [2] normalization as implemented in the edgeR[30] package, the third one is to use the methodology implemented in the cqn[4] Bioconductor package which adjusts for sample-specific effects of gene length and G+C content of every gene. When using the DESeq method for DE analysis in the LCL samples, the TMM normalization procedure was replaced by its own normalization procedure.

Raw counts were transformed into filtered and normalized counts for the purpose of producing MA-plots (Figure 2), assessing goodness of fit to the NB distribution (Figure 3), examining the relationship between mean expression level and the shape parameter of the PT distribution (Figure 4) and doing DE analysis with tweeDEseq. In the case of DESeq raw counts were transformed into normalized counts only when used with the cqn normalization method.

In the case of edgeR-TMM normalization, counts were transformed following the steps that the function exactTest() in edgeR takes: calculate normalization factors with the TMM method (calcNormFactors()), estimate effective library sizes and adjust counts to effective library sizes obtaining non-integer normalized pseudocounts (equalizeLibSizes()) which were subtracted by 0.5 and then raised to the smallest integers not less than these pseudocounts (ceiling()). These steps are written together in the function normalizeCounts() from the tweeDEseq package.

In the case of cqn, normalization offsets are calculated by the function cqn() as log2 RPMs, which are added to original raw log2 RPMs. These are rolled back to absolute numbers and “unlogged” obtaining non-integer normalized pseudocounts which, analogously to the edgeR-TMM case, were subtracted by 0.5 and then raised to the smallest integers not less than these pseudocounts (ceiling()). The rationale behind subtracting 0.5 to the pseudocounts instead of directly truncating or raising to the next integer value, is to try to approach as much as possible the correct proportion of zero counts in the normalized data.

However, when performing DE analysis with edgeR, or with DESeq and its own normalization procedure, the specific recommendations made by the corresponding software authors were followed. More concretely, raw counts were not transformed in order to preserve their sampling properties and normalization adjustments entered the DE analysis through the corresponding normalization factors and offsets arguments within the functions that test for DE (see scripts for details in Additional file 1).

### Pre-processing of microarray data

The microarray LCL data from [24] was processed from the raw CEL files available at http://www.ncbi.nlm.nih.gov/geounder accession GSE7792. Firstly, we only considered YRI samples. Secondly, data was processed using the Bioconductor oligo package. Quality assessment was performed by calculating NUSE and RLE diagnostics (Bolstad et al., 2005) and discarding those samples that either of the two reported diagnostics was considered below a minimum quality threshold. Third, using the RMA algorithm (Irizarry et al., 2003) implemented in the rma() function from the oligo package with argument target=~core~, expression values were background corrected, normalized and summarized into Affymetrix transcript clusters. Fourth, most samples formed part of family trios and only samples belonging to father or mother were kept. Fifth, using the getNetAffx() function from the oligo package, Ensembl Transcript identifiers well obtained for each Affymetrix transcript cluster identifier. Sixth, using the bioconductor package biomaRt, Ensembl Transcript identifiers were translated into Ensembl Gene identifiers, resolving multiple assignments by keeping the Ensembl Gene identifier that had a match in the Ensembl Gene identifiers forming the table of counts of the [12] RNA-seq data, or choosing one arbitrarily, otherwise. Seven, duplicated assignments of the same Ensembl Gene identifier to multiple Affymetrix transcript cluster identifiers were resolved by keeping the transcript cluster with largest expression variability measured by its interquartile range (IQR).

At this point an expression data matrix of 16,323 Ensembl Genes by 74 samples was obtained and using the scanning date of each CEL file, samples were grouped into 5 batches, out of which one containing only three male samples was discarded leaving a total of 71 samples distributed into 4 balanced batches across gender. Batch effect was removed by using the QR-decomposition method implemented in the removeBatchEffect() function from the Bioconductor package limma[25] while keeping the sex-specific expression effect by setting the gender sample indicator variable within the design matrix argument. Finally, samples and genes were further filtered to match those from the RNA-seq table of counts.

### Matching RNA-seq and microarray expression data matrices

To perform the analyses summarized in Figure 11 and Additional file 2: Figure S12 we further filtered the previously pre-processed RNA-seq and microarray gene expression matrices to match both Ensembl Gene identifiers and individual HapMap identifiers. This resulted in two gene expression data matrices of equal dimension with 15,194 genes and 36 samples. We only considered the RNA-seq data normalized with the cqn package.

To perform the analyses summarized in Figure 12 we built two other gene expression data matrices where, as before, samples were restricted to those 36 that matched between RNA-seq and microarray data but genes were not, leading to a RNA-seq and microarray gene expression data matrices of 27,438 and 16,323 Ensembl Genes by 36 samples, respectively. Genes were not matched since the purpose of these analyses was to gather insight into the differences and challenges in detecting DE genes using RNA-seq with respect to microarray gene expression data with many replicates.

### Functional annotations

Functional annotations for Ensembl genes forming the tables of counts, were retrieved from http://jun2011.archive.ensembl.org with R and the biomaRt Bioconductor package. Gene length and G+C content annotations, used with the cqn normalization method, were obtained by downloading all human cDNAs from http://ftp.ensembl.org/pub/release-63/fasta/homo_sapiens/cdna/Homo_sapiens.GRCh37.63.cdna.all.fa.gz and calculating the length and G+C content of the longest cDNA for each Ensembl gene.

The gold-standard list of genes with sex-specific expression was built with genes reported in the literature that, in one hand, escape chromosome X inactivation [22] and, on the other hand, belong to the male-specific region of chromosome Y [23]. In both cases, gene symbols were first translated into Ensembl gene identifiers and then further filtered to keep only those included in the set of Ensembl gene identifiers release 63. This resulted in a gold-standard list of 95 genes with sex-specific expression.

The list of housekeeping genes was retrieved from the literature [19] and mapped to Ensembl genes release 63, resulting in a final set of 669 housekeeping genes. The expression breadth reported in Figure 5 was obtained through the Barcode Gene Expression catalog [18] which uses information from 18,656 publicly available microarray samples from 131 tissue types, of the HG-U133 Plus 2.0 Affymetrix chip, to estimate the proportion of tissue types in which a given probeset is expressed in more than half the samples. After discarding unreliable probes (annotated with high-entropy in the catalog), we use these values as surrogates for expression breadth by mapping Affymetrix probeset identifiers to the genes in our table of counts through the hgu133plus2.db Bioconductor annotation package, leading to 16,292 genes with expression breadth values. When two or more probesets mapped to the same gene, the maximum value was taken for that gene.

All these functional data are included in the experimental data package tweeDEseqCountData available at http://www.bioconductor.org under the keywords annotEnsembl63, genderGenes and hkGenes.

### Poisson-Tweedie distributions

Poisson-Tweedie (PT) distributions have been studied by several authors [31-34] and unify several over-dispersed count data distributions (see Figure one in [34]). This family of distributions can be defined by a probability generating function and mass probabilities have to be computed using a recursive algorithm [31,34]. El-Shaarawi *et al.* (2011) [34] compared different recursions and parameterizations of this family providing an algorithm to compute the PT probability distribution function. In the R package tweeDEseq we have developed a fast implementation, written in the C programming language, of this recursive algorithm. We briefly describe here the PT family of distributions as well as how we have used it to analyze RNA-seq count data in the context of a differential expression (DE) analysis.

Following El-Shaarawi *et al.* (2011) [34], let *Y*∼PT(*a*,*b*,*c*) be a PT random variable with vector of parameters *θ*=(*a*,*b*,*c*)^*T*^ defined in the domain

(1) Θ=(−∞,1]×(0,+∞)×[0,1).

The PT random variable *Y* has a probability generating function (pgf) of the form:

(2) $$G_{Y}(y|a,b,c)=\exp\left\{\frac{b}{a}\left(\right){\left(1-c\right)}^{a}-{\left(1-\text{cy}\right)}^{a}))\right\}\;,$$

when *a*≠0, while when *a*=0, then:

(3) $$\underset{a\to 0}{\text{lim}}G_{Y}(y|a,b,c)={\left[\frac{\left(1-c\right)}{\left(1-\text{cy}\right)}\right]}^{b}\;.$$

Using this parameterization, the following recursive algorithm can be used to compute the PT probability distribution function [34]:

(4) $$\;p_{0}=\left\{\begin{array}{ll} e^{b[{\left(1-c\right)}^{a}-1]/a}, & a\neq 0,\\ {\left(1-c\right)}^{b}, & a=0, \end{array}\right)$$

(5) $$\begin{array}{l} \;p_{1}={\text{bcp}}_{0},\;p_{k+1}=\frac{1}{k+1}\left({\text{bcp}}_{k}+\overset{k}{\underset{j=1}{\sum }}{\text{jr}}_{k+1-j}p_{j}\right),\\ k=1,2,\ldots \end{array}$$

where

(6) $$r_{1}=(1-a)c,\;r_{j+1}=\left(\frac{j-1+a}{j+1}\right){\text{cr}}_{j},\;j=1,2,\ldots$$

and *p*_*i*_ denotes the probability of observing *i* counts.

For the sake of interpretability, we reparameterize *θ*=(*a*,*b*,*c*) to *θ*=(*μ*,*ϕ*,*a*), where *μ* denotes the mean, *ϕ*=*σ*^2^/*μ* is the dispersion index (*σ*^2^ is the variance), and *a* the shape parameter that is used to define some count data distributions that are particular cases of PT such as Poisson or negative binomial. The relationship between both parameterizations is the following:

(7) $$c=\frac{\phi -1}{\phi -a},\;b=\frac{\mu {\left(1-a\right)}^{\left(1-a\right)}}{(\phi -1){\left(d-a\right)}^{-a}}\;.$$

The PT model includes not only Poisson (*a*=1) and negative binomial (NB) (*a*=0) but also other distributions that have been used to analyze count data such as Poisson-Inverse Gaussian (PIG) ($a=\frac{1}{2}$), Pólya-Aeppli (P-A) (*a*=−1) or Neyman type A (*a*→−*∞*). Therefore, the PT distribution family unifies several diverse count data distributions, including different overdispersed distributions such as NB or PIG. These distributions can model different scenarios as, for instance, a RNA-seq expression profile with a wide dynamic range leading to a heavy tail in the distribution. In such a case, PIG has a heavier tail than NB and this would make it more appropriate for such a gene. Note that an extremely heavy tail implies overdispersion, but the converse does not hold; hence the NB distribution is not adequate to model RNA-seq expression profiles of genes with a wide dynamic range due to their intrinsic biological variability [15].

Given a certain parameterization Kokonendji *et al.* (2004) [17] prove that the mean-variance relationship for the PT family can be expressed as:

(8) $$\sigma^{2}=\mu \left(1+\mu^{p-1}\exp\left\{(2-p)\Phi_{p}\right\}\right)$$

where *p* is the shape parameter of that specific parameterization. It follows that, whereas the NB distribution is only able to capture a quadratic mean-variance relationship, the PT family is able to generalize this relationship to any order. As a result, it is more convenient to use the PT model when dealing with count data which presents variable overdispersion.

### Parameter estimation for Poisson-Tweedie distributions

We need to estimate the parameter vector $\hat{\theta }=(\hat{\mu },\hat{\phi },â)$ to develop, on the one hand, a test of goodness-of-fit to an NB distribution and, on the other hand, a two-sample PT-test for differences in means. This latter test is used for detecting differentially expressed genes. Without loss of generality, let *y*_*gk*_ be the number of counts for gene *g* in sample *k*, derived from pre-processing RNA-seq data. We assume that *y*_*gk*_ follows the PT distribution:

(9) $$y_{\text{gk}}∼\text{PT}(\mu_{g},\phi_{g},a_{g})\;.$$

In practice, we do not know the parameters *θ*_*g*_=*μ*_*g*_,*ϕ*_*g*_,*a*_*g*_, but we can estimate them from data by maximum likelihood when the sample size is sufficiently large so that it guarantees the desirable large sample properties of unbiasedness and minimum variance of the maximum likelihood estimate (MLE). In the Additional file 2: Supplementary Information we provide a simulation study in order to estimate the minimum number of samples per group that approximately meets this requirement (see Additional file 2: Figure S7).

We obtained the MLE $\hat{\theta }$ using a quasi-Newton method with constraints. We have implemented such a procedure using the optim function in R. In order to guarantee good convergence, we consider as initial parameters the moment estimates of *μ*_*g*_ and *ϕ*_*g*_, and *a*_*g*_=0. We choose this value for *a*_*g*_ because it corresponds to an NB model that is the natural cut-point of PT’s parameter space.

### Goodness-of-fit to a negative binomial distribution

In the framework of PT distributions we can formulate a test of the goodness of fit to an NB distribution by considering *H*_0_:*a*=0 versus *H*_*a*_:*a*≠0. Using a likelihood ratio test (LRT), the testing statistic is [34]

(10) $$T=\frac{\underset{\left(\hat{\mu },\hat{\phi },â\right)}{\max}\ell (\hat{\mu },\hat{\phi },\hat{a}|y_{0},\ldots ,y_{m})}{\underset{\left(\hat{\mu },\hat{\phi }\right)}{\max}\ell (\hat{\mu },\hat{\phi }|y_{0},\ldots ,y_{m})}\;,$$

where numerator and denominator correspond to the likelihood functions for the PT and NB models, respectively. Since the PT model has just one parameter more than the NB model, the quantity $2\log T∼\chi_{1}^{2}$ under the null hypothesis, as *n* grows large, and it can be used to decide whether count data follow a NB distribution by means of a Q-Q plot (see Additional file 2: Figure S2) or by calculating the corresponding *P*-value.

### Test to determine differentially expressed genes

For a given gene, let us assume that we observe *c*_1_,*c*_2_,…,*c*_*n*_ counts for *n* individuals and that we tabulate these counts into a contingency table with cells, *y*_0_,*y*_1_,…,*y*_*m*_ where *m*=max{*c*_1_,…,*c*_*n*_}. Therefore, *y*_*c*_ represents the number of observations with *c* counts. Then, the log-likelihood can be written as follows

(11) $$\log\ell (\hat{\theta }|y_{0},\ldots ,y_{m})=\overset{m}{\underset{i=0}{\sum }}y_{i}\;l_{i}(\hat{\theta })\;,$$

where $l_{i}(\hat{\theta })=\log[p_{i}(\hat{\theta })]$ and $p_{i}(\hat{\theta })$ denotes the mass probability at *i* with *i*=0,1,…,*m* and is computed using the recurrence given in equation (6). El-Shaarawi *et al.* (2011) [34] indicate that when regularity conditions hold, that is, when *θ* is an interior point of the parameter space *θ*, asymptotic normality of $\hat{\theta }$ can be assumed. Therefore, the negative inverse Hessian matrix of the log-likelihood at the MLE $\hat{\theta }$ corresponds to the estimated covariance matrix of $\hat{\theta }$. In particular, for the *μ* parameter we have that

(12) $$\text{Var}(\mu )=-E{\left[\frac{\partial^{2}}{\partial \mu^{2}}\log\ell (\hat{\theta }|y_{0},\ldots ,y_{m})\right]}^{-1}.$$

Consequently, if we are interested in comparing the mean counts for two sample groups, denoted by *μ*_*A*_ and *μ*_*B*_, a two-sample PT-test for the mean with null hypothesis $H_{0}:\frac{\mu_{A}}{\mu_{B}}=1$, which we perform in logarithmic scale as *H*_0_: log(*μ*_*A*_)= log(*μ*_*B*_), can be built by calculating the PT-statistic:

(13) $$T=\frac{{\hat{\mu }}_{A}-{\hat{\mu }}_{B}}{\sqrt{\text{Var}(\mu_{A})+\text{Var}(\mu_{B})}}\;,$$

The PT-statitic, *T*, follows a standard normal distribution under the null hypothesis. Therefore, the (1−*α*)*%* percentile of a *N*(0,1) distribution is used to determine whether the observed differences between the two groups are statistically significant or not by providing a corresponding *P*-value that can be later on corrected for multiple testing using, for instance, Benjamini-Hochberg’s FDR [35].

### Simulation of RNA-seq data

The results shown in Figure 6 recreating the null hypothesis of no DE with real RNA-seq data were performed by dividing the LCL data into two separate data sets of male and female samples. From each data set we bootstrapped 100 times two groups of 20 samples uniformly at random, thus obtaining on the one hand, group pairs of female samples and, on the other hand, group pairs of male samples. On each bootstrapped data set we performed the two-sample test for DE detection of every method between the groups of female versus female samples and male versus male samples. We also considered two versions of the data, one with the raw un-normalized counts and the other with the counts normalized with the cqn package [4]. In principle, there are no DE genes to be discovered from these comparisons, and therefore, under the null hypothesis of no DE, the *P*-value distribution for any given gene throughout the 100 bootstrapped data sets should be uniform.

The simulations shown in Figures 7, 8 and 9 contained synthetic RNA-seq data generated from a gamma-Poisson mixture model in a similar way to other published studies [8]. Under this model, we first draw dispersion parameters *ϕ*_*g*_ for every gene *g* at random from a gamma distribution Gamma(*k*=2,*θ*=0.7) and means according to three different fold-changes (1.5, 2 and 4) where half of the genes were up-regulated and the other half down-regulated. The *λ*_*gi*_ Poisson parameter for every gene *g* and sample *i* was drawn at random from a gamma distribution Gamma(*k*=*a*,*θ*=1/(*ϕ*−1)) with *a*=*fμ*_*gk*_/(*ϕ*−1) and *f*≈*N*(0,*σ*) corresponding to library factor which was either constant (*σ*=0) or variable (*σ*=0.5). Counts were simulated for each gene *g* from the resulting mixture gamma-Poisson distribution with parameters *λ*_*gi*_ for each sample *i*. Note that the resulting marginal distribution from the gamma-Poisson is a negative-binomial.

## Software availability

•**Project name:**tweeDEseq

•**Project home page: **http://www.bioconductor.org/packages/release/bioc/html/tweeDEseq.html

•**Operating system(s):** Platform independent

•**Programming language:**R and C

•**Other requirements:**R 3.0.0

•**Licence:** GNU GPL

•Any restrictions tu use by non-academics: no restrictions

## Competing interests

The authors declare that they have no competing interests.

## Authors’ contributions

JRG and PP conceived the idea of modelling RNA-seq count data using PT family of distributions. ME programmed the recursive algorithm to compute PT probability distribution, performed simulation studies, and created the R package jointly with JRG and RC. PP and JRG proposed the statistical test for detecting DE genes. DG preprocessed the RNA-seq data. RC, ME and JRG analysed the data and wrote the paper. The project was supervised by JRG. All authors read and approved the final manuscript.

## Supplementary Material

Additional file 1 **Scripts.** ZIP file (.zip) containing all scripts, in the form of Sweave vignettes, to reproduce the results shown in this paper, including one copy of the resulting PDF file. Please read first through the README file contained in this tar ball in order to understand how to run the scripts. Click here for file

Additional file 2 **Supplementary materials.** PDF file including supplementary figures and tables. Click here for file
