# Supplementary material for: A flexible count data model to fit the wide diversity of expression profiles arising from extensively replicated RNA-seq experiments
Source: BMC Bioinformatics. 2013 Aug 21;14:254. doi: 10.1186/1471-2105-14-254 (PMC3849762; doi:10.1186/1471-2105-14-254)
Supplement: Additional file 1 — Scripts. ZIP file (.zip) containing all scripts, in the form of Sweave vignettes, to reproduce the results shown in this paper, including one copy of the resulting PDF file. Please read first through the README file contained in this tar ball in order to understand how to run the scripts. [file 1471-2105-14-254-S1.zip › tweeDEseqSupportingAnalyses/tweeDEseqSupportingAnalyses.pdf]

Supporting analyses for the article "A flexible count data model to fit the wide diversity of expression profiles arising from extensively replicated RNA-seq experiments"

Mikel Esnaola[1] Pedro Puig[2], David Gonzalez[3] Robert Castelo[4,5,*]
robert.castelo@upf.edu

Juan Ramón González[1,2,5,6,*]
jrgonzalez@creal.cat

March 21, 2013

[1]Centre for Research in Environmental Epidemiology (CREAL), Barcelona, Spain

2 Department of Mathematics, Universitat Autònoma de Barcelona (UAB), Barcelona, Spain

3 Center for Genomic Regulation (CRG), Barcelona, Spain

4 Department of Experimental and Health Sciences, Research Program on Biomedical Informatics (GRIB), Universitat Pompeu Fabra, Barcelona, Spain

5 Hospital del Mar Research Institute (IMIM), Barcelona, Spain

6 CIBER Epidemiology and Public Health (CIBERESP), Spain

∗ Corresponding authors: Juan R Gonzalez (jrgonzalez@creal.cat) and R Castelo (robert.castelo@upf.edu)

Contents

1 Setup 2

2 Data import 3

3 Filtering 4

4 Normalization 4
4.1 Un-normalized data 5
4.2 Normalizing by the specific method proposed by each package 5
4.3 Normalizing by cqn 6
4.4 Comparison of the resulting normalized tables of counts 7
4.5 Session information for filtering and normalization 8

5 Goodness of fit 8
5.1 Testing Goodness-of-fit to a negative binomial distribution 9
5.2 Distribution of the shape parameter a 9
5.3 Breadth of expression for different count data distributions 10
5.4 Session information for goodness of fit 12

6 Differential expression 13
6.1 Testing for differential expression with tweeDEseq 13
6.2 Testing for differential expression with DESeq 14
6.3 Testing for differential expression with edgeR 18

7 Assessing differential expression calling accuracy **23**
7.1 Calling accuracy for tweeDEseq 24
7.2 Calling accuracy for DESeq 25
7.3 Calling accuracy for edgeR 29
7.4 Comparison of all DE calling accuracies 33
7.5 Session information for differential expression 38

8 Reproducibility in differential expression **38**
8.1 Testing count data for differential expression with tweeDEseq 39
8.2 Testing count data for differential expression with DESeq 39
8.3 Testing count data for differential expression with edgeR 40
8.4 Testing microarray data for differential expression with limma 41
8.5 Comparison of reproducibility of significance levels of differential expression 41
8.6 Comparing RNA-seq and microarray DE analyses 42
8.7 Session information for reproducibility in differential expression 45

1 Setup

This entire analysis is carried out in R using a variety of packages available at http://cran.r-project.org and at the http://www.bioconductor.org project. Among the latter bundle of packages, Biobase provides a function called `cache()` which we will employ all throughout the entire analysis in order to load pre-computed results when we do not want to calculate them again. For this purpose we define directories with the prefix `results` where pre-computed objects will be stored and a prefix name `cache_` for the files storing these objects, both stored in variables `cacheDir` and `cachePrefix`, respectively, as we will see below. In order to re-compute everything it suffices then to remove the directories with names under `cacheDir`. We set now the variable storing the prefix name.

```
> cachePrefix <- "cache_"
```

In a directory called `figures` the files from all figures will be stored and should be created if it does not exist.

```
> if (!file.exists("figures"))
    dir.create("figures")
```

We start by loading the necessary libraries, tweeDEseq for the package implementing the test for differential expression that employs the Poisson-Tweedie family of distributions, tweeDEseqCountData for the experimental and functional data employed in the analysis, DESeq for the DESeq method and edgeR for the edgeR method.

```
> library(Biobase)
> library(tweeDEseq)
> library(DESeq)
> library(edgeR)
> library(limma)
```

The results of this vignette are stored in two directories, one called `resultsPickrell1` and the other `resultsPickrell1huang`. If they do not exist, because we have removed them in order to re-compute everything again, we should create them

```
> if (!file.exists("resultsPickrell1"))
    dir.create("resultsPickrell1")
> if (!file.exists("resultsPickrell1huang"))
    dir.create("resultsPickrell1huang")
```

These two directory names will be later the ones pointed by the variable `cacheDir` to store and load cached results.

2 Data import

We load into the workspace the data corresponding to the initial table of raw counts of the RNA-seq experiment from (Pickrell et al., 2010) on lymphoblastoid cell lines derived from unrelated nigerian individuals and copy it into a variable called `countsRaw` which will be used in the rest of this section wherever is required the initial table of raw counts. These data are stored in the Bioconductor experimental data package tweeDEseqCountData:

```
> cacheDir <- "resultsPickrell1"
> data(pickrell1)
> pickrell1.eset
ExpressionSet (storageMode: lockedEnvironment)
assayData: 38415 features, 69 samples
  element names: exprs
protocolData: none
phenoData
  sampleNames: NA18486 NA18498 ... NA19257 (69 total)
  varLabels: num.tech.reps population study gender
  varMetadata: labelDescription
featureData
  featureNames: ENSG00000000003 ENSG00000000005 ... LRG_99 (52580
    total)
  fvarLabels: gene
  fvarMetadata: labelDescription
experimentData: use 'experimentData(object)'
Annotation:
> countsRaw <- exprs(pickrell1.eset)
> dim(countsRaw)
[1] 38415    69
> countsRaw[1:5, 1:5]
                NA18486 NA18498 NA18499 NA18501 NA18502
ENSG00000127720       6      32      14      35      14
ENSG00000242018      20      21      24      22      16
ENSG00000224440       0       0       0       0       0
ENSG00000214453       0       0       0       0       0
ENSG00000237787       0       0       1       0       0
```

We also need to load the vector of gender labels for each of the 69 samples and we also copy it into another variable called `genderCondition` which will be used later to define samples groups in the search for sex-specific expression changes. The order of this vector matches the sample order in the columns of the table of counts in `countsRaw`:

```
> genderCondition <- pickrell1.eset$gender
> length(genderCondition)
[1] 69
> head(genderCondition)
[1] male   male   female male   female male
Levels: female male
> table(genderCondition)
genderCondition
female   male
    40     29
```

At some point later on, we will need to use the total sequencing depth, or initial library sizes:

```
> initialLibSizes <- colSums(countsRaw)
```

3 Filtering

We start off with filtering out genes with very low expression levels using the function `filterCounts()` from the tweeDEseq package. Concretely, we only keep those genes with a minimum average of 0.1 counts per million throughout the samples:

```
> cache(countsFiltered <- filterCounts(countsRaw, mean.cpm.cutoff=0.1),
        dir=cacheDir, prefix=cachePrefix)
> dim(countsFiltered)
[1] 31226    69
```

4 Normalization

Normalizing the table of counts aims at removing sources of non-biological variation that might be biasing fold-changes and the overall observed variability. Here we will consider the following approaches to normalizing the initial table of counts:

1. Un-normalized data, we do not apply any normalization procedure.
2. Normalizing by the specific method proposed by each package.
3. Normalizing by cqn.

The latter two methods are independent from any DE analysis package and, differently to the normalization methods currently available in each of the DE analysis packages, they require gene length and gene G+C content information to normalize the counts. This information is contained in the `annotEnsembl63` *data.frame* that forms part of the tweeDEseqCountData experimental data package and proceed to load it here first:

```
> data(annotEnsembl63)
> dim(annotEnsembl63)
[1] 50451     8
> head(annotEnsembl63)
                Symbol Chr     Start       End EntrezID
ENSG00000252775     U7   5 133913821 133913880     <NA>
ENSG00000207459     U6   5 133970529 133970635     <NA>
ENSG00000252899     U7   5 133997420 133997479     <NA>
ENSG00000201298     U6   5 134036862 134036968     <NA>
ENSG00000222266     U6   5 134051173 134051272     <NA>
ENSG00000222924     U6   5 137405044 137405147     <NA>
                                                     Description Length
ENSG00000252775 U7 small nuclear RNA  [Source:RFAM;Acc:RF00066]     NA
ENSG00000207459  U6 spliceosomal RNA  [Source:RFAM;Acc:RF00026]     NA
ENSG00000252899 U7 small nuclear RNA  [Source:RFAM;Acc:RF00066]     NA
ENSG00000201298  U6 spliceosomal RNA  [Source:RFAM;Acc:RF00026]     NA
ENSG00000222266  U6 spliceosomal RNA  [Source:RFAM;Acc:RF00026]     NA
ENSG00000222924  U6 spliceosomal RNA  [Source:RFAM;Acc:RF00026]     NA
                GCcontent
ENSG00000252775        NA
ENSG00000207459        NA
ENSG00000252899        NA
ENSG00000201298        NA
ENSG00000222266        NA
ENSG00000222924        NA
```

4.1 Un-normalized data

Since here we do not apply any normalization procedure it is only necessary to put the previously filtered table of counts `countsFiltered` into the class of object that the corresponding method requires to perform the DE analysis. For instance, to use DESeq we should first create an object of the package built-in class *CountDataSet*:

```
> cdsUn <- newCountDataSet(countsFiltered, genderCondition)
> class(cdsUn)
[1] "CountDataSet"
attr(,"package")
[1] "DESeq"
> dim(cdsUn)
Features  Samples
   31226       69
```

and then set the size factors to the unity vector in order to force DESeq to work with the un-normalized data:

```
> sizeFactors(cdsUn) <- rep(1, ncol(countsFiltered))
```

In order to use edgeR we should create an object of the package built-in class *DGEList*:

```
> dUn <- DGEList(counts=countsFiltered, group=genderCondition,
                 lib.size=initialLibSizes)
> class(dUn)
[1] "DGEList"
attr(,"package")
[1] "edgeR"
> dim(dUn)
[1] 31226    69
```

By deafult, edgeR sets the normalization factors of this object to the unity vector, thus nothing else needs to be done in order to keep this data without normalization:

```
> dUn$samples$norm.factors
 [1] 1 1 1 1 1 1 1 1 1 1 1 1 1 1 1 1 1 1 1 1 1 1 1 1 1 1 1 1 1 1 1 1 1 1 1 1 1 1
[39] 1 1 1 1 1 1 1 1 1 1 1 1 1 1 1 1 1 1 1 1 1 1 1 1 1 1 1 1 1 1 1
```

In this case, where we do not normalize the counts all three different classes of objects, the tables of counts embedded in each of these objects should be identical:

```
> stopifnot(identical(as.matrix(countsFiltered), counts(cdsUn)))
> stopifnot(identical(as.matrix(countsFiltered), dUn$counts))
```

4.2 Normalizing by the specific method proposed by each package

The tweeDEseqpackage normalizes counts by relying on the corresponding functions from the edgeR package (comprising RNA composition adjustment by TMM (Robinson and Oshlack, 2010) and quantile-to-quantile count adjustment). This is available through one function (`normalizeCounts()`) that makes the appropriate calls to edgeR to normalize these data with this methodology. The call could be the following:

```
> cache(countsNormTweeDEseq <- normalizeCounts(countsFiltered, genderCondition),
        dir=cacheDir, prefix=cachePrefix)
```

In the case of the DESeq package we estimate the normalizing factors on the previously built unnormalized *CountDataSet* object:

```
> cache(cdsNorm <- estimateSizeFactors(cdsUn), dir=cacheDir, prefix=cachePrefix)
> sizeFactors(cdsNorm)
  NA18486   NA18498   NA18499   NA18501   NA18502   NA18504   NA18505   NA18507
0.6615410 1.4146759 0.7697261 0.9694701 1.1805085 0.9077265 1.1884069 1.3872131
  NA18508   NA18510   NA18511   NA18516   NA18517   NA18519   NA18520   NA18522
1.2228788 0.8116763 1.4603588 1.0447032 1.2534273 1.1609407 0.9910178 1.1525016
  NA18523   NA18852   NA18853   NA18855   NA18856   NA18858   NA18861   NA18862
0.5004175 1.2368743 0.7911536 0.9297654 0.7394884 1.1289520 0.6980175 1.0617676
  NA18870   NA18871   NA18909   NA18912   NA18913   NA18916   NA19093   NA19098
0.8773727 0.7546645 1.2489244 0.5016136 1.0675970 1.0962096 0.9674903 1.5384962
  NA19099   NA19101   NA19102   NA19108   NA19114   NA19116   NA19119   NA19127
1.4149887 1.3390745 0.4694445 1.5027936 0.8252121 1.3249745 0.4384142 1.0937449
  NA19128   NA19130   NA19131   NA19137   NA19138   NA19140   NA19143   NA19144
1.1559075 1.3372781 1.1398046 0.8939310 1.1932709 1.1849143 1.0858087 1.1265441
  NA19147   NA19152   NA19153   NA19159   NA19160   NA19171   NA19172   NA19190
0.9984960 1.3907193 1.1884742 0.7072425 1.2083385 0.8864393 0.9061773 0.9161241
  NA19192   NA19193   NA19200   NA19201   NA19203   NA19204   NA19209   NA19210
1.1092539 0.8462426 0.8122994 1.2701342 1.1413128 0.8735203 1.3025219 1.2151457
  NA19222   NA19225   NA19238   NA19239   NA19257
0.6766898 1.0258389 1.2305045 1.3584329 1.0118720
```

With edgeR we also estimate normalizing factors on the *DGEList* object:

```
> cache(dNorm <- calcNormFactors(dUn), dir=cacheDir, prefix=cachePrefix)
> dNorm$samples$norm.factors
 [1] 0.9397580 1.0867137 0.9692115 1.2069747 0.9021972 0.9987159 0.9873463
 [8] 1.0102826 0.9864692 0.9431865 0.9880796 1.0433657 1.0105542 0.9164147
[15] 0.9812765 1.1334225 1.0174560 0.9471144 1.0127462 0.9074963 1.1925314
[22] 0.9269652 1.3426597 1.0316326 1.0340928 0.9208077 0.9490620 0.9083929
[29] 0.8668493 1.1362832 1.0441677 1.1410596 1.1409502 1.1364551 0.8647733
[36] 1.0248545 0.6838139 1.1329146 1.1218164 0.9329042 0.8860575 1.1184704
[43] 1.0665230 1.1208620 0.9975130 0.9043300 1.0531951 1.0287382 0.8851289
[50] 0.9785634 0.9935689 0.9138904 1.0115569 1.1409834 0.9417925 0.9118735
[57] 1.0163536 0.7424894 0.9696044 0.9488864 0.9387199 1.0791755 0.8921170
[64] 0.9831734 1.3040543 0.8704627 1.1082502 1.1865042 0.9317279
```

4.3 Normalizing by cqn

Now we normalize using the package cqn. We can only normalize counts from genes for which we have length and G+C information in order to normalize adjusting for these two factors:

```
> suppressMessages(library(cqn))
> common <- intersect(rownames(countsFiltered),
                      rownames(annotEnsembl63[!is.na(annotEnsembl63$Length), ]))
> length(common)
[1] 27438
```

We can normalize the data using the function `cqn()` as follows:

```
> cache(cqnNorm <- cqn(countsFiltered[common, ],
                       lengths=as.numeric(annotEnsembl63[common, "Length"]),
                       x=annotEnsembl63[common, "GCcontent"],
                       sizeFactors=initialLibSizes, verbose=FALSE),
        dir=cacheDir, prefix=cachePrefix)
> class(cqnNorm)
```

```
[1] "cqn"
```

The cqn method calculates offset values to be employed directly in the statistical model of the corresponding DE detection method. However, one can obtain a normalized table of counts in the following way:

```
> log2rpm <- cqnNorm$y + cqnNorm$offset
> log2r <- sweep(cqnNorm$y + cqnNorm$offset, 2, log2(initialLibSizes/1e6), FUN="+")
> cache(countsNormCQN <- ceiling(2^log2r-0.5), dir=cacheDir, prefix=cachePrefix)
```

Again, we need to create a *CountDataSet* object for DESeq and set the normalization factors to the unity vector:

```
> cdsCQN <- newCountDataSet(countsNormCQN, genderCondition)
> sizeFactors(cdsCQN) <- rep(1, ncol(countsNormCQN))
```

In the case of edgeR we will use directly the offset values stored in the cqnNorm object.

4.4 Comparison of the resulting normalized tables of counts

In Figure 1 we find the MA-plots for the transformed tables of counts after normalizing by each of the previous for methods. We do not observe large expression-level dependent biases but many of the large fold-changes occurring at genes with low-expression in panels a and b, vanish when applying cqn (c).

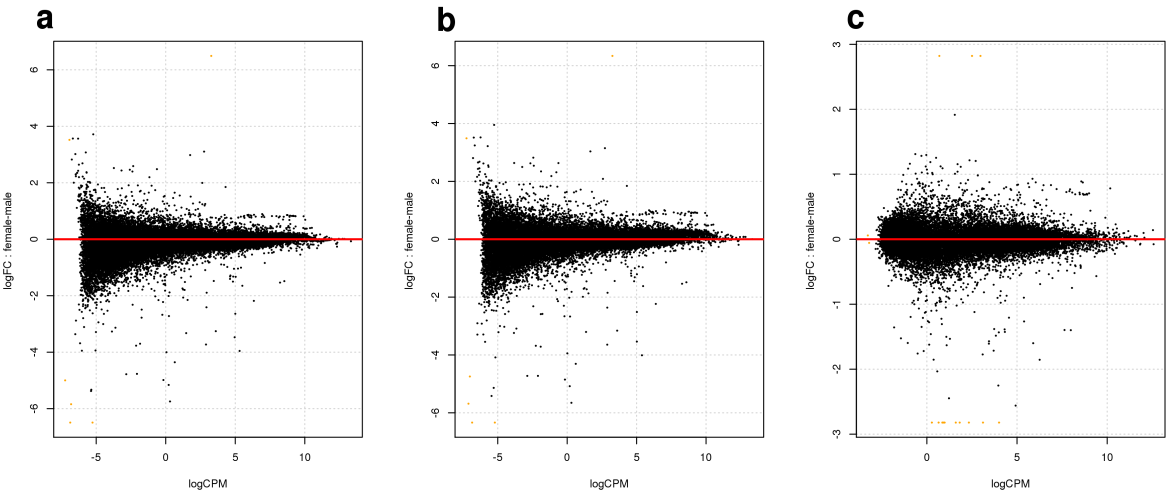

Figure 1. MA plots for the table of counts after applying no normalization (a), normalization by edgeR (b), and normalization by cqn (c). ote that the counts plotted here are obtained after transforming the initial raw ounts according to the corresponding normalization method.

In Figure 2 we have a multidimensional scaling (MDS) plot reflecting the "distance" between samples projected in two dimensions. This plot is produced with the `plotMDS()` function from the edgeR package over each of the normalized tables of counts. The `plotMDS()` function employs distance measure appropriate for RNA-seq data. Samples do not appear to group by male and female condition but they also scatter quite homogeneously through out the two dimensions without clear outliers.

```
> cache(mdsUn <- plotMDS.DGEList(dUn, col=c("blue", "red")[as.integer(genderCondition)],
                                labels=paste(toupper(substr(genderCondition, 1, 1)),
                                             1:dim(dUn)[2], sep=""), xlab="", ylab=""),
        dir=cacheDir, prefix=cachePrefix)
> cache(mdsNorm <- plotMDS.DGEList(dNorm, col=c("blue", "red")[as.integer(genderCondition)],
```

```
                                          labels=paste(toupper(substr(genderCondition, 1, 1)),
                                                       1:dim(dNorm)[2], sep=""), xlab="", ylab=""),
        dir=cacheDir, prefix=cachePrefix)
> cache(mdsCQN <- plotMDS.DGEList(DGEList(counts=countsNormCQN, group=genderCondition,
                                                 lib.size=initialLibSizes),
                                         col=c("blue", "red")[as.integer(genderCondition)],
                                         labels=paste(toupper(substr(genderCondition, 1, 1)),
                                                      1:dim(dUn)[2], sep=""), xlab="", ylab=""),
        dir=cacheDir, prefix=cachePrefix)
```

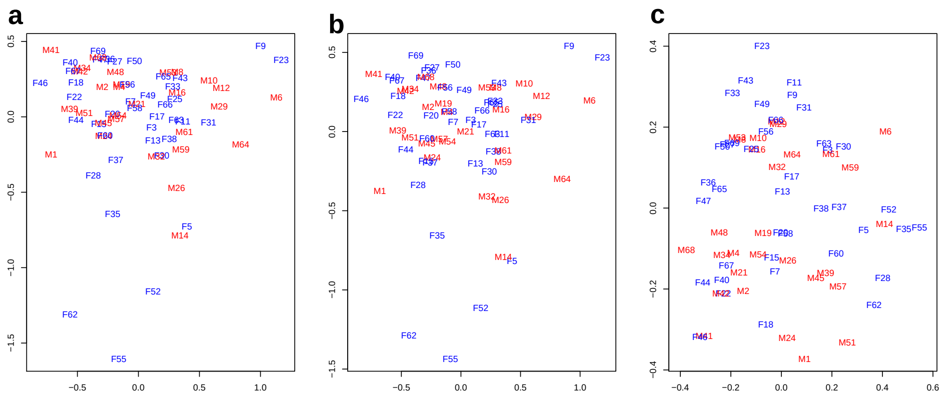

Figure 2. Multidimensional scaling (MDS) plot showing dissimilarities between samples projected on two dimensions. Blue and red indicate male and female samples, respectively.

4.5 Session information for filtering and normalization

```
> toLatex(sessionInfo())
```

- R version 2.15.1 (2012-06-22), `x86_64-unknown-linux-gnu`
- Locale: `LC_CTYPE=en_US.UTF-8`, `LC_NUMERIC=C`, `LC_TIME=en_US.UTF-8`, `LC_COLLATE=en_US.UTF-8`, `LC_MONETARY=en_US.UTF-8`, `LC_MESSAGES=en_US.UTF-8`, `LC_PAPER=C`, `LC_NAME=C`, `LC_ADDRESS=C`, `LC_TELEPHONE=C`, `LC_MEASUREMENT=en_US.UTF-8`, `LC_IDENTIFICATION=C`
- Base packages: base, datasets, graphics, grDevices, methods, splines, stats, utils
- Other packages: Biobase 2.18.0, BiocGenerics 0.4.0, cqn 1.4.0, DESeq 1.10.1, edgeR 3.0.8, lattice 0.20-10, limma 3.14.3, locfit 1.5-8, mclust 4.0, nor1mix 1.1-3, preprocessCore 1.20.0, quantreg 4.94, SparseM 0.96, tweeDEseq 1.4.0, tweeDEseqCountData 1.0.7
- Loaded via a namespace (and not attached): annotate 1.36.0, AnnotationDbi 1.20.3, DBI 0.2-5, genefilter 1.40.0, geneplotter 1.36.0, grid 2.15.1, IRanges 1.16.4, MASS 7.3-22, parallel 2.15.1, RColorBrewer 1.0-5, RSQLite 0.11.2, stats4 2.15.1, survival 2.37-2, tools 2.15.1, XML 3.95-0.1, xtable 1.7-0

5 Goodness of fit

In this section we examine the goodness of fit of every differently normalized table of counts to a negative binomial distribution. Afterwards, we examine the distribution of the shape parameter a for every gene

under the Poisson-Tweedie model. We employ the parallel package to speed up computations and use the variable `nCores` to set the number of threads that run in parallel. When `nCores` is set to 1 then all available cores are used.

```
> library(parallel)
> nCores <- 1
> nAvailableCores <- detectCores()
> if (nCores == 1)
    nCores <- nAvailableCores
> cacheDir <- "resultsPickrell1"
> stopifnot(file.exists(cacheDir)) ## sanity check
```

5.1 Testing Goodness-of-fit to a negative binomial distribution

Here we test the goodness of fit to the negative binomial distribution using the function `gof()` from the tweeDEseq package:

- Un-normalized data:

```
> cache(chi2gofUn <- gofTest(countsFiltered, a=0, mc.cores=nCores),
        dir=cacheDir, prefix=cachePrefix)
> names(chi2gofUn) <- rownames(countsFiltered)
```

- Normalized data with the edgeR methodology available through the tweeDEseq package itself:

```
> cache(chi2gofNorm <- gofTest(countsNormTweeDEseq, a=0, mc.cores=nCores),
                               dir=cacheDir, prefix=cachePrefix)
> names(chi2gofNorm) <- rownames(countsNormTweeDEseq)
```

- Normalized data with the cqn methodology:

```
> cache(chi2gofNormCQN <- gofTest(countsNormCQN, a=0, mc.cores=nCores),
                                  dir=cacheDir, prefix=cachePrefix)
> names(chi2gofNormCQN) <- rownames(countsNormCQN)
```

Using the function `qqchisq()` from the tweeDEseq package we can create χ^2 Q-Q plots displayed in Figure 3 to examine the goodness of fit of these data to a negative binomial distribution.

5.2 Distribution of the shape parameter a

Here we estimate the PT mean μ, dispersion D and shape parameter a for each table of counts pre-processed with a different normalization procedure.

```
> ff <- function(i, data)
   {
    x <- unlist(data[i,])
    ans <- try(mlePoissonTweedie(x), TRUE)
    if (inherits(ans, "try-error"))
     out <- NA
    else
     out <- c(ans$par, var=ans$se["mu"]^2*length(x))
    out
   }
```

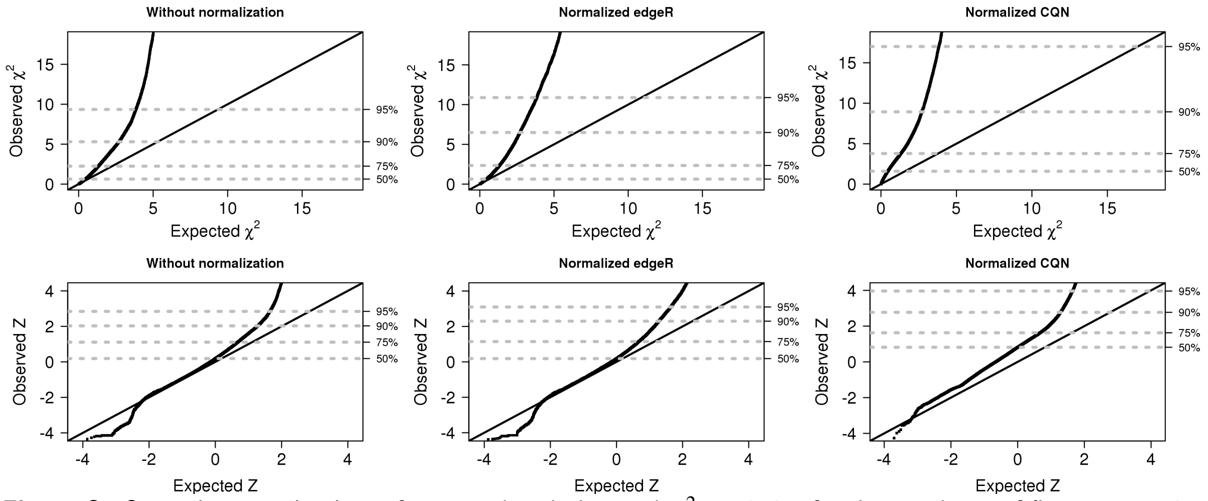

Figure 3. Quantile-quantile plots of expected and observed χ^2 statistics for the goodness of fit to a negative binomial distribution (top row) and transformed into normal z-scores (bottom row) to improve the display of lower quantiles.

- Un-normalized data:

```
> ## cache(aUn <- unlist(mclapply(1:nrow(countsFiltered), ff, data=countsFiltered, mc.cores=
> ##       dir=cacheDir, prefix=cachePrefix)
> ## names(aUn) <- rownames(countsFiltered)
> cache(aUn <- do.call("rbind", mclapply(1:nrow(countsFiltered), ff, data=countsFiltered, mc
        dir=cacheDir, prefix=cachePrefix)
> rownames(aUn) <- rownames(countsFiltered)
```

- Normalized data with the edgeR methodology available through the tweeDEseq package itself:

```
> ## cache(aNorm <- unlist(mclapply(1:nrow(countsNormTweeDEseq), ff, data=countsNormTweeDEse
> ##       dir=cacheDir, prefix=cachePrefix)
> ## names(aNorm) <- rownames(countsNormTweeDEseq)
> cache(aNorm <- do.call("rbind", mclapply(1:nrow(countsNormTweeDEseq), ff, data=countsNormT
        dir=cacheDir, prefix=cachePrefix)
> rownames(aNorm) <- rownames(countsNormTweeDEseq)
```

- Normalized data with the cqn methodology:

```
> ## cache(aNormCQN <- unlist(mclapply(1:nrow(countsNormCQN), ff, data=countsNormCQN, mc.cor
> ##       dir=cacheDir, prefix=cachePrefix)
> ## names(aNormCQN) <- rownames(countsNormCQN)
> cache(aNormCQN <- do.call("rbind", mclapply(1:nrow(countsNormCQN), ff, data=countsNormCQN,
        dir=cacheDir, prefix=cachePrefix)
> rownames(aNormCQN) <- rownames(countsNormCQN)
```

In Figure 4 we can see the distribution of a values as function of the mean expression.

5.3 Breadth of expression for different count data distributions

Using the Barcode (McCall et al., 2011) database we estimate the breadth of expression for every gene. The file containing the initial values of expression through human tissues was downloaded from `http://rafalab.jhsph.edu/barcode/catalogGPL570.csv`. These values were calculated from probeset of the Affymetrix HG-U133plus2 chip.

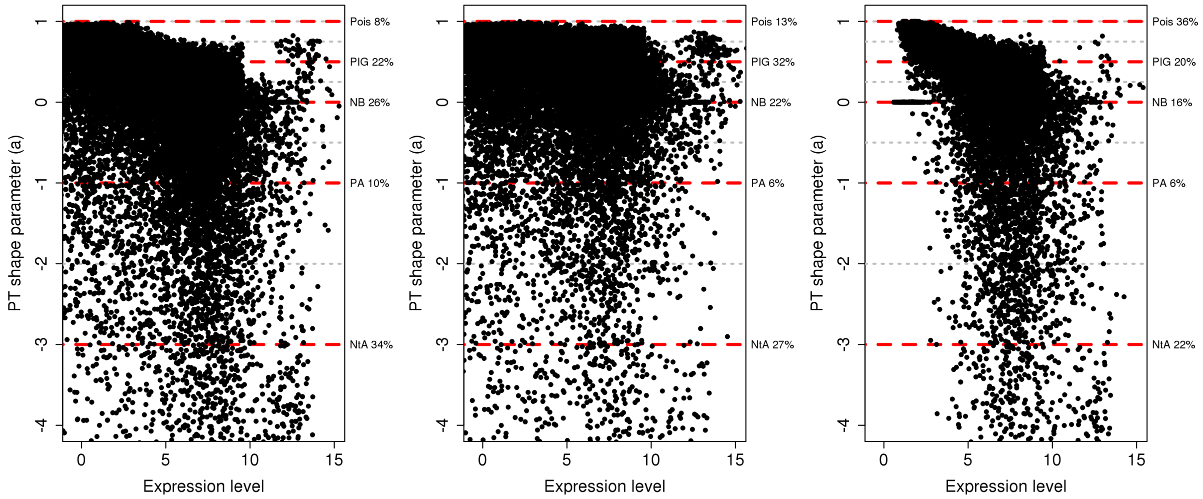

Figure 4. Distribution of the Poisson-Tweedie shape parameter as function of the mean expression level. Estimated Poisson-Tweedie shape parameter a as function of the mean expression level for each gene. Red dashed lines indicate the value of a corresponding to each specific distribution within the Poisson-Tweedie family, denoted by Pois (Poisson), PIG (Poisson-Inverse Gaussian), NB (negative binomial), PA (Pòlya-Aeppli) and NtA (Neyman type A). The right y-axis indicates the percentage of genes around specific a values bounded by dotted grey lines. Data from Pickrell et al. (2010) pre-processed by our own pipeline are shown without any normalization (a), normalized with edgeR (Robinson and Oshlack, 2010) (b) and cqn (Hansen et al., 2011) (c).

```
> library(tweeDEseqCountData)
> library(hgu133plus2.db)
> library(geneplotter)
> library(RColorBrewer)
> data(hkGenes)
> rafaGeneCatalog <- read.csv("catalogGPL570.csv", header=TRUE, stringsAsFactors=FALSE)
> dim(rafaGeneCatalog)
[1] 54613     6
```

Originally the Barcode catalog gives for each of the 54613 probesets of the HG-U133plus2 chip an estimate of expression breadth calculated as the proportion of tissue types in which a given probeset is expressed in more than half the samples, using publicly information from 18,656 publicly available microarray samples from 131 tissue types. The Barcode method also provides a measure of consistency of expression through samples of the same tissue, calculated as an entropy where a high value indicates an unreliable probeset. We discard here those probesets flagged by the Barcode data as unreliable because they have a high entropy value.

```
> rafaGeneCatalog <- rafaGeneCatalog[rafaGeneCatalog$HighEntropy == "No", ]
> dim(rafaGeneCatalog)
[1] 46220     6
```

Using the chip-level Bioconductor annotation package hgu133plus2.db we map the probesets of the Barcode catalog to Ensembl Gene Identifiers. When more than one probeset matches the same Ensembl ID, we take the maximum of its probeset values.

```
> ensID <- mget(rafaGeneCatalog$AffyID, hgu133plus2ENSEMBL, ifnotfound=NA)
> propExp <- data.frame(EnsID=unlist(ensID, use.names=FALSE),
                        Proportion=rep(rafaGeneCatalog$Proportion,
                                       times=sapply(ensID, length)))
> propExp <- split(propExp$Proportion, propExp$EnsID)
> length(propExp)
```

	HKG	nNByes	nNBno	Total	OR	Pvalue
**UnNorm**	Yes	31	546	577		
	No	187	26355	26542		
	Total	218	26901	27119	8.0	6.56e-17
	HKG	nNByes	nNBno	Total	OR	Pvalue
**edgeR**	Yes	19	558	577		
	No	36	27637	27673		
	Total	55	28195	28250	26.1	8.27e-19
	HKG	nNByes	nNBno	Total	OR	Pvalue
**cqn**	Yes	32	545	577		
	No	139	23265	23404		
	Total	171	23810	23981	9.8	1.49e-19

Table 1. Enrichment of non-NB genes among housekeeping genes through the data of Pickrell *et al.* (2010) processed with different normalization methods.

```
[1] 18583
> propExp <- sapply(propExp, max)
```

Using the χ^2 goodness-of-fit statistics to a negative binomial distribution calculated in the previous subsection and their corresponding P-values, we define one group of NB genes at $P > 0.2$ and another group of clear-cut non-NB genes at $P < 2^{-16}$. For each group we calculate the cumulative distribution of values of expression breadth. Figure 5 shows these distributions on the tables of counts obtained after applying each different normalization method. For comparison, the distribution of expression breadth values is also shown for a set of human housekeeping genes retrieved from the literature (Eisenberg and Levanon, 2003).

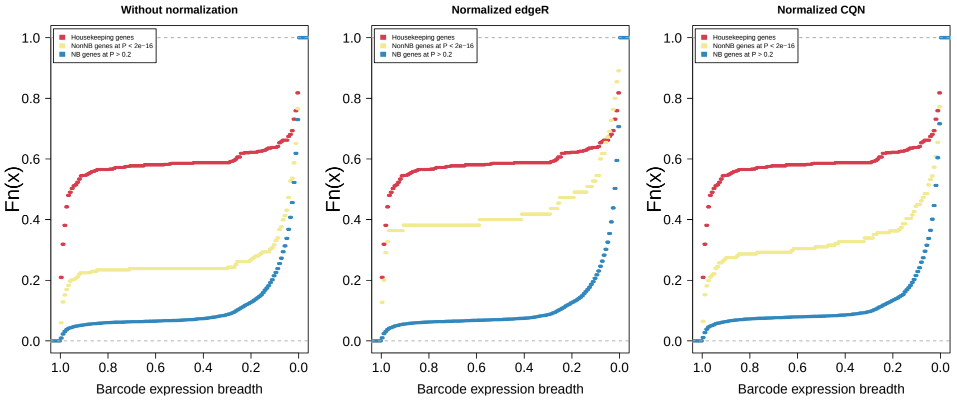

Figure 5. Breadth of expression estimated through the Barcode (McCall et al., 2011) database, for different subsets of genes defined by the P value of the goodness-of-fit test to a negative binomial distribution.

Figure 5 suggests that, in these expression data, there may be an enrichment of non-NB genes among the set of human housekeeping genes. We have assessed this hypothesis in each of the normalized data sets by means of a Fisher's exact test and the results shown in Table 1 confirms that this is true independently of how the data is normalized.

5.4 Session information for goodness of fit

```
> toLatex(sessionInfo())
```

- R version 2.15.1 (2012-06-22), `x86_64-unknown-linux-gnu`

- Locale: LC_CTYPE=en_US.UTF8, LC_NUMERIC=C, LC_TIME=en_US.UTF8, LC_COLLATE=en_US.UTF8, LC_MONETARY=en_US.UTF8, LC_MESSAGES=en_US.UTF8, LC_PAPER=C, LC_NAME=C, LC_ADDRESS=C, LC_TELEPHONE=C, LC_MEASUREMENT=en_US.UTF8, LC_IDENTIFICATION=C
- Base packages: base, datasets, graphics, grDevices, methods, parallel, splines, stats, utils
- Other packages: annotate 1.36.0, AnnotationDbi 1.20.3, Biobase 2.18.0, BiocGenerics 0.4.0, cqn 1.4.0, DBI 0.2-5, DESeq 1.10.1, edgeR 3.0.8, geneplotter 1.36.0, hgu133plus2.db 2.8.0, lattice 0.20-13, limma 3.14.4, locfit 1.5-8, mclust 4.0, nor1mix 1.1-3, org.Hs.eg.db 2.8.0, preprocessCore 1.20.0, quantreg 4.94, RColorBrewer 1.0-5, RSQLite 0.11.2, SparseM 0.96, tweeDEseq 1.4.1, tweeDEseqCountData 1.0.7, xtable 1.7-0
- Loaded via a namespace (and not attached): genefilter 1.40.0, grid 2.15.1, IRanges 1.16.5, MASS 7.3-23, stats4 2.15.1, survival 2.37-2, tools 2.15.1, XML 3.95-0.1

6 Differential expression

Throughout all the differential expression (DE) detection methods compared here, we call DE those genes with a minimum 1-fold change (i.e., no fold-change cutoff) at a maximum FDR of 10% and set these values in the following cutoff variables used in the rest of this document:

```
> fcCutoff <- 1
> fdrCutoff <- 0.1
> cacheDir <- "resultsPickrell1"
> stopifnot(file.exists(cacheDir)) ## sanity check
```

6.1 Testing for differential expression with tweeDEseq

In order to illustrate the accuracy of `tweeDEseq` for detecting DE genes in a extensively-replicated RNA-seq experiment we have compared the expression profiles between males and females from the population of 38415 unrelated Nigerian individuals (Pickrell et al., 2010).

The tweeDEseq package contains a function to test for differential expression between two different conditions using a score based test: the `tweeDE()` function. This function takes as input a matrix of counts with genes on the rows and samples on the columns and it can perform calculations in parallel if two or more cpu cores are available on the computer. In order to enable this feature one should have installed and load first the parallel package. One can also fine-tune the number of cores to be employed with the argument `mc.cores` which by default equals one implying that if parallel is loaded, all cores will be used. We set here one single variable to control this feature throughout all calls to the `tweeDE()` function:

```
> library(parallel)
> nCores <- 1
> nAvailableCores <- detectCores()
> if (nCores == 1)
    nCores <- nAvailableCores
```

We perform a DE analysis with the `tweeDE()` function for each different normalized input table of counts:

- Un-normalized data:

```
> cache(resTweeDEseqUn <- tweeDE(countsFiltered, group=genderCondition, pair=c("male", "fema
                                  mc.cores=nCores), dir=cacheDir, prefix=cachePrefix)
```

```
> dim(resTweeDEseqUn)
```

```
[1] 31226     7

> deGenesTweeDEseqUn <- print(resTweeDEseqUn, n=Inf, log2fc=log2(fcCutoff),
                              pval.adjust=fdrCutoff, sort.by="log2fc", print=FALSE)
> deGenesTweeDEseqUn <- deGenesTweeDEseqUn[!is.na(deGenesTweeDEseqUn$pval), ]
> dim(deGenesTweeDEseqUn)

[1] 59  7
```

- Normalized data with the edgeR methodology available through the tweeDEseq package itself:

```
> cache(resTweeDEseqNorm <- tweeDE(countsNormTweeDEseq, group=genderCondition,
                                   pair=c("male", "female"), mc.cores=nCores),
        dir=cacheDir, prefix=cachePrefix)

> dim(resTweeDEseqNorm)

[1] 31226     7

> deGenesTweeDEseqNorm <- print(resTweeDEseqNorm, n=Inf,
                                log2fc=log2(fcCutoff), pval.adjust=fdrCutoff,
                                sort.by="log2fc", print=FALSE)
> deGenesTweeDEseqNorm <- deGenesTweeDEseqNorm[!is.na(deGenesTweeDEseqNorm$pval), ]
> dim(deGenesTweeDEseqNorm)

[1] 80  7
```

- Normalized data with the cqn methodology:

```
> cache(resTweeDEseqNormCQN <- tweeDE(countsNormCQN, group=genderCondition,
                                      pair=c("male", "female"), mc.cores=nCores),
        dir=cacheDir, prefix=cachePrefix)

> dim(resTweeDEseqNormCQN)

[1] 27438     7

> deGenesTweeDEseqNormCQN <- print(resTweeDEseqNormCQN, n=Inf,
                                   log2fc=log2(fcCutoff),
                                   pval.adjust=fdrCutoff, sort.by="log2fc",
                                   print=FALSE)
> deGenesTweeDEseqNormCQN <- deGenesTweeDEseqNormCQN[!is.na(deGenesTweeDEseqNormCQN$pval), ]
> dim(deGenesTweeDEseqNormCQN)

[1] 55  7
```

6.2 Testing for differential expression with DESeq

In the case of DESeq, for each different normalization method we use the corresponding object created in the previous section to estimate tagwise dispersions, perform negative binomial testing and call DE genes at the previously defined significance cutoffs.

- Un-normalized data using arguments `method="per-condition"` and `sharingMode="gene-est-only"`:

```
> cache(cdsUnCgOnly <- estimateDispersions(cdsUn, method="per-condition", sharingMode="gene-
        dir=cacheDir, prefix=cachePrefix)
> cache(resDESeqUnCgOnly <- nbinomTest(cdsUnCgOnly, "male", "female"),
        dir=cacheDir, prefix=cachePrefix)
```

```
> dim(resDESeqUnCgOnly)

[1] 31226     8

> deGenesDESeqUnCgOnly <- resDESeqUnCgOnly[resDESeqUnCgOnly$padj < fdrCutoff &
                                          abs(resDESeqUnCgOnly$log2FoldChange) > log2
> deGenesDESeqUnCgOnly <- deGenesDESeqUnCgOnly[!is.na(deGenesDESeqUnCgOnly$pval), ]
> dim(deGenesDESeqUnCgOnly)

[1] 129   8
```

- Un-normalized data using arguments `method="per-condition"` and `sharingMode="maximum"`:

```
> cache(cdsUnCmax <- estimateDispersions(cdsUn, method="per-condition", sharingMode="maximum
        dir=cacheDir, prefix=cachePrefix)
> cache(resDESeqUnCmax <- nbinomTest(cdsUnCmax, "male", "female"),
        dir=cacheDir, prefix=cachePrefix)
```

```
> dim(resDESeqUnCmax)

[1] 31226     8

> deGenesDESeqUnCmax <- resDESeqUnCmax[resDESeqUnCmax$padj < fdrCutoff &
                                          abs(resDESeqUnCmax$log2FoldChange) > log2(f
> deGenesDESeqUnCmax <- deGenesDESeqUnCmax[!is.na(deGenesDESeqUnCmax$pval), ]
> dim(deGenesDESeqUnCmax)

[1] 85  8
```

- Un-normalized data using arguments `method="pooled"` and `sharingMode="gene-est-only"`:

```
> cache(cdsUnPgOnly <- estimateDispersions(cdsUn, method="pooled", sharingMode="gene-est-onl
        dir=cacheDir, prefix=cachePrefix)
> cache(resDESeqUnPgOnly <- nbinomTest(cdsUnPgOnly, "male", "female"),
        dir=cacheDir, prefix=cachePrefix)
```

```
> dim(resDESeqUnPgOnly)

[1] 31226     8

> deGenesDESeqUnPgOnly <- resDESeqUnPgOnly[resDESeqUnPgOnly$padj < fdrCutoff &
                                          abs(resDESeqUnPgOnly$log2FoldChange) > log2
> deGenesDESeqUnPgOnly <- deGenesDESeqUnPgOnly[!is.na(deGenesDESeqUnPgOnly$pval), ]
> dim(deGenesDESeqUnPgOnly)

[1] 61  8
```

- Un-normalized data using arguments `method="pooled"` and `sharingMode="maximum"`:

```
> cache(cdsUnPmax <- estimateDispersions(cdsUn, method="pooled", sharingMode="maximum"),
        dir=cacheDir, prefix=cachePrefix)
> cache(resDESeqUnPmax <- nbinomTest(cdsUnPmax, "male", "female"),
        dir=cacheDir, prefix=cachePrefix)
```

```
> dim(resDESeqUnPmax)
[1] 31226     8
> deGenesDESeqUnPmax <- resDESeqUnPmax[resDESeqUnPmax$padj < fdrCutoff &
                                            abs(resDESeqUnPmax$log2FoldChange) > log2(f
> deGenesDESeqUnPmax <- deGenesDESeqUnPmax[!is.na(deGenesDESeqUnPmax$pval), ]
> dim(deGenesDESeqUnPmax)
[1] 53  8
```

- Normalized data with DESeq's own methodology using arguments `method="per-condition"` and `sharingMode="gene-est-only"`:

```
> cache(cdsNormCgOnly <- estimateDispersions(cdsNorm, method="per-condition", sharingMode="g
        dir=cacheDir, prefix=cachePrefix)
> cache(resDESeqNormCgOnly <- nbinomTest(cdsNormCgOnly, "male", "female"),
        dir=cacheDir, prefix=cachePrefix)

> dim(resDESeqNormCgOnly)
[1] 31226     8
> deGenesDESeqNormCgOnly <- resDESeqNormCgOnly[resDESeqNormCgOnly$padj < fdrCutoff &
                                            abs(resDESeqNormCgOnly$log2FoldChange) > lo
> deGenesDESeqNormCgOnly <- deGenesDESeqNormCgOnly[!is.na(deGenesDESeqNormCgOnly$pval), ]
> dim(deGenesDESeqNormCgOnly)
[1] 178   8
```

- Normalized data with DESeq's own methodology using arguments `method="per-condition"` and `sharingMode="maximum"`:

```
> cache(cdsNormCmax <- estimateDispersions(cdsNorm, method="per-condition", sharingMode="max
        dir=cacheDir, prefix=cachePrefix)
> cache(resDESeqNormCmax <- nbinomTest(cdsNormCmax, "male", "female"),
        dir=cacheDir, prefix=cachePrefix)

> dim(resDESeqNormCmax)
[1] 31226     8
> deGenesDESeqNormCmax <- resDESeqNormCmax[resDESeqNormCmax$padj < fdrCutoff &
                                            abs(resDESeqNormCmax$log2FoldChange) > log2
> deGenesDESeqNormCmax <- deGenesDESeqNormCmax[!is.na(deGenesDESeqNormCmax$pval), ]
> dim(deGenesDESeqNormCmax)
[1] 103   8
```

- Normalized data with DESeq's own methodology using arguments `method="pooled"` and `sharingMode="gene-est-only"`:

```
> cache(cdsNormPgOnly <- estimateDispersions(cdsNorm, method="pooled", sharingMode="gene-est
        dir=cacheDir, prefix=cachePrefix)
> cache(resDESeqNormPgOnly <- nbinomTest(cdsNormPgOnly, "male", "female"),
        dir=cacheDir, prefix=cachePrefix)
```

```
> dim(resDESeqNormPgOnly)
```

```
[1] 31226     8
```

```
> deGenesDESeqNormPgOnly <- resDESeqNormPgOnly[resDESeqNormPgOnly$padj < fdrCutoff &
                                             abs(resDESeqNormPgOnly$log2FoldChange) > lo
> deGenesDESeqNormPgOnly <- deGenesDESeqNormPgOnly[!is.na(deGenesDESeqNormPgOnly$pval), ]
> dim(deGenesDESeqNormPgOnly)
```

```
[1] 70  8
```

- Normalized data with DESeq's own methodology using arguments `method="pooled"` and `sharingMode="maximum"`:

```
> cache(cdsNormPmax <- estimateDispersions(cdsNorm, method="pooled", sharingMode="maximum"),
        dir=cacheDir, prefix=cachePrefix)
> cache(resDESeqNormPmax <- nbinomTest(cdsNormPmax, "male", "female"),
        dir=cacheDir, prefix=cachePrefix)
```

```
> dim(resDESeqNormPmax)
```

```
[1] 31226     8
```

```
> deGenesDESeqNormPmax <- resDESeqNormPmax[resDESeqNormPmax$padj < fdrCutoff &
                                             abs(resDESeqNormPmax$log2FoldChange) > log2
> deGenesDESeqNormPmax <- deGenesDESeqNormPmax[!is.na(deGenesDESeqNormPmax$pval), ]
> dim(deGenesDESeqNormPmax)
```

```
[1] 53  8
```

- Normalized data with the cqn methodology using arguments `method="per-condition"` and `sharingMode="gene-est-only"`. Here we need to set `fitType="local"` to avoid getting an error:

```
> cache(cdsNormCQNCgOnly <- estimateDispersions(cdsCQN, method="per-condition", fitType="loc
        dir=cacheDir, prefix=cachePrefix)
> cache(resDESeqNormCQNCgOnly <- nbinomTest(cdsNormCQNCgOnly, "male", "female"),
        dir=cacheDir, prefix=cachePrefix)
```

```
> dim(resDESeqNormCQNCgOnly)
```

```
[1] 27438     8
```

```
> deGenesDESeqNormCQNCgOnly <- resDESeqNormCQNCgOnly[resDESeqNormCQNCgOnly$padj < fdrCutoff
                                             abs(resDESeqNormCQNCgOnly$log2FoldChange) >
> deGenesDESeqNormCQNCgOnly <- deGenesDESeqNormCQNCgOnly[!is.na(deGenesDESeqNormCQNCgOnly$pv
> dim(deGenesDESeqNormCQNCgOnly)
```

```
[1] 95  8
```

- Normalized data with the cqn methodology using arguments `method="per-condition"` and `sharingMode="maximum"`. Here we need to set `fitType="local"` to avoid getting an error:

```
> cache(cdsNormCQNCmax <- estimateDispersions(cdsCQN, method="per-condition", fitType="local
        dir=cacheDir, prefix=cachePrefix)
> cache(resDESeqNormCQNCmax <- nbinomTest(cdsNormCQNCmax, "male", "female"),
        dir=cacheDir, prefix=cachePrefix)
```

```
> dim(resDESeqNormCQNCmax)

[1] 27438     8

> deGenesDESeqNormCQNCmax <- resDESeqNormCQNCmax[resDESeqNormCQNCmax$padj < fdrCutoff &
                                                 abs(resDESeqNormCQNCmax$log2FoldChange) > l
> deGenesDESeqNormCQNCmax <- deGenesDESeqNormCQNCmax[!is.na(deGenesDESeqNormCQNCmax$pval), ]
> dim(deGenesDESeqNormCQNCmax)

[1] 63  8
```

- Normalized data with the cqn methodology using arguments `method="pooled"` and `sharingMode="gene-est-only"`. Here we need to set `fitType="local"` to avoid getting an error:

```
> cache(cdsNormCQNPgOnly <- estimateDispersions(cdsCQN, method="pooled", fitType="local", sh
        dir=cacheDir, prefix=cachePrefix)
> cache(resDESeqNormCQNPgOnly <- nbinomTest(cdsNormCQNPgOnly, "male", "female"),
        dir=cacheDir, prefix=cachePrefix)


> dim(resDESeqNormCQNPgOnly)

[1] 27438     8

> deGenesDESeqNormCQNPgOnly <- resDESeqNormCQNPgOnly[resDESeqNormCQNPgOnly$padj < fdrCutoff
                                                 abs(resDESeqNormCQNPgOnly$log2FoldChange) >
> deGenesDESeqNormCQNPgOnly <- deGenesDESeqNormCQNPgOnly[!is.na(deGenesDESeqNormCQNPgOnly$pv
> dim(deGenesDESeqNormCQNPgOnly)

[1] 53  8
```

- Normalized data with the cqn methodology using arguments `method="pooled"` and `sharingMode="maximum"`. Here we need to set `fitType="local"` to avoid getting an error:

```
> cache(cdsNormCQNPmax <- estimateDispersions(cdsCQN, method="pooled", fitType="local", shar
        dir=cacheDir, prefix=cachePrefix)
> cache(resDESeqNormCQNPmax <- nbinomTest(cdsNormCQNPmax, "male", "female"),
        dir=cacheDir, prefix=cachePrefix)


> dim(resDESeqNormCQNPmax)

[1] 27438     8

> deGenesDESeqNormCQNPmax <- resDESeqNormCQNPmax[resDESeqNormCQNPmax$padj < fdrCutoff &
                                                 abs(resDESeqNormCQNPmax$log2FoldChange) > l
> deGenesDESeqNormCQNPmax <- deGenesDESeqNormCQNPmax[!is.na(deGenesDESeqNormCQNPmax$pval), ]
> dim(deGenesDESeqNormCQNPmax)

[1] 46  8
```

6.3 Testing for differential expression with edgeR

In the case of edgeR, for each different normalization method we use the corresponding object created in the previous section to estimate tagwise dispersions, perform negative binomial testing and call DE genes at the previously defined significance cutoffs.

- Un-normalized data and common/trended/tagwise moderation regime with `prior.df=20` (default):

```
> design <- model.matrix(~ dUn$sample$group)
> cache(dUn.common <- estimateGLMCommonDisp(dUn, design), dir=cacheDir, prefix=cachePrefix)
> cache(dUn.trended <- estimateGLMTrendedDisp(dUn.common, design), dir=cacheDir, prefix=cach
> cache(dUn.tagwise <- estimateGLMTagwiseDisp(dUn.trended, design), dir=cacheDir, prefix=cac
> cache(dUn.fit <- glmFit(dUn.tagwise, design), dir=cacheDir, prefix=cachePrefix)
> cache(dUn.lrt <- glmLRT(dUn.fit, coef=2), dir=cacheDir, prefix=cachePrefix)
> cache(resEdgeRun <- topTags(dUn.lrt, n=Inf), dir=cacheDir, prefix=cachePrefix)
> dim(resEdgeRun)

[1] 31226     5

> deGenesEdgeRun <-
    resEdgeRun$table[rownames(resEdgeRun$table)[resEdgeRun$table$FDR < fdrCutoff &
                 abs(resEdgeRun$table$logFC) > log2(fcCutoff)], ]
> deGenesEdgeRun <- deGenesEdgeRun[!is.na(deGenesEdgeRun$PValue), ]
> dim(deGenesEdgeRun)

[1] 157   5
```

- Un-normalized data and common/trended/tagwise moderation regime with `prior.df=1`:

```
> cache(dUn.tagwise.df1 <- estimateGLMTagwiseDisp(dUn.trended, design, prior.df=1),
       dir=cacheDir, prefix=cachePrefix)
> cache(dUn.fit.df1 <- glmFit(dUn.tagwise.df1, design), dir=cacheDir, prefix=cachePrefix)
> cache(dUn.lrt.df1 <- glmLRT(dUn.fit.df1, coef=2), dir=cacheDir, prefix=cachePrefix)
> cache(resEdgeRunDf1 <- topTags(dUn.lrt.df1, n=Inf), dir=cacheDir, prefix=cachePrefix)
> dim(resEdgeRunDf1)

[1] 31226     5

> deGenesEdgeRunDf1 <-
    resEdgeRunDf1$table[rownames(resEdgeRunDf1$table)[resEdgeRunDf1$table$FDR < fdrCutoff &
                 abs(resEdgeRunDf1$table$logFC) > log2(fcCutoff)], ]
> deGenesEdgeRunDf1 <- deGenesEdgeRunDf1[!is.na(deGenesEdgeRunDf1$PValue), ]
> dim(deGenesEdgeRunDf1)

[1] 149   5
```

- Un-normalized data, common/trended/tagwise moderation regime with `prior.df=20` (default) and quasi-likelihood F-tests:

```
> cache(dUn.qlf <- glmQLFTest(dUn.fit, coef=2), dir=cacheDir, prefix=cachePrefix)
> cache(resEdgeRunQLF <- topTags(dUn.qlf, n=Inf), dir=cacheDir, prefix=cachePrefix)
> dim(resEdgeRunQLF)

[1] 31226     5

> deGenesEdgeRunQLF <-
    resEdgeRunQLF$table[rownames(resEdgeRunQLF$table)[resEdgeRunQLF$table$FDR < fdrCutoff &
                 abs(resEdgeRunQLF$table$logFC) > log2(fcCutoff)], ]
> deGenesEdgeRunQLF <- deGenesEdgeRunQLF[!is.na(deGenesEdgeRunQLF$PValue), ]
> dim(deGenesEdgeRunQLF)

[1] 132   5
```

- Un-normalized data, common/trended/tagwise moderation regime with `prior.df=1` and quasi-likelihood F-tests:

```
> cache(dUn.df1.qlf <- glmQLFTest(dUn.fit.df1, coef=2), dir=cacheDir, prefix=cachePrefix)
> cache(resEdgeRunQLFdf1 <- topTags(dUn.df1.qlf, n=Inf), dir=cacheDir, prefix=cachePrefix)
> dim(resEdgeRunQLFdf1)

[1] 31226     5

> deGenesEdgeRunQLFdf1 <-
    resEdgeRunQLFdf1$table[rownames(resEdgeRunQLFdf1$table)[resEdgeRunQLFdf1$table$FDR < fdr
                 abs(resEdgeRunQLFdf1$table$logFC) > log2(fcCutoff)], ]
> deGenesEdgeRunQLFdf1 <- deGenesEdgeRunQLFdf1[!is.na(deGenesEdgeRunQLFdf1$PValue), ]
> dim(deGenesEdgeRunQLFdf1)

[1] 111   5
```

- Normalized data with edgeR's own methodology and common/trended/tagwise moderation regime with `prior.df=20` (default):

```
> design <- model.matrix(~ dNorm$sample$group)
> cache(dNorm.common <- estimateGLMCommonDisp(dNorm, design), dir=cacheDir, prefix=cachePref
> cache(dNorm.trended <- estimateGLMTrendedDisp(dNorm.common, design), dir=cacheDir, prefix=
> cache(dNorm.tagwise <- estimateGLMTagwiseDisp(dNorm.trended, design), dir=cacheDir, prefix
> cache(dNorm.fit <- glmFit(dNorm.tagwise, design), dir=cacheDir, prefix=cachePrefix)
> cache(dNorm.lrt <- glmLRT(dNorm.fit, coef=2), dir=cacheDir, prefix=cachePrefix)
> cache(resEdgeRnorm <- topTags(dNorm.lrt, n=Inf), dir=cacheDir, prefix=cachePrefix)
> dim(resEdgeRnorm)

[1] 31226     5

> deGenesEdgeRnorm <-
    resEdgeRnorm$table[rownames(resEdgeRnorm$table)[resEdgeRnorm$table$FDR < fdrCutoff &
                 abs(resEdgeRnorm$table$logFC) > log2(fcCutoff)], ]
> deGenesEdgeRnorm <- deGenesEdgeRnorm[!is.na(deGenesEdgeRnorm$PValue), ]
> dim(deGenesEdgeRnorm)

[1] 154   5
```

- Normalized data with edgeR's own methodology and common/trended/tagwise moderation regime with `prior.n=1`:

```
> cache(dNorm.tagwise.df1 <- estimateGLMTagwiseDisp(dNorm.trended, design, prior.df=1),
        dir=cacheDir, prefix=cachePrefix)
> cache(dNorm.fit.df1 <- glmFit(dNorm.tagwise.df1, design), dir=cacheDir, prefix=cachePrefix
> cache(dNorm.lrt.df1 <- glmLRT(dNorm.fit.df1, coef=2), dir=cacheDir, prefix=cachePrefix)
> cache(resEdgeRnormDf1 <- topTags(dNorm.lrt.df1, n=Inf), dir=cacheDir, prefix=cachePrefix)
> dim(resEdgeRnormDf1)

[1] 31226     5

> deGenesEdgeRnormDf1 <-
    resEdgeRnormDf1$table[rownames(resEdgeRnormDf1$table)[resEdgeRnormDf1$table$FDR < fdrCut
                 abs(resEdgeRnormDf1$table$logFC) > log2(fcCutoff)], ]
> deGenesEdgeRnormDf1 <- deGenesEdgeRnormDf1[!is.na(deGenesEdgeRnormDf1$PValue), ]
> dim(deGenesEdgeRnormDf1)

[1] 139   5
```

- Normalized data with edgeR's own methodology and common/trended/tagwise moderation regime with `prior.df=20` (default) and quasi-likelihood F-tests:

```
> cache(dNorm.qlf <- glmQLFTest(dNorm.fit, coef=2), dir=cacheDir, prefix=cachePrefix)
> cache(resEdgeRnormQLF <- topTags(dNorm.qlf, n=Inf), dir=cacheDir, prefix=cachePrefix)
> dim(resEdgeRnormQLF)

[1] 31226     5

> deGenesEdgeRnormQLF <-
    resEdgeRnormQLF$table[rownames(resEdgeRnormQLF$table)[resEdgeRnormQLF$table$FDR < fdrCut
                          abs(resEdgeRnormQLF$table$logFC) > log2(fcCutoff)], ]
> deGenesEdgeRnormQLF <- deGenesEdgeRnormQLF[!is.na(deGenesEdgeRnormQLF$PValue), ]
> dim(deGenesEdgeRnormQLF)

[1] 140   5
```

- Normalized data with edgeR's own methodology and common/trended/tagwise moderation regime with `prior.df=1` and quasi-likelihood F-tests:

```
> cache(dNorm.df1.qlf <- glmQLFTest(dNorm.fit.df1, coef=2), dir=cacheDir, prefix=cachePrefix
> cache(resEdgeRnormQLFdf1 <- topTags(dNorm.df1.qlf, n=Inf), dir=cacheDir, prefix=cachePrefi
> dim(resEdgeRnormQLFdf1)

[1] 31226     5

> deGenesEdgeRnormQLFdf1 <-
    resEdgeRnormQLFdf1$table[rownames(resEdgeRnormQLFdf1$table)[resEdgeRnormQLFdf1$table$FDR
                             abs(resEdgeRnormQLFdf1$table$logFC) > log2(fcCutoff)], ]
> deGenesEdgeRnormQLFdf1 <- deGenesEdgeRnormQLFdf1[!is.na(deGenesEdgeRnormQLFdf1$PValue), ]
> dim(deGenesEdgeRnormQLFdf1)

[1] 118   5
```

- Normalized data with the cqn methodology and common/trended/tagwise moderation regime with `prior.n=20` (default):

```
> dUnCommonGenes <- dUn[common, ]
> design <- model.matrix(~ dUnCommonGenes$sample$group)
> cache(dCQN.common <- estimateGLMCommonDisp(dUnCommonGenes, design, offset=cqnNorm$offset),
        dir=cacheDir, prefix=cachePrefix)
> cache(dCQN.trended <- estimateGLMTrendedDisp(dCQN.common, design, offset=cqnNorm$offset),
        dir=cacheDir, prefix=cachePrefix)
> cache(dCQN.tagwise <- estimateGLMTagwiseDisp(dCQN.trended, design, offset=cqnNorm$offset),
        dir=cacheDir, prefix=cachePrefix)
> cache(dCQN.fit <- glmFit(dCQN.tagwise, design, offset=cqnNorm$offset),
        dir=cacheDir, prefix=cachePrefix)
> cache(dCQN.lrt <- glmLRT(dCQN.fit, coef=2), dir=cacheDir, prefix=cachePrefix)
> cache(resEdgeRnormCQN <- topTags(dCQN.lrt, n=Inf), dir=cacheDir, prefix=cachePrefix)
> dim(resEdgeRnormCQN)

[1] 27438     5

> deGenesEdgeRnormCQN <-
    resEdgeRnormCQN$table[rownames(resEdgeRnormCQN$table)[resEdgeRnormCQN$table$FDR < fdrCut
                 abs(resEdgeRnormCQN$table$logFC) > log2(fcCutoff)], ]
> deGenesEdgeRnormCQN <- deGenesEdgeRnormCQN[!is.na(deGenesEdgeRnormCQN$PValue), ]
> dim(deGenesEdgeRnormCQN)

[1] 85  5
```

- Normalized data with the cqn methodology and common/trended/tagwise moderation regime with prior.n=1:

```
> cache(dCQN.tagwise.df1 <- estimateGLMTagwiseDisp(dCQN.trended, design, prior.df=1, offset=
        dir=cacheDir, prefix=cachePrefix)
> cache(dCQN.fit.df1 <- glmFit(dCQN.tagwise.df1, design, offset=cqnNorm$offset),
        dir=cacheDir, prefix=cachePrefix)
> cache(dCQN.lrt.df1 <- glmLRT(dCQN.fit.df1, coef=2), dir=cacheDir, prefix=cachePrefix)
> cache(resEdgeRnormCQNdf1 <- topTags(dCQN.lrt.df1, n=Inf), dir=cacheDir, prefix=cachePrefix
> dim(resEdgeRnormCQNdf1)

[1] 27438     5

> deGenesEdgeRnormCQNdf1 <-
    resEdgeRnormCQNdf1$table[rownames(resEdgeRnormCQNdf1$table)[resEdgeRnormCQNdf1$table$FDR
                             abs(resEdgeRnormCQNdf1$table$logFC) > log2(fcCutoff)], ]
> deGenesEdgeRnormCQNdf1 <- deGenesEdgeRnormCQNdf1[!is.na(deGenesEdgeRnormCQNdf1$PValue), ]
> dim(deGenesEdgeRnormCQNdf1)

[1] 72  5
```

- Normalized data with the cqn methodology, common/trended/tagwise moderation regime with prior.df=20 (default) and quasi-likelihood F-tests:

```
> cache(dCQN.qlf <- glmQLFTest(dCQN.fit, coef=2), dir=cacheDir, prefix=cachePrefix)
> cache(resEdgeRnormCQNqlf <- topTags(dCQN.qlf, n=Inf), dir=cacheDir, prefix=cachePrefix)
> dim(resEdgeRnormCQNqlf)

[1] 27438     5

> deGenesEdgeRnormCQNqlf <-
    resEdgeRnormCQNqlf$table[rownames(resEdgeRnormCQNqlf$table)[resEdgeRnormCQNqlf$table$FDR
                 abs(resEdgeRnormCQNqlf$table$logFC) > log2(fcCutoff)], ]
> deGenesEdgeRnormCQNqlf <- deGenesEdgeRnormCQNqlf[!is.na(deGenesEdgeRnormCQNqlf$PValue), ]
> dim(deGenesEdgeRnormCQNqlf)

[1] 74  5
```

- Normalized data with the cqn methodology, common/trended/tagwise moderation regime with prior.df=20 (default) and quasi-likelihood F-tests:

```
> cache(dCQN.df1.qlf <- glmQLFTest(dCQN.fit.df1, coef=2), dir=cacheDir, prefix=cachePrefix)
> cache(resEdgeRnormCQNqlfDf1 <- topTags(dCQN.df1.qlf, n=Inf), dir=cacheDir, prefix=cachePre
> dim(resEdgeRnormCQNqlfDf1)

[1] 27438     5

> deGenesEdgeRnormCQNqlfDf1 <-
    resEdgeRnormCQNqlfDf1$table[rownames(resEdgeRnormCQNqlfDf1$table)[resEdgeRnormCQNqlfDf1$
                 abs(resEdgeRnormCQNqlfDf1$table$logFC) > log2(fcCutoff)], ]
> deGenesEdgeRnormCQNqlfDf1 <- deGenesEdgeRnormCQNqlfDf1[!is.na(deGenesEdgeRnormCQNqlfDf1$PV
> dim(deGenesEdgeRnormCQNqlfDf1)

[1] 67  5
```

7 Assessing differential expression calling accuracy

In order to try to assess the accuracy of all of the previous differential expression analysis methods we will compare each subset of DE genes to a list of genes with documented sex-specific expression by means of a Fisher's exact test. The list of genes with documented sex-specific expression was built by first selecting genes in chromosome X that escape X-inactivation (Carrel and HF, 2005), denoted here by XiE genes, and genes in the male-specific region of the Y chrosomome (Skaletsky et al., 2003) denoted by MSY genes. This list forms part of the the experimental data package tweeDEseqCountData and should be retrieved from the package in the following way:

```
> data(genderGenes)
> length(XiEgenes)
[1] 63
> length(msYgenes)
[1] 32
```

which loads two vector objects, `XiEgenes` and `msYgenes`, of Ensembl Gene IDs containing the two separated lists of XiE and MSY genes, respectively. For the purpose of evaluating the accuracy of the predictions we will put these two types of genes with sex-specific expression into a single vector:

```
> genderGenes <- c(msYgenes, XiEgenes)
> length(genderGenes)
[1] 95
```

against which the accuracy will be evaluated in terms of the harmonic mean of precision and recall, also known as the F-measure calculated as follows:

$$F = 2 \times \frac{\text{precision} \cdot \text{recall}}{\text{precision} + \text{recall}}, \tag{1}$$

and implemented in the following function:

```
> performance <- function(predictions, labels, predName="DE", labName="SEX", beta=1)
   {
     tt <- table(relevel(factor(labels, labels=c("No", "Yes")), 2),
                 relevel(factor(predictions, labels=c("No", "Yes")), 2),
                 dnn=list(labName, predName))
     tp <- tt[1, 1] ## true positive predictions
     fp <- tt[2, 1] ## false positive predictions
     fn <- tt[1, 2] ## false negative predictions

     p <- tp / (tp + fp)  ## precision
     r <- tp / (tp + fn)  ## recall

     F <- ((1+beta^2)*p*r) / (beta^2*p + r)

     ans <- list(table=tt, p=p, r=r, F=F)
     class(ans) <- "performance"
     ans
   }
> print.performance <- function(x, digits=2)
   {
     print(x$table)
     cat ("\n")
     cat("Precision:", round(x$p, digits=digits), "\n")
     cat("Recall:", round(x$r, digits=digits), "\n")
     cat("F-measure:", round(x$F, digits=digits), "\n")
   }
```

where the latter one is just made for displaying purposes.

For each combination of normalization and prediction method, we store the corresponding contingency table of predictions and correct labels and the value of the F-measure summarizing the accuracy of the predictions, in the following nested list data structure which will be filled out with the function defined below.

```
> cacc <- vector(mode="list", length=3)
> names(cacc) <- c("UnNorm", "OwnNorm", "cqn")
> cacc <- lapply(cacc, function(x) {
                        l <- vector(mode="list", length=9)
                        names(l) <- c("tweeDEseq", "DESeqCg0", "DESeqCmax", "DESeqPg0", "DESeqPr
                                      "edgeRdf20", "edgeRdf1", "edgeRqlfDf20", "edgeRqlfDf1")
                        l <- lapply(l, function(x)
                                        data.frame(SexSp=c("Yes", "No", "Total"),
                                                   DEyes=rep(NA, 3),
                                                   DEno=rep(NA, 3),
                                                   Total=rep(NA, 3),
                                                   Perf.=rep(NA, 3),
                                                   stringsAsFactors=FALSE))
                        l
                      })
> fillCallingAcc <- function(cacc, normMet, deMet, perf) {
    stopifnot(class(perf) == "performance")
    cacc[[normMet]][[deMet]][1:2, 2:3] <- perf$table
    cacc[[normMet]][[deMet]][1:2, 4] <- margin.table(perf$table, 1)
    cacc[[normMet]][[deMet]][3, 2:3] <- margin.table(perf$table, 2)
    cacc[[normMet]][[deMet]][3, 4] <- margin.table(perf$table)
    cacc[[normMet]][[deMet]][1, 5] <- sprintf("P=%.2f", perf$p)
    cacc[[normMet]][[deMet]][2, 5] <- sprintf("R=%.2f", perf$r)
    cacc[[normMet]][[deMet]][3, 5] <- sprintf("F=%.2f", perf$F)
    cacc
  }
```

7.1 Calling accuracy for tweeDEseq

- Un-normalized data:

```
> truelabels <- rownames(resTweeDEseqUn) %in% genderGenes
> predictions <- resTweeDEseqUn$pval.adjust < fdrCutoff &
                 abs(resTweeDEseqUn$log2fc) > log2(fcCutoff)
> perf <- performance(predictions, truelabels)
> perf

     DE
SEX     Yes    No
  Yes    16    63
  No     43 31104

Precision: 0.27
Recall: 0.2
F-measure: 0.23

> cacc <- fillCallingAcc(cacc, "UnNorm", "tweeDEseq", perf)
```

- Normalized data with the edgeR methodology available through tweeDEseq:

```
> truelabels <- rownames(resTweeDEseqNorm) %in% genderGenes
> predictions <- resTweeDEseqNorm$pval.adjust < fdrCutoff &
                 abs(resTweeDEseqNorm$log2fc) > log2(fcCutoff)
> perf <- performance(predictions, truelabels)
> perf
```

```
     DE
SEX     Yes    No
  Yes    24    55
  No     56 31091

Precision: 0.3
Recall: 0.3
F-measure: 0.3
```

```
> cacc <- fillCallingAcc(cacc, "OwnNorm", "tweeDEseq", perf)
```

- Normalized data with the `cqn` methodology:

```
> truelabels <- rownames(resTweeDEseqNormCQN) %in% genderGenes
> predictions <- resTweeDEseqNormCQN$pval.adjust < fdrCutoff &
                 abs(resTweeDEseqNormCQN$log2fc) > log2(fcCutoff)
> perf <- performance(predictions, truelabels)
> perf
```

```
     DE
SEX     Yes    No
  Yes    20    59
  No     35 27324

Precision: 0.36
Recall: 0.25
F-measure: 0.3
```

```
> cacc <- fillCallingAcc(cacc, "cqn", "tweeDEseq", perf)
```

7.2 Calling accuracy for DESeq

- Un-normalized data and arguments `method="per-condition"` and `sharingMode="gene-est-only"`:

```
> truelabels <- resDESeqUnCgOnly$id %in% genderGenes
> predictions <- resDESeqUnCgOnly$padj < fdrCutoff &
                 abs(resDESeqUnCgOnly$log2FoldChange) > log2(fcCutoff)
> perf <- performance(predictions, truelabels)
> perf
```

```
     DE
SEX     Yes    No
  Yes    18    61
  No    111 31036

Precision: 0.14
Recall: 0.23
F-measure: 0.17
```

```
> cacc <- fillCallingAcc(cacc, "UnNorm", "DESeqCgO", perf)
```

- Un-normalized data and arguments `method="per-condition"` and `sharingMode="maximum"`:

```
> truelabels <- resDESeqUnCmax$id %in% genderGenes
> predictions <- resDESeqUnCmax$padj < fdrCutoff &
                 abs(resDESeqUnCmax$log2FoldChange) > log2(fcCutoff)
> perf <- performance(predictions, truelabels)
> perf

     DE
SEX     Yes    No
  Yes    16    63
  No     69 31078

Precision: 0.19
Recall: 0.2
F-measure: 0.2

> cacc <- fillCallingAcc(cacc, "UnNorm", "DESeqCmax", perf)
```

- Un-normalized data and arguments `method="pooled"` and `sharingMode="gene-est-only"`:

```
> truelabels <- resDESeqUnPgOnly$id %in% genderGenes
> predictions <- resDESeqUnPgOnly$padj < fdrCutoff &
                 abs(resDESeqUnPgOnly$log2FoldChange) > log2(fcCutoff)
> perf <- performance(predictions, truelabels)
> perf

     DE
SEX     Yes    No
  Yes    16    63
  No     45 31102

Precision: 0.26
Recall: 0.2
F-measure: 0.23

> cacc <- fillCallingAcc(cacc, "UnNorm", "DESeqPgO", perf)
```

- Un-normalized data and arguments `method="pooled"` and `sharingMode="maximum"`:

```
> truelabels <- resDESeqUnPmax$id %in% genderGenes
> predictions <- resDESeqUnPmax$padj < fdrCutoff &
                 abs(resDESeqUnPmax$log2FoldChange) > log2(fcCutoff)
> perf <- performance(predictions, truelabels)
> perf

     DE
SEX     Yes    No
  Yes    13    66
  No     40 31107

Precision: 0.25
Recall: 0.16
F-measure: 0.2

> cacc <- fillCallingAcc(cacc, "UnNorm", "DESeqPmax", perf)
```

- Normalized data with the DESeq methodology and arguments `method="per-condition"` and `sharingMode="gene-est-only"`:

```
> truelabels <- resDESeqNormCgOnly$id %in% genderGenes
> predictions <- resDESeqNormCgOnly$padj < fdrCutoff &
                 abs(resDESeqNormCgOnly$log2FoldChange) > log2(fcCutoff)
> perf <- performance(predictions, truelabels)
> perf

     DE
SEX     Yes    No
  Yes    25    54
  No    153 30994

Precision: 0.14
Recall: 0.32
F-measure: 0.19

> cacc <- fillCallingAcc(cacc, "OwnNorm", "DESeqCgO", perf)
```

- Normalized data with the DESeq methodology and arguments `method="per-condition"` and `sharingMode="maximum"`:

```
> truelabels <- resDESeqNormCmax$id %in% genderGenes
> predictions <- resDESeqNormCmax$padj < fdrCutoff &
                 abs(resDESeqNormCmax$log2FoldChange) > log2(fcCutoff)
> perf <- performance(predictions, truelabels)
> perf

     DE
SEX     Yes    No
  Yes    18    61
  No     85 31062

Precision: 0.17
Recall: 0.23
F-measure: 0.2

> cacc <- fillCallingAcc(cacc, "OwnNorm", "DESeqCmax", perf)
```

- Normalized data with the DESeq methodology and arguments `method="pooled"` and `sharingMode="gene-est-only"`:

```
> truelabels <- resDESeqNormPgOnly$id %in% genderGenes
> predictions <- resDESeqNormPgOnly$padj < fdrCutoff &
                 abs(resDESeqNormPgOnly$log2FoldChange) > log2(fcCutoff)
> perf <- performance(predictions, truelabels)
> perf

     DE
SEX     Yes    No
  Yes    20    59
  No     50 31097

Precision: 0.29
Recall: 0.25
F-measure: 0.27
```

```
> cacc <- fillCallingAcc(cacc, "OwnNorm", "DESeqPgO", perf)
```

- Normalized data with the DESeq methodology and arguments method="pooled" and sharingMode="maximum":

```
> truelabels <- resDESeqNormPmax$id %in% genderGenes
> predictions <- resDESeqNormPmax$padj < fdrCutoff &
                 abs(resDESeqNormPmax$log2FoldChange) > log2(fcCutoff)
> perf <- performance(predictions, truelabels)
> perf

     DE
SEX     Yes    No
  Yes    14    65
  No     39 31108

Precision: 0.26
Recall: 0.18
F-measure: 0.21

> cacc <- fillCallingAcc(cacc, "OwnNorm", "DESeqPmax", perf)
```

- Normalized data with the cqn methodology and arguments method="per-condition" and sharingMode="gene-est-only":

```
> truelabels <- resDESeqNormCQNCgOnly$id %in% genderGenes
> predictions <- resDESeqNormCQNCgOnly$padj < fdrCutoff &
                 abs(resDESeqNormCQNCgOnly$log2FoldChange) > log2(fcCutoff)
> perf <- performance(predictions, truelabels)
> perf

     DE
SEX     Yes    No
  Yes    21    58
  No     74 27285

Precision: 0.22
Recall: 0.27
F-measure: 0.24

> cacc <- fillCallingAcc(cacc, "cqn", "DESeqCgO", perf)
```

- Normalized data with the cqn methodology and arguments method="per-condition" and sharingMode="maximum":

```
> truelabels <- resDESeqNormCQNCmax$id %in% genderGenes
> predictions <- resDESeqNormCQNCmax$padj < fdrCutoff &
                 abs(resDESeqNormCQNCmax$log2FoldChange) > log2(fcCutoff)
> perf <- performance(predictions, truelabels)
> perf

     DE
SEX     Yes    No
  Yes    16    63
  No     47 27312

Precision: 0.25
Recall: 0.2
F-measure: 0.23
```

```
> cacc <- fillCallingAcc(cacc, "cqn", "DESeqCmax", perf)
```

- Normalized data with the cqn methodology and arguments `method="pooled"` and `sharingMode="gene-est-only"`:

```
> truelabels <- resDESeqNormCQNPgOnly$id %in% genderGenes
> predictions <- resDESeqNormCQNPgOnly$padj < fdrCutoff &
                 abs(resDESeqNormCQNPgOnly$log2FoldChange) > log2(fcCutoff)
> perf <- performance(predictions, truelabels)
> perf

     DE
SEX     Yes    No
  Yes    19    60
  No     34 27325

Precision: 0.36
Recall: 0.24
F-measure: 0.29

> cacc <- fillCallingAcc(cacc, "cqn", "DESeqPgO", perf)
```

- Normalized data with the cqn methodology and arguments `method="pooled"` and `sharingMode="maximum"`:

```
> truelabels <- resDESeqNormCQNPmax$id %in% genderGenes
> predictions <- resDESeqNormCQNPmax$padj < fdrCutoff &
                 abs(resDESeqNormCQNPmax$log2FoldChange) > log2(fcCutoff)
> perf <- performance(predictions, truelabels)
> perf

     DE
SEX     Yes    No
  Yes    16    63
  No     30 27329

Precision: 0.35
Recall: 0.2
F-measure: 0.26

> cacc <- fillCallingAcc(cacc, "cqn", "DESeqPmax", perf)
```

7.3 Calling accuracy for edgeR

- Un-normalized data and common/trended/tagwise moderation regime with `prior.df=20` (default):

```
> truelabels <- rownames(resEdgeRun$table) %in% genderGenes
> predictions <- resEdgeRun$table$FDR < fdrCutoff &
                 abs(resEdgeRun$table$logFC) > log2(fcCutoff)
> perf <- performance(predictions, truelabels)
> perf

     DE
SEX     Yes    No
  Yes    20    59
  No    137 31010
```

```
Precision: 0.13
Recall: 0.25
F-measure: 0.17
```

```
> cacc <- fillCallingAcc(cacc, "UnNorm", "edgeRdf20", perf)
```

- Un-normalized data and common/trended/tagwise moderation regime with `prior.df=1`:

```
> truelabels <- rownames(resEdgeRunDf1$table) %in% genderGenes
> predictions <- resEdgeRunDf1$table$FDR < fdrCutoff &
                 abs(resEdgeRunDf1$table$logFC) > log2(fcCutoff)
> perf <- performance(predictions, truelabels)
> perf
```

```
     DE
SEX     Yes    No
  Yes    21    58
  No    128 31019

Precision: 0.14
Recall: 0.27
F-measure: 0.18
```

```
> cacc <- fillCallingAcc(cacc, "UnNorm", "edgeRdf1", perf)
```

- Un-normalized data, common/trended/tagwise moderation regime with `prior.df=20` (default) and quasi-likelihood F-tests:

```
> truelabels <- rownames(resEdgeRunQLF$table) %in% genderGenes
> predictions <- resEdgeRunQLF$table$FDR < fdrCutoff &
                 abs(resEdgeRunQLF$table$logFC) > log2(fcCutoff)
> perf <- performance(predictions, truelabels)
> perf
```

```
     DE
SEX     Yes    No
  Yes    19    60
  No    113 31034

Precision: 0.14
Recall: 0.24
F-measure: 0.18
```

```
> cacc <- fillCallingAcc(cacc, "UnNorm", "edgeRqlfDf20", perf)
```

- Un-normalized data, common/trended/tagwise moderation regime with `prior.df=1` and quasi-likelihood F-tests:

```
> truelabels <- rownames(resEdgeRunQLFdf1$table) %in% genderGenes
> predictions <- resEdgeRunQLFdf1$table$FDR < fdrCutoff &
                 abs(resEdgeRunQLFdf1$table$logFC) > log2(fcCutoff)
> perf <- performance(predictions, truelabels)
> perf
```

```
     DE
SEX     Yes    No
  Yes    19    60
  No     92 31055

Precision: 0.17
Recall: 0.24
F-measure: 0.2
```

```
> cacc <- fillCallingAcc(cacc, "UnNorm", "edgeRqlfDf1", perf)
```

- Normalized data with edgeR's own methodology and common/trended/tagwise moderation regime with prior.df=20 (default):

```
> truelabels <- rownames(resEdgeRnorm$table) %in% genderGenes
> predictions <- resEdgeRnorm$table$FDR < fdrCutoff &
                 abs(resEdgeRnorm$table$logFC) > log2(fcCutoff)
> perf <- performance(predictions, truelabels)
> perf
```

```
     DE
SEX     Yes    No
  Yes    25    54
  No    129 31018

Precision: 0.16
Recall: 0.32
F-measure: 0.21
```

```
> cacc <- fillCallingAcc(cacc, "OwnNorm", "edgeRdf20", perf)
```

- Normalized data with edgeR's own methodology and common/trended/tagwise moderation regime with prior.df=1:

```
> truelabels <- rownames(resEdgeRnormDf1$table) %in% genderGenes
> predictions <- resEdgeRnormDf1$table$FDR < fdrCutoff &
                 abs(resEdgeRnormDf1$table$logFC) > log2(fcCutoff)
> perf <- performance(predictions, truelabels)
> perf
```

```
     DE
SEX     Yes    No
  Yes    26    53
  No    113 31034

Precision: 0.19
Recall: 0.33
F-measure: 0.24
```

```
> cacc <- fillCallingAcc(cacc, "OwnNorm", "edgeRdf1", perf)
```

- Normalized data with edgeR's own methodology, common/trended/tagwise moderation regime with prior.df=20 (default) and quasi-likelihood F-tests:

```
> truelabels <- rownames(resEdgeRnormQLF$table) %in% genderGenes
> predictions <- resEdgeRnormQLF$table$FDR < fdrCutoff &
                 abs(resEdgeRnormQLF$table$logFC) > log2(fcCutoff)
> perf <- performance(predictions, truelabels)
> perf
```

```
     DE
SEX     Yes    No
  Yes    25    54
  No    115 31032

Precision: 0.18
Recall: 0.32
F-measure: 0.23
```

```
> cacc <- fillCallingAcc(cacc, "OwnNorm", "edgeRqlfDf20", perf)
```

- Normalized data with edgeR's own methodology and common/trended/tagwise moderation regime with `prior.df=1` and quasi-likelihood F-tests:

```
> truelabels <- rownames(resEdgeRnormQLFdf1$table) %in% genderGenes
> predictions <- resEdgeRnormQLFdf1$table$FDR < fdrCutoff &
                 abs(resEdgeRnormQLFdf1$table$logFC) > log2(fcCutoff)
> perf <- performance(predictions, truelabels)
> perf
```

```
     DE
SEX     Yes    No
  Yes    26    53
  No     92 31055

Precision: 0.22
Recall: 0.33
F-measure: 0.26
```

```
> cacc <- fillCallingAcc(cacc, "OwnNorm", "edgeRqlfDf1", perf)
```

- Normalized data with the cqn methodology and common/trended/tagwise moderation regime with `prior.df=20` (default):

```
> truelabels <- rownames(resEdgeRnormCQN$table) %in% genderGenes
> predictions <- resEdgeRnormCQN$table$FDR < fdrCutoff &
                 abs(resEdgeRnormCQN$table$logFC) > log2(fcCutoff)
> perf <- performance(predictions, truelabels)
> perf
```

```
     DE
SEX     Yes    No
  Yes    14    65
  No     71 27288

Precision: 0.16
Recall: 0.18
F-measure: 0.17
```

```
> cacc <- fillCallingAcc(cacc, "cqn", "edgeRdf20", perf)
```

- Normalized data with the cqn methodology and common/trended/tagwise moderation regime with `prior.df=1`:

```
> truelabels <- rownames(resEdgeRnormCQNdf1$table) %in% genderGenes
> predictions <- resEdgeRnormCQNdf1$table$FDR < fdrCutoff &
                 abs(resEdgeRnormCQNdf1$table$logFC) > log2(fcCutoff)
> perf <- performance(predictions, truelabels)
> perf
```

```
    DE
SEX     Yes    No
  Yes    14    65
  No     58 27301

Precision: 0.19
Recall: 0.18
F-measure: 0.19
```

```
> cacc <- fillCallingAcc(cacc, "cqn", "edgeRdf1", perf)
```

- Normalized data with the cqn methodology, common/trended/tagwise moderation regime with prior.df=20 (default) and quasi-likelihood F-tests:

```
> truelabels <- rownames(resEdgeRnormCQNqlf$table) %in% genderGenes
> predictions <- resEdgeRnormCQNqlf$table$FDR < fdrCutoff &
                 abs(resEdgeRnormCQNqlf$table$logFC) > log2(fcCutoff)
> perf <- performance(predictions, truelabels)
> perf
```

```
    DE
SEX     Yes    No
  Yes    14    65
  No     60 27299

Precision: 0.19
Recall: 0.18
F-measure: 0.18
```

```
> cacc <- fillCallingAcc(cacc, "cqn", "edgeRqlfDf20", perf)
```

- Normalized data with the cqn methodology, common/trended/tagwise moderation regime with prior.df=1 and quasi-likelihood F-tests:

```
> truelabels <- rownames(resEdgeRnormCQNqlfDf1$table) %in% genderGenes
> predictions <- resEdgeRnormCQNqlfDf1$table$FDR < fdrCutoff &
                 abs(resEdgeRnormCQNqlfDf1$table$logFC) > log2(fcCutoff)
> perf <- performance(predictions, truelabels)
> perf
```

```
    DE
SEX     Yes    No
  Yes    14    65
  No     53 27306

Precision: 0.21
Recall: 0.18
F-measure: 0.19
```

```
> cacc <- fillCallingAcc(cacc, "cqn", "edgeRqlfDf1", perf)
```

7.4 Comparison of all DE calling accuracies

In Table 2 we have summarized all the previous values on the calling accuracy of each normalization and DE detection method.

	UnNorm					OwnNorm					cqn				
	SexSp	DEyes	DEno	Total	Perf.	SexSp	DEyes	DEno	Total	Perf.	SexSp	DEyes	DEno	Total	Perf.
**tweeDEseq**	Yes	16	63	79	P=0.27	Yes	24	55	79	P=0.30	Yes	20	59	79	P=0.36
	No	43	31104	31147	R=0.20	No	56	31091	31147	R=0.30	No	35	27324	27359	R=0.25
	Total	59	31167	31226	F=0.23	Total	80	31146	31226	F=0.30	Total	55	27383	27438	F=0.30
	SexSp	DEyes	DEno	Total	Perf.	SexSp	DEyes	DEno	Total	Perf.	SexSp	DEyes	DEno	Total	Perf.
**DESeqCgO**	Yes	18	61	79	P=0.14	Yes	25	54	79	P=0.14	Yes	21	58	79	P=0.22
	No	111	31036	31147	R=0.23	No	153	30994	31147	R=0.32	No	74	27285	27359	R=0.27
	Total	129	31097	31226	F=0.17	Total	178	31048	31226	F=0.19	Total	95	27343	27438	F=0.24
	SexSp	DEyes	DEno	Total	Perf.	SexSp	DEyes	DEno	Total	Perf.	SexSp	DEyes	DEno	Total	Perf.
**DESeqCmax**	Yes	16	63	79	P=0.19	Yes	18	61	79	P=0.17	Yes	16	63	79	P=0.25
	No	69	31078	31147	R=0.20	No	85	31062	31147	R=0.23	No	47	27312	27359	R=0.20
	Total	85	31141	31226	F=0.20	Total	103	31123	31226	F=0.20	Total	63	27375	27438	F=0.23
	SexSp	DEyes	DEno	Total	Perf.	SexSp	DEyes	DEno	Total	Perf.	SexSp	DEyes	DEno	Total	Perf.
**DESeqPgO**	Yes	16	63	79	P=0.26	Yes	20	59	79	P=0.29	Yes	19	60	79	P=0.36
	No	45	31102	31147	R=0.20	No	50	31097	31147	R=0.25	No	34	27325	27359	R=0.24
	Total	61	31165	31226	F=0.23	Total	70	31156	31226	F=0.27	Total	53	27385	27438	F=0.29
	SexSp	DEyes	DEno	Total	Perf.	SexSp	DEyes	DEno	Total	Perf.	SexSp	DEyes	DEno	Total	Perf.
**DESeqPmax**	Yes	13	66	79	P=0.25	Yes	14	65	79	P=0.26	Yes	16	63	79	P=0.35
	No	40	31107	31147	R=0.16	No	39	31108	31147	R=0.18	No	30	27329	27359	R=0.20
	Total	53	31173	31226	F=0.20	Total	53	31173	31226	F=0.21	Total	46	27392	27438	F=0.26
	SexSp	DEyes	DEno	Total	Perf.	SexSp	DEyes	DEno	Total	Perf.	SexSp	DEyes	DEno	Total	Perf.
**edgeRdf20**	Yes	20	59	79	P=0.13	Yes	25	54	79	P=0.16	Yes	14	65	79	P=0.16
	No	137	31010	31147	R=0.25	No	129	31018	31147	R=0.32	No	71	27288	27359	R=0.18
	Total	157	31069	31226	F=0.17	Total	154	31072	31226	F=0.21	Total	85	27353	27438	F=0.17
	SexSp	DEyes	DEno	Total	Perf.	SexSp	DEyes	DEno	Total	Perf.	SexSp	DEyes	DEno	Total	Perf.
**edgeRdf1**	Yes	21	58	79	P=0.14	Yes	26	53	79	P=0.19	Yes	14	65	79	P=0.19
	No	128	31019	31147	R=0.27	No	113	31034	31147	R=0.33	No	58	27301	27359	R=0.18
	Total	149	31077	31226	F=0.18	Total	139	31087	31226	F=0.24	Total	72	27366	27438	F=0.19
	SexSp	DEyes	DEno	Total	Perf.	SexSp	DEyes	DEno	Total	Perf.	SexSp	DEyes	DEno	Total	Perf.
**edgeRqlfDf20**	Yes	19	60	79	P=0.14	Yes	25	54	79	P=0.18	Yes	14	65	79	P=0.19
	No	113	31034	31147	R=0.24	No	115	31032	31147	R=0.32	No	60	27299	27359	R=0.18
	Total	132	31094	31226	F=0.18	Total	140	31086	31226	F=0.23	Total	74	27364	27438	F=0.18
	SexSp	DEyes	DEno	Total	Perf.	SexSp	DEyes	DEno	Total	Perf.	SexSp	DEyes	DEno	Total	Perf.
**edgeRqlfDf1**	Yes	19	60	79	P=0.17	Yes	26	53	79	P=0.22	Yes	14	65	79	P=0.21
	No	92	31055	31147	R=0.24	No	92	31055	31147	R=0.33	No	53	27306	27359	R=0.18
	Total	111	31115	31226	F=0.20	Total	118	31108	31226	F=0.26	Total	67	27371	27438	F=0.19

Table 2. Comparison of different normalization and DE detection methods on the data from Pickrell *et al.* (2010) processed with our own pipeline. On the "Perf." column P denotes precision, R denotes recall and F denotes the F-measure.

Next, we examine the overall distribution of precision and recall values for every combination of normalization and DE detection method, shown in Figure 6.

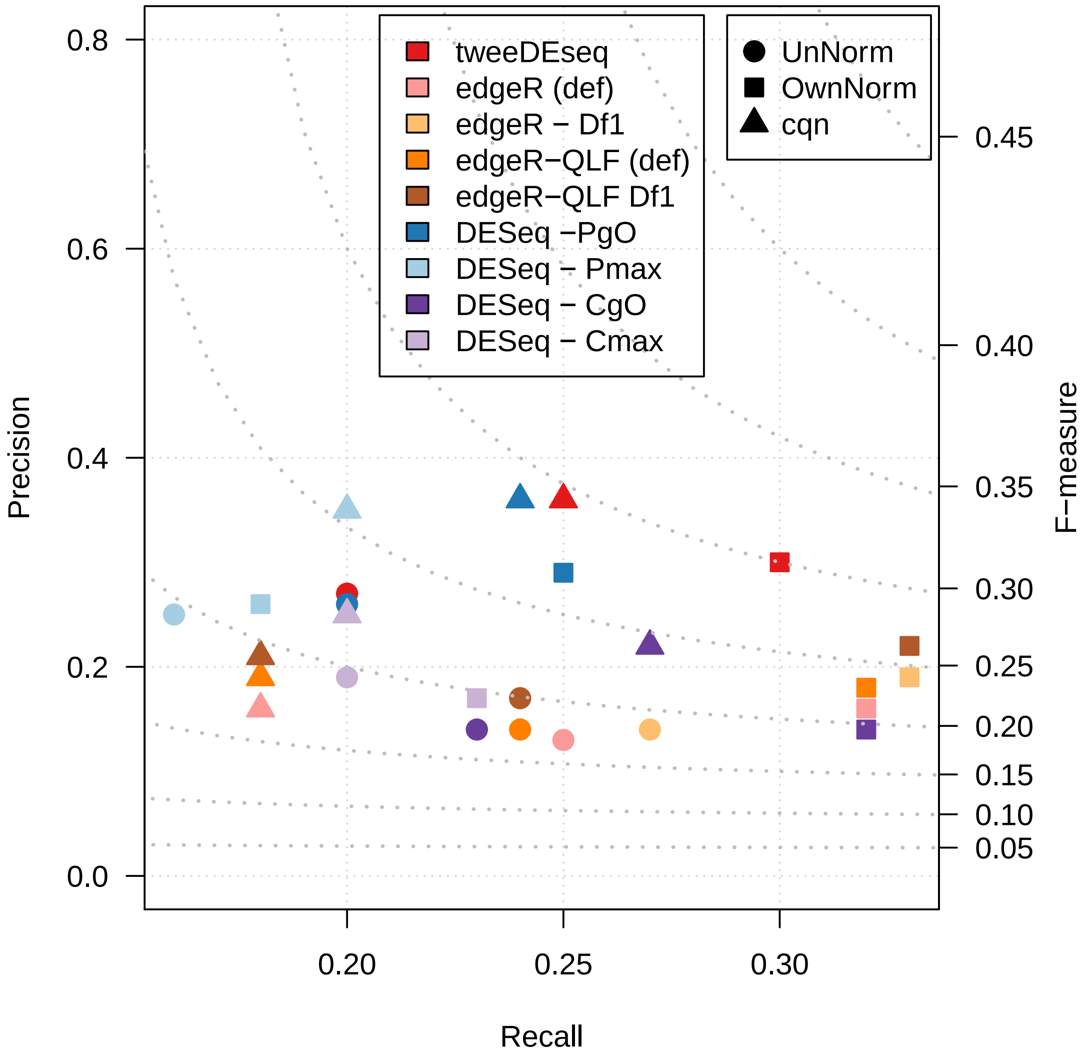

Figure 6. Precision (y-axis) as function of the recall (x-axis) for each combination of normalization and DE detection method. Right axis indicates values of the F-measure.

Finally, in Table 3 we show the list of DE genes found by tweeDEseq on the data normalized with the cqn package, which seems to be the most accurate one.

Table 3. Differentially expressed genes between female and male Nigerian individuals called by tweeDEseq at 10% FDR. Genes are ordered by their absolute fold-change in $\log_2$ scale. The "Gender specific" column indicates those genes reported in the literature as belonging to the male-specific region of the Y chromosome (MSYSkaletsky et al. (2003)) or escaping to the inactivation of the Xi chromosome (XiECarrel and HF (2005)).

	EnsemblID	Symbol	Chr	Description	Log2FC	GenderSpecific
1	ENSG00000229807	XIST	X	X (inactive)-specific transcript (non-protein coding)	8.39	XiE

Table 3 – Continued on next page

Table 3 – continued from previous page

2	ENSG00000131002	CYorf15B	Y	chromosome Y open reading frame 15B	-4.44	MSY
3	ENSG00000165246	NLGN4Y	Y	neuroligin 4, Y-linked	-3.90	MSY
4	ENSG00000099749	CYorf15A	Y	chromosome Y open reading frame 15A	-3.87	MSY
5	ENSG00000213318	RP11-331F4.1	16		-3.60	
6	ENSG00000233864	TTTY15	Y	testis-specific transcript, Y-linked 15 (non-protein coding)	-3.54	
7	ENSG00000157828	RPS4Y2	Y	ribosomal protein S4, Y-linked 2	-3.18	MSY
8	ENSG00000230986	RP13-204A15.1	X		-3.05	
9	ENSG00000146938	NLGN4X	X	neuroligin 4, X-linked	-3.04	XiE
10	ENSG00000129824	RPS4Y1	Y	ribosomal protein S4, Y-linked 1	-2.55	MSY
11	ENSG00000243209	AC010889.1	Y		-2.43	
12	ENSG00000198692	EIF1AY	Y	eukaryotic translation initiation factor 1A, Y-linked	-2.21	MSY
13	ENSG00000011201	KAL1	X	Kallmann syndrome 1 sequence	-1.81	
14	ENSG00000183878	UTY	Y	ubiquitously transcribed tetratricopeptide repeat gene, Y-linked	-1.75	MSY
15	ENSG00000241859	KALP	Y	Kallmann syndrome sequence pseudogene	-1.64	
16	ENSG00000067048	DDX3Y	Y	DEAD (Asp-Glu-Ala-Asp) box polypeptide 3, Y-linked	-1.59	MSY
17	ENSG00000232928	RP13-204A15.4	X		-1.42	
18	ENSG00000231535	NCRNA00278	Y	non-protein coding RNA 278	-1.38	
19	ENSG00000012817	KDM5D	Y	lysine (K)-specific demethylase 5D	-1.37	
20	ENSG00000006757	PNPLA4	X	patatin-like phospholipase domain containing 4	1.01	XiE
21	ENSG00000005302	MSL3	X	male-specific lethal 3 homolog (Drosophila)	0.91	
22	ENSG00000101846	STS	X	steroid sulfatase (microsomal), isozyme S	0.89	XiE
23	ENSG00000215520	RP11-401M16.3	1		0.83	
24	ENSG00000239254	RP11-365F18.3	7		0.83	

Table 3 – Continued on next page

Table 3 – continued from previous page

25	ENSG00000130021	HDHD1	X	haloacid dehalogenase-like hydrolase domain containing 1	0.82	XiE
26	ENSG00000224287	MSL3P1	2	male-specific lethal 3 homolog (Drosophila) pseudogene 1	0.81	
27	ENSG00000198034	RPS4X	X	ribosomal protein S4, X-linked	0.80	XiE
28	ENSG00000239490	RP11-863N1.1	18		0.80	
29	ENSG00000229920	AC016734.3	2		0.80	
30	ENSG00000214541	AL137162.1	20		0.80	
31	ENSG00000242058	RP11-143J12.1	18		0.77	
32	ENSG00000186008	RPS4XP21	19	ribosomal protein S4X pseudogene 21	0.76	
33	ENSG00000162639	HENMT1	1	HEN1 methyltransferase homolog 1 (Arabidopsis)	-0.76	
34	ENSG00000239830	CTD-3116E22.2	19		0.75	
35	ENSG00000243663	RP11-21K20.1	12		0.74	
36	ENSG00000219146	RP11-134L4.1	6		0.73	
37	ENSG00000226948	RP5-1068H6.3	20		0.72	
38	ENSG00000224892	RPS4XP16	13	ribosomal protein S4X pseudogene 16	0.72	
39	ENSG00000244097	RP11-411G7.1	17		0.72	
40	ENSG00000240371	RP11-624G17.1	11		0.71	
41	ENSG00000214203	RP11-135F9.1	12		0.71	
42	ENSG00000218265	RP11-501I19.4	6		0.71	
43	ENSG00000244073	CTD-2284O10.1	5		0.70	
44	ENSG00000234335	RP11-241I20.3	10		0.69	
45	ENSG00000240721	RPS4XP15	12	ribosomal protein S4X pseudogene 15	0.68	
46	ENSG00000205664	RP11-706O15.1	X	HCG1981372, isoform CRA_cNovel protein	0.65	
47	ENSG00000126012	KDM5C	X	lysine (K)-specific demethylase 5C	0.62	XiE
48	ENSG00000229305	RP11-431K24.2	1		0.57	
49	ENSG00000173674	EIF1AX	X	eukaryotic translation initiation factor 1A, X-linked	0.56	XiE
50	ENSG00000114374	USP9Y	Y	ubiquitin specific peptidase 9, Y-linked	-0.51	MSY
51	ENSG00000005889	ZFX	X	zinc finger protein, X-linked	0.49	XiE
52	ENSG00000086712	TXLNG	X	taxilin gamma	0.47	
53	ENSG00000215301	DDX3X	X	DEAD (Asp-Glu-Ala-Asp) box polypeptide 3, X-linked	0.46	XiE

Table 3 – Continued on next page

Table 3 – continued from previous page

54	ENSG00000067646	ZFY	Y	zinc finger protein, Y-linked	-0.45	MSY
55	ENSG00000147050	KDM6A	X	lysine (K)-specific demethylase 6A	0.43	

7.5 Session information for differential expression

```
> toLatex(sessionInfo())
```

- R version 2.15.1 (2012-06-22), x86_64-unknown-linux-gnu
- Locale: LC_CTYPE=en_US.UTF-8, LC_NUMERIC=C, LC_TIME=en_US.UTF-8, LC_COLLATE=en_US.UTF-8, LC_MONETARY=en_US.UTF-8, LC_MESSAGES=en_US.UTF-8, LC_PAPER=C, LC_NAME=C, LC_ADDRESS=C, LC_TELEPHONE=C, LC_MEASUREMENT=en_US.UTF-8, LC_IDENTIFICATION=C
- Base packages: base, datasets, graphics, grDevices, methods, parallel, splines, stats, utils
- Other packages: Biobase 2.18.0, BiocGenerics 0.4.0, cqn 1.4.0, DESeq 1.10.1, edgeR 3.0.8, lattice 0.20-10, limma 3.14.3, locfit 1.5-8, mclust 4.0, nor1mix 1.1-3, preprocessCore 1.20.0, quantreg 4.94, RColorBrewer 1.0-5, SparseM 0.96, tweeDEseq 1.4.0, tweeDEseqCountData 1.0.7, xtable 1.7-0
- Loaded via a namespace (and not attached): annotate 1.36.0, AnnotationDbi 1.20.3, DBI 0.2-5, genefilter 1.40.0, geneplotter 1.36.0, grid 2.15.1, IRanges 1.16.4, MASS 7.3-22, RSQLite 0.11.2, stats4 2.15.1, survival 2.37-2, tools 2.15.1, XML 3.95-0.1

8 Reproducibility in differential expression

In this section we assess the level of reproducibility of DE provided by each DE dectection method by using a subset of the LCL samples which has been profiled with microarrays and RNA-seq. Since we have to perform again DE analysis on different data sets, results will be stored in a different directory, concretely:

```
> cacheDir <- "resultsPickrell1huang"
> if (!file.exists(cacheDir))
      dir.create(cacheDir)
```

The microarray data was published by Huang et al. (2007) and processed to remove low quality chips and batch effects and to match Ensembl Gene identifiers and HapMap samples from the Pickrell et al. (2010) RNA-seq data. This results in two gene expression matrices with the same number of genes and samples stored under the name `commonPickrell1Huang` in the experimental data package tweeDEseqCountData.

```
> data(commonPickrell1Huang)
> stopifnot(dim(pickrell1countsCommon.eset) == dim(huangArrayRMAnoBatchCommon.eset))
> stopifnot(dim(pickrell1countsCQNcommon.eset) == dim(huangArrayRMAnoBatchCommon.eset))
> stopifnot(identical(featureNames(pickrell1countsCommon.eset), featureNames(huangArrayRMAnoBatch
> stopifnot(identical(sampleNames(pickrell1countsCommon.eset), sampleNames(huangArrayRMAnoBatchCo
> stopifnot(identical(featureNames(pickrell1countsCQNcommon.eset), featureNames(huangArrayRMAnoBa
> stopifnot(identical(sampleNames(pickrell1countsCQNcommon.eset), sampleNames(huangArrayRMAnoBat
> rawCountsCommon <- exprs(pickrell1countsCommon.eset)
> initialLibSizesCommon <- colSums(rawCountsCommon)
> countsCQNcommon <- exprs(pickrell1countsCQNcommon.eset)
```

```
> arrayRMAcommon <- exprs(huangArrayRMAnoBatchCommon.eset)
> genderCondition <- pickrell1countsCommon.eset$gender
> stopifnot(identical(huangArrayRMAnoBatchCommon.eset$gender, genderCondition))
```

8.1 Testing count data for differential expression with tweeDEseq

We perform a DE analysis with the `tweeDE()` function:

```
> cache(resTweeDEseqCQNcommon <- tweeDE(countsCQNcommon, group=genderCondition,
                                        pair=c("male", "female"), mc.cores=nCores),
        dir=cacheDir, prefix=cachePrefix)
> dim(resTweeDEseqCQNcommon)
```

8.2 Testing count data for differential expression with DESeq

In the case of DESeq, we estimate tagwise dispersions, perform negative binomial testing and call DE genes at the previously defined significance cutoffs.

- Using arguments `method="per-condition"` and `sharingMode="gene-est-only"`.

```
> cdsCQNcommon <- newCountDataSet(countsCQNcommon, genderCondition)
> sizeFactors(cdsCQNcommon) <- rep(1, ncol(countsCQNcommon))
> cache(cdsCQNcommonCgOnly <- estimateDispersions(cdsCQNcommon, method="per-condition", shar
        dir=cacheDir, prefix=cachePrefix)
> cache(resDESeqCQNcommonCgOnly <- nbinomTest(cdsCQNcommonCgOnly, "male", "female"),
        dir=cacheDir, prefix=cachePrefix)
> dim(resDESeqCQNcommonCgOnly)
```

- Using arguments `method="per-condition"` and `sharingMode="maximum"`. Here we need to set `fitType="local"` to avoid getting an error:

```
> cache(cdsCQNcommonCmax <- estimateDispersions(cdsCQNcommon, method="per-condition", sharin
        dir=cacheDir, prefix=cachePrefix)
> cache(resDESeqCQNcommonCmax <- nbinomTest(cdsCQNcommonCmax, "male", "female"),
        dir=cacheDir, prefix=cachePrefix)
> dim(resDESeqCQNcommonCmax)
```

- Using arguments `method="pooled"` and `sharingMode="gene-est-only"`.

```
> cache(cdsCQNcommonPgOnly <- estimateDispersions(cdsCQNcommon, method="pooled", sharingMode
        dir=cacheDir, prefix=cachePrefix)
> cache(resDESeqCQNcommonPgOnly <- nbinomTest(cdsCQNcommonPgOnly, "male", "female"),
        dir=cacheDir, prefix=cachePrefix)
> dim(resDESeqCQNcommonPgOnly)
```

- Using arguments `method="pooled"` and `sharingMode="maximum"`. Here we need to set `fit-Type="local"` to avoid getting an error:

```
> cache(cdsCQNcommonPmax <- estimateDispersions(cdsCQNcommon, method="pooled", sharingMode="
        dir=cacheDir, prefix=cachePrefix)
> cache(resDESeqCQNcommonPmax <- nbinomTest(cdsCQNcommonPmax, "male", "female"),
        dir=cacheDir, prefix=cachePrefix)
> dim(resDESeqCQNcommonPmax)
```

8.3 Testing count data for differential expression with edgeR

In the case of edgeR, we estimate tagwise dispersions, perform negative binomial testing and call DE genes at the previously defined significance cutoffs.

- Common/trended/tagwise moderation regime with `prior.n=20` (default):

```
> dCQN <- DGEList(counts=rawCountsCommon, group=genderCondition, lib.size=initialLibSizesCom
> design <- model.matrix(~ dCQN$sample$group)
> cache(dCQN.common <- estimateGLMCommonDisp(dCQN, design, offset=cqnNormCommon$offset),
        dir=cacheDir, prefix=cachePrefix)
> cache(dCQN.trended <- estimateGLMTrendedDisp(dCQN.common, design, offset=cqnNormCommon$off
        dir=cacheDir, prefix=cachePrefix)
> cache(dCQN.tagwise <- estimateGLMTagwiseDisp(dCQN.trended, design, offset=cqnNormCommon$of
        dir=cacheDir, prefix=cachePrefix)
> cache(dCQN.fit <- glmFit(dCQN.tagwise, design, offset=cqnNormCommon$offset),
        dir=cacheDir, prefix=cachePrefix)
> cache(dCQN.lrt <- glmLRT(dCQN.fit, coef=2), dir=cacheDir, prefix=cachePrefix)
> cache(resEdgeRnormCQN <- topTags(dCQN.lrt, n=Inf), dir=cacheDir, prefix=cachePrefix)
> dim(resEdgeRnormCQN)

[1] 15194     5
```

- Common/trended/tagwise moderation regime with `prior.n=1`:

```
> cache(dCQN.tagwise.df1 <- estimateGLMTagwiseDisp(dCQN.trended, design, prior.df=1, offset=
        dir=cacheDir, prefix=cachePrefix)
> cache(dCQN.fit.df1 <- glmFit(dCQN.tagwise.df1, design, offset=cqnNormCommon$offset),
        dir=cacheDir, prefix=cachePrefix)
> cache(dCQN.lrt.df1 <- glmLRT(dCQN.fit.df1, coef=2), dir=cacheDir, prefix=cachePrefix)
> cache(resEdgeRnormCQNdf1 <- topTags(dCQN.lrt.df1, n=Inf), dir=cacheDir, prefix=cachePrefix
> dim(resEdgeRnormCQNdf1)

[1] 15194     5
```

- Common/trended/tagwise moderation regime with `prior.df=20` (default) and quasi-likelihood F-tests:

```
> cache(dCQN.qlf <- glmQLFTest(dCQN.fit, coef=2), dir=cacheDir, prefix=cachePrefix)
> cache(resEdgeRnormCQNqlf <- topTags(dCQN.qlf, n=Inf), dir=cacheDir, prefix=cachePrefix)
> dim(resEdgeRnormCQNqlf)

[1] 15194     5
```

- Normalized data with the cqn methodology, common/trended/tagwise moderation regime with `prior.df=20` (default) and quasi-likelihood F-tests:

```
> cache(dCQN.df1.qlf <- glmQLFTest(dCQN.fit.df1, coef=2), dir=cacheDir, prefix=cachePrefix)
> cache(resEdgeRnormCQNqlfDf1 <- topTags(dCQN.df1.qlf, n=Inf), dir=cacheDir, prefix=cachePre
> dim(resEdgeRnormCQNqlfDf1)

[1] 15194     5
```

8.4 Testing microarray data for differential expression with limma

```
> design <- model.matrix(~ genderCondition)
> fit <- lmFit(arrayRMAcommon, design)
> fit <- eBayes(fit)
> resLimmaRMA <- topTable(fit, coef=2, n=Inf)
> dim(resLimmaRMA)
[1] 15194     7
```

8.5 Comparison of reproducibility of significance levels of differential expression

We want to assess the reproducibility of DE significance levels by each DE detection method in microarray and RNA-seq data. For that purpose, we will compare raw *p*-values from each different method on DE genes between male and female LCL samples called at 10% FDR:

```
> fdrCutoff <- 0.1
```

We will use again the genes with documented sex-specific expression to highlight those most likely to be bona fide DE genes:

```
> length(XiEgenes)
[1] 63
> length(msYgenes)
[1] 32
> length(genderGenes)
[1] 95
```

where the latter vector `genderGenes` contains the first two, `XiEgenes` and `msYgenes`, containing Ensembl Gene IDs from the two separated lists of XiE and MSY genes, respectively. We start by collecting the raw and adjusted *p*-values of DE between male and female LCL samples for every DE detection method on microarray (limma) and RNA-seq data (tweeDEseq, DESeq and edgeR):

```
> allGenes <- rownames(rawCountsCommon)
> length(allGenes)
[1] 15194
> allP <- data.frame(tweeDEseq=rep(NA, length(allGenes)),
                     DESeqCgOn=rep(NA, length(allGenes)),
                     DESeqCmax=rep(NA, length(allGenes)),
                     DESeqPgOn=rep(NA, length(allGenes)),
                     DESeqPmax=rep(NA, length(allGenes)),
                     edgeRdf20=rep(NA, length(allGenes)),
                     edgeRdf1=rep(NA, length(allGenes)),
                     edgeRqlfDf20=rep(NA, length(allGenes)),
                     edgeRqlfDf1=rep(NA, length(allGenes)),
                     limma=rep(NA, length(allGenes)),
                     row.names=allGenes)
> allP$tweeDEseq <- resTweeDEseqCQNcommon[allGenes, "pval"]
> allP$DESeqCgOn <- resDESeqCQNcommonCgOnly[match(allGenes, resDESeqCQNcommonCgOnly$id), "pval"]
> allP$DESeqCmax <- resDESeqCQNcommonCmax[match(allGenes, resDESeqCQNcommonCmax$id), "pval"]
> allP$DESeqPgOn <- resDESeqCQNcommonPgOnly[match(allGenes, resDESeqCQNcommonPgOnly$id), "pval"]
> allP$DESeqPmax <- resDESeqCQNcommonPmax[match(allGenes, resDESeqCQNcommonPmax$id), "pval"]
> allP$edgeRdf20 <- resEdgeRnormCQN$table[allGenes, "PValue"]
> allP$edgeRdf1 <- resEdgeRnormCQNdf1$table[allGenes, "PValue"]
> allP$edgeRqlfDf20 <- resEdgeRnormCQNqlf$table[allGenes, "PValue"]
```

```
> allP$edgeRqlfDf1 <- resEdgeRnormCQNqlfDf1$table[allGenes, "PValue"]
> allP$limma <- resLimmaRMA[match(allGenes, resLimmaRMA$ID), "P.Value"]
> allP <- as.data.frame(apply(allP, 2, function(x) -log10(x)))
> allFDR <- data.frame(tweeDEseq=rep(NA, length(allGenes)),
                     DESeqCgOn=rep(NA, length(allGenes)),
                     DESeqCmax=rep(NA, length(allGenes)),
                     DESeqPgOn=rep(NA, length(allGenes)),
                     DESeqPmax=rep(NA, length(allGenes)),
                     edgeRdf20=rep(NA, length(allGenes)),
                     edgeRdf1=rep(NA, length(allGenes)),
                     edgeRqlfDf20=rep(NA, length(allGenes)),
                     edgeRqlfDf1=rep(NA, length(allGenes)),
                     limma=rep(NA, length(allGenes)),
                     row.names=allGenes)
> allFDR$tweeDEseq <- resTweeDEseqCQNcommon[allGenes, "pval.adjust"]
> allFDR$DESeqCgOn <- resDESeqCQNcommonCgOnly[match(allGenes, resDESeqCQNcommonCgOnly$id), "padj[illegible]
> allFDR$DESeqCmax <- resDESeqCQNcommonCmax[match(allGenes, resDESeqCQNcommonCmax$id), "padj"]
> allFDR$DESeqPgOn <- resDESeqCQNcommonPgOnly[match(allGenes, resDESeqCQNcommonPgOnly$id), "padj[illegible]
> allFDR$DESeqPmax <- resDESeqCQNcommonPmax[match(allGenes, resDESeqCQNcommonPmax$id), "padj"]
> allFDR$edgeRdf20 <- resEdgeRnormCQN$table[allGenes, "FDR"]
> allFDR$edgeRdf1 <- resEdgeRnormCQNdf1$table[allGenes, "FDR"]
> allFDR$edgeRqlfDf20 <- resEdgeRnormCQNqlf$table[allGenes, "FDR"]
> allFDR$edgeRqlfDf1 <- resEdgeRnormCQNqlfDf1$table[allGenes, "FDR"]
> allFDR$limma <- resLimmaRMA[match(allGenes, resLimmaRMA$ID), "adj.P.Val"]
> dim(allP)

[1] 15194    10

> dim(allFDR)

[1] 15194    10
```

We compare first the $-\log_{10}$ units of raw p-values between limma (microarray) and every other RNA-seq DE detection methodology for genes called DE at 10% FDR by *either* of the two compared methods. These comparisons are shown in Figure 7.

Finally, we compare the $-\log_{10}$ units of raw p-values between limma (microarray) and every other RNA-seq DE detection methodology for genes called DE at 10% FDR by *both* of the two compared methods. These comparisons are shown in Figure 8.

8.6 Comparing RNA-seq and microarray DE analyses

We are also interested in highlighting differences and challenges in DE analyses with RNA-seq with respect to microarray data. For this purpose, we use again the same two analogous data sets but, this time we only enforce matching the samples between the two gene expression data matrices and we only compare `tweeDEseq` on the RNA-seq data normalized with `cqn`. These two other gene expression matrices form part also from the objects loaded under the name `commonPickrell1huang`:

```
> stopifnot(ncol(pickrell1countsCommonSamples.eset) == ncol(huangArrayRMAnoBatchCommonSamples.es[illegible]
> stopifnot(ncol(pickrell1countsCQNcommonSamples.eset) == ncol(huangArrayRMAnoBatchCommonSamples.[illegible]
> stopifnot(identical(sampleNames(pickrell1countsCommonSamples.eset), sampleNames(huangArrayRMAn[illegible]
> stopifnot(identical(sampleNames(pickrell1countsCQNcommonSamples.eset), sampleNames(huangArrayRM[illegible]
> rawCountsCommonSamples <- exprs(pickrell1countsCommonSamples.eset)
> initialLibSizesCommonSamples <- colSums(rawCountsCommonSamples)
> countsCQNcommonSamples <- exprs(pickrell1countsCQNcommonSamples.eset)
> arrayRMAcommonSamples <- exprs(huangArrayRMAnoBatchCommonSamples.eset)
> genderCondition <- pickrell1countsCommonSamples.eset$gender
> dim(countsCQNcommonSamples)

[1] 27438    36
```

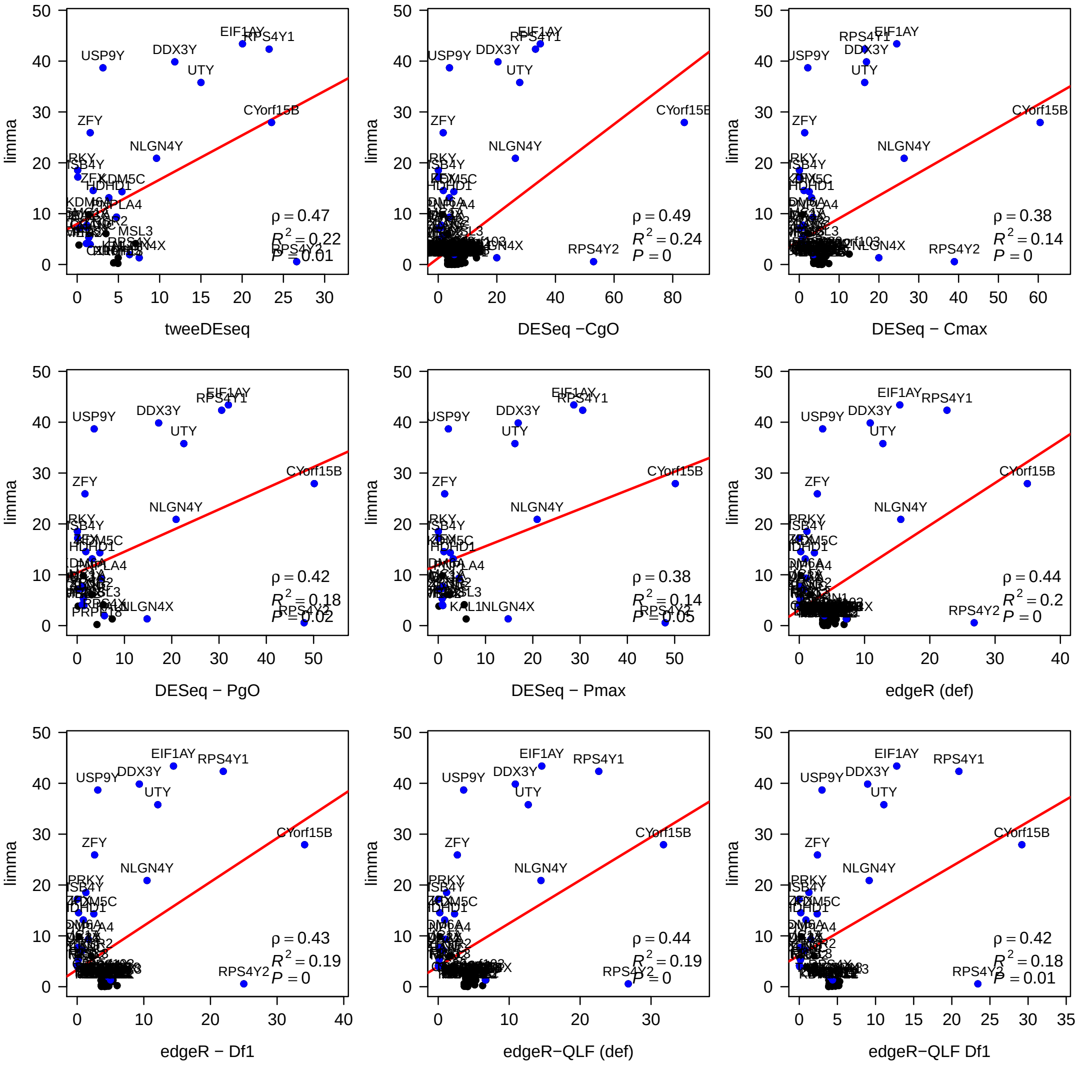

Figure 7. Raw p-values of differential expression in $-\log_{10}$ scale for DE genes called at 10% FDR by either limma (y-axis), from microarray data, *or* the other compared DE detection method applied on RNA-seq data (x-axis). A regression line is depicted in red. On the bottom-right corner of each panel, ρ indicates the Pearson correlation whereas R^2 and P indicate, respectively, the coefficient of determination and p-value of the test for zero regression coefficient, of the significance values of limma as function of those from the compared RNA-seq method. Even though the relationships are significant in all comparisons, the low R^2 values indicate a poor level of reproducibility between microarray and RNA-seq DE analysis, irrespectively of the method employed for detecting DE genes in RNA-seq data. A large fraction of irreproducible DE is due to genes that are called DE by one technology but not by the other. Blue dots indicate genes with documented sex-specific expression.

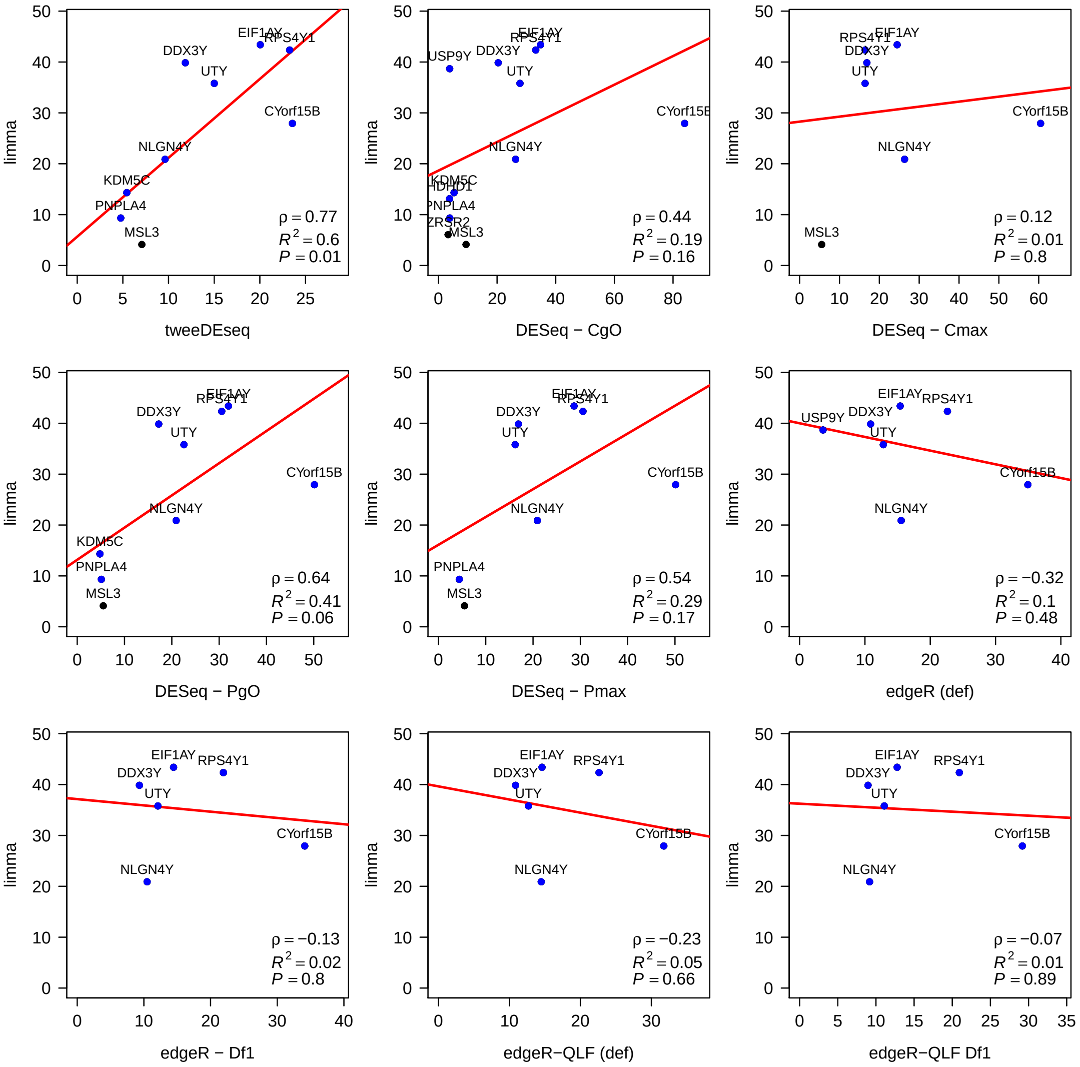

Figure 8. Raw p-values of differential expression in $-\log_{10}$ scale for DE genes called at 10% FDR by both, limma (y-axis), from microarray data, *and* the other compared DE detection method applied on RNA-seq data (x-axis). A regression line is depicted in red. On the bottom-right corner of each panel, ρ indicates the Pearson correlation whereas R^2 and P indicate, respectively, the coefficient of determination and p-value of the test for zero regression coefficient, of the $-\log_{10} p$-values of limma as function of those from the compared RNA-seq method. Only tweeDEseq provides a significant ($p < 0.05$) level of reproducibility between p-values of DE genes reported by both, limma on microarray data and the compared RNA-seq method, attaining also the highest ρ and R^2 values. Blue dots indicate genes with documented sex-specific expression.

We start by applying tweeDEseq:

```
> cache(resTweeDEseqCQNcommonSamples <- tweeDE(countsCQNcommonSamples, group=genderCondition,
                                        pair=c("male", "female"), mc.cores=nCores),
        dir=cacheDir, prefix=cachePrefix)
> dim(resTweeDEseqCQNcommonSamples)
[1] 27438     7
```

Now limma:

```
> design <- model.matrix(~ genderCondition)
> dim(arrayRMAcommonSamples)
[1] 16323    36
> fit <- lmFit(arrayRMAcommonSamples, design)
> fit <- eBayes(fit)
> resLimmaRMAcommonSamples <- topTable(fit, coef=2, n=Inf)
> dim(resLimmaRMAcommonSamples)
[1] 16323     7
```

and compare these two DE analyses by means of volcano plots shown in Figure 9.

8.7 Session information for reproducibility in differential expression

```
> toLatex(sessionInfo())
```

- R version 2.15.1 (2012-06-22), x86_64-unknown-linux-gnu
- Locale: LC_CTYPE=en_US.UTF8, LC_NUMERIC=C, LC_TIME=en_US.UTF8, LC_COLLATE=en_US.UTF8, LC_MONETARY=en_US.UTF8, LC_MESSAGES=en_US.UTF8, LC_PAPER=C, LC_NAME=C, LC_ADDRESS=C, LC_TELEPHONE=C, LC_MEASUREMENT=en_US.UTF8, LC_IDENTIFICATION=C
- Base packages: base, datasets, graphics, grDevices, methods, parallel, stats, utils
- Other packages: Biobase 2.18.0, BiocGenerics 0.4.0, DESeq 1.10.1, edgeR 3.0.8, lattice 0.20-14, limma 3.14.4, locfit 1.5-8, RColorBrewer 1.0-5, tweeDEseq 1.4.1, tweeDEseqCountData 1.0.8, xtable 1.7-1
- Loaded via a namespace (and not attached): annotate 1.36.0, AnnotationDbi 1.20.7, cqn 1.4.0, DBI 0.2-5, genefilter 1.40.0, geneplotter 1.36.0, grid 2.15.1, IRanges 1.16.6, MASS 7.3-23, nor1mix 1.1-3, RSQLite 0.11.2, splines 2.15.1, stats4 2.15.1, survival 2.37-4, tools 2.15.1, XML 3.95-0.2

References

Carrel, L. and HF, W. (2005). X-inactivation profile reveals extensive variability in X-linked gene expression in females. *Nature*, 434:400–404.

Eisenberg, E. and Levanon, E. Y. (2003). Human housekeeping genes are compact. *Trends Genet*, 19(7):362–5.

Hansen, K., Irizarry, R., and Wu, Z. (2011). Removing technical variability in rna-seq data using conditional quantile normalization. Technical Report 227, Department of Biostatistics, Johns Hopkins University, Baltimore.

Huang, R. S., Duan, S., Bleibel, W. K., Kistner, E. O., Zhang, W., Clark, T. A., Chen, T. X., Schweitzer, A. C., Blume, J. E., Cox, N. J., and Dolan, M. E. (2007). A genome-wide approach to identify genetic variants that contribute to etoposide-induced cytotoxicity. *Proceedings of the National Academy of Sciences of the United States of America*, 104(23):9758–9763.

McCall, M., Uppal, K., Jaffee, H., and Zilliox, MJ Irizarry, R. (2011). The gene expression barcode: leveraging public data repositories to begin cataloging the human and murine transcriptomes. *Nucleic Acids Res*, 39:D1011–D1015.

Pickrell, J., Marioni, J., Pai, A., Degner, J., Engelhardt, B., Nkadori, E., Veyrieras, J., Stephens, M., Gilad, Y., and Pritchard, J. (2010). Understanding mechanisms underlying human gene expression variation with RNA sequencing. *Nature*, 464:768–772.

Robinson, M. and Oshlack, A. (2010). A scaling normalization method for differential expression analysis of RNA-seq data. *Genome Biol*, page 11:R25.

Skaletsky, H., Kuroda-Kawaguchi, T., Minx, P., Cordum, H., Hillier, L., Brown, L., Repping, S., Pyntikova, T., Ali, J., Bieri, T., Chinwalla, A., Delehaunty, A., Delehaunty, K., Du, H., Fewell, G., Fulton, L., Fulton, R., Graves, T., Hou, S.-F., Latrielle, P., Leonard, S., Mardis, E., Maupin, R., McPherson, J., Miner, T., Nash, W., Nguyen, C., Ozersky, P., Pepin, K., Rock, S., Rohlfing, T., Scott, K., Schultz, B., Strong, C., Tin-Wollam, A., Yang, S.-P., Waterston, R., Wilson, R., Rozen, S., and Page, D. (2003). The male-specific region of the human y chromosome is a mosic of discrete sequence classes. *Nature*, 423:825–837.

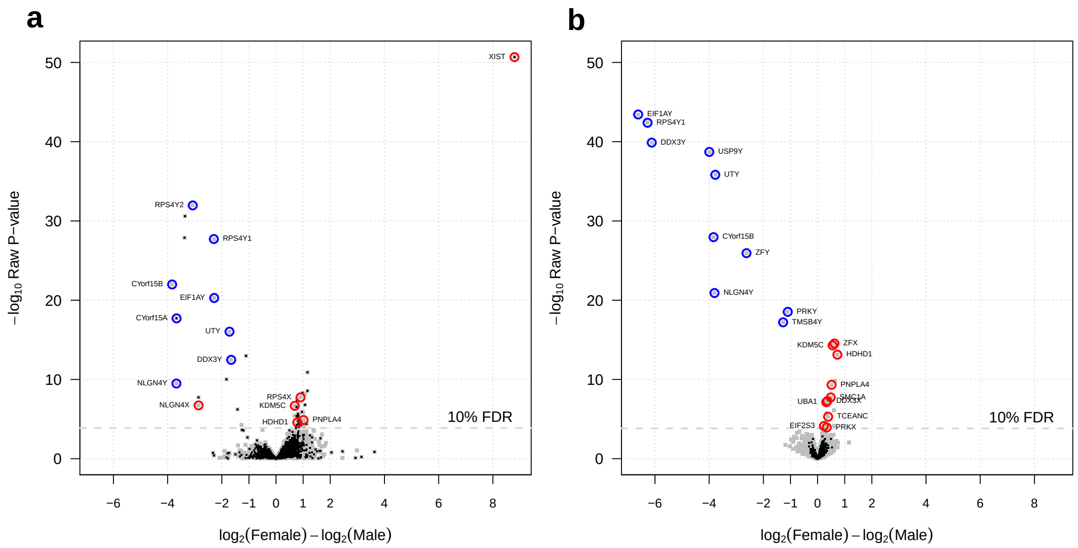

Figure 9. Comparison of DE analyses between microarray and RNA-seq. Volcano plots of DE analyses performed on matching LCL samples profiled with RNA-seq (a) and gene expression microarrays (b). The x-axis corresponds to $\log_2$ fold-changes between female and male individuals while the y-axis corresponds to $-\log_{10} P$-value of significance. RNA-seq data were analysed with `tweeDEseq` while microarray data were analysed with `limma`. Grey dots indicate genes common to both, the RNA-seq and the microarray gene expression matrices, while black dots indicate genes occurring exclusively in one of the two data sets. Blue and red circles indicate genes documented in the literature with sex-specific expression, concretely belonging to the male-specific region of chromosome Y and escaping X-chromosome inactivation in females, respectively.
