# Supplementary figures and images for: A flexible count data model to fit the wide diversity of expression profiles arising from extensively replicated RNA-seq experiments
Source: BMC Bioinformatics. 2013 Aug 21;14:254. doi: 10.1186/1471-2105-14-254 (PMC3849762; doi:10.1186/1471-2105-14-254)

Fold-change 1.5

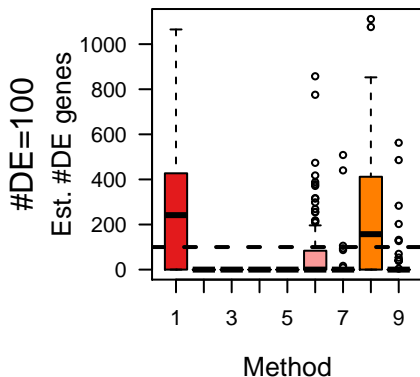

Fold-change 2.0

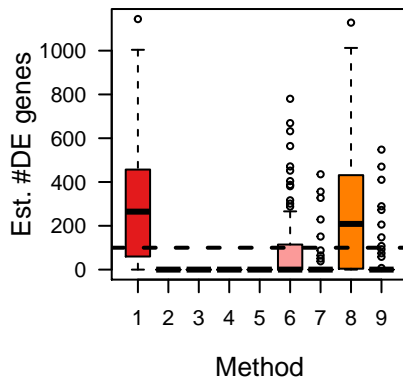

Fold-change 4.0

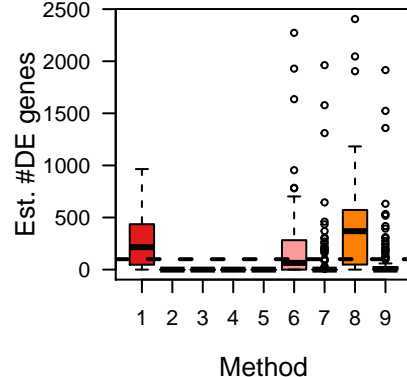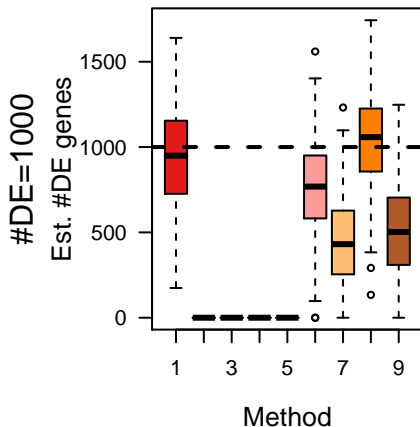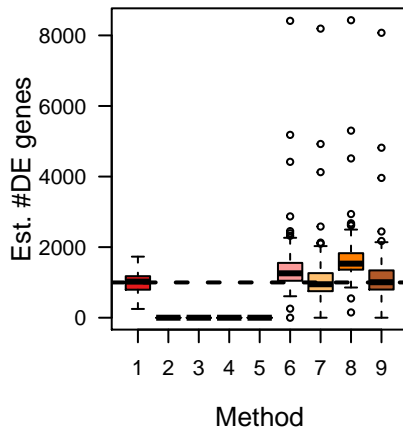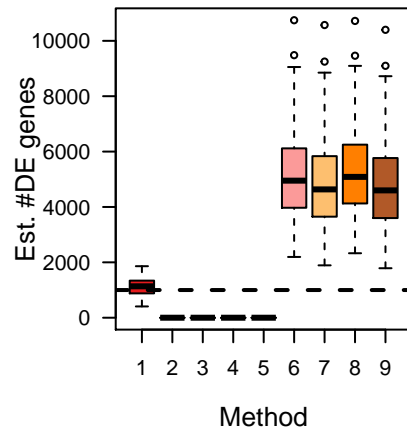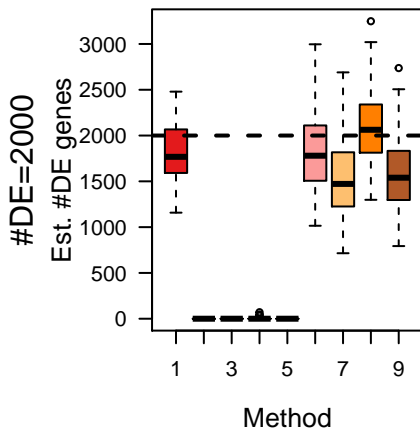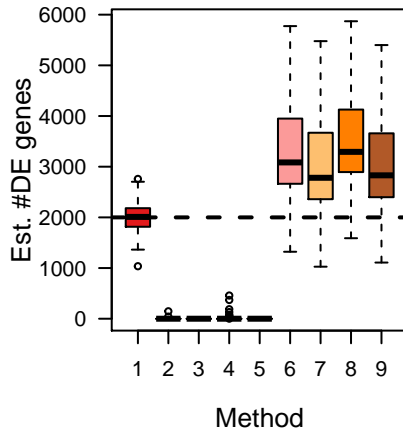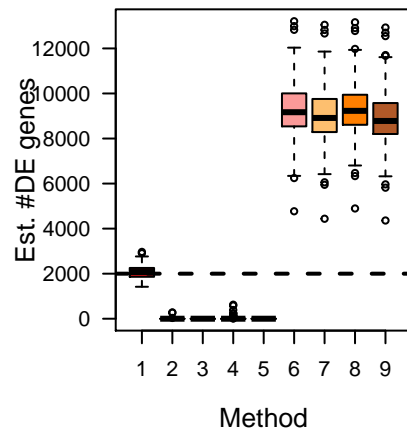

Supplement: Additional file 1 — Scripts. ZIP file (.zip) containing all scripts, in the form of Sweave vignettes, to reproduce the results shown in this paper, including one copy of the resulting PDF file. Please read first through the README file contained in this tar ball in order to understand how to run the scripts. [file 1471-2105-14-254-S1.zip › tweeDEseqSupportingAnalyses/DEsimulations/estNrDEgenes.sdoff0.0.pdf]

Fold-change 1.5

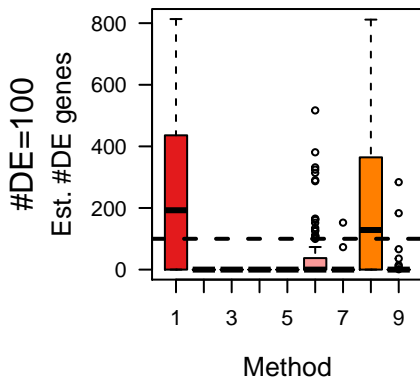

Fold-change 2.0

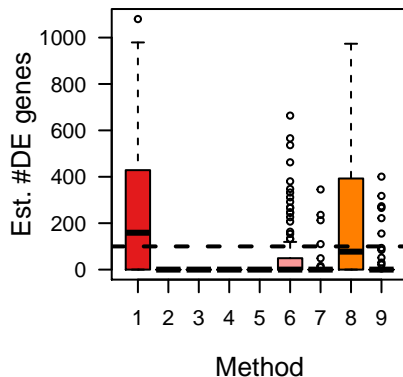

Fold-change 4.0

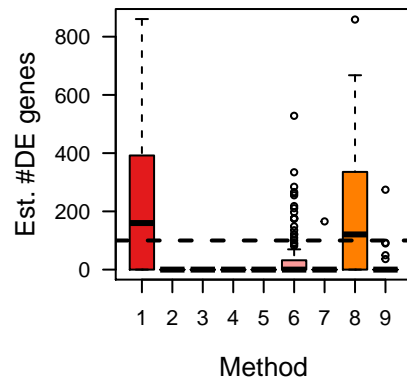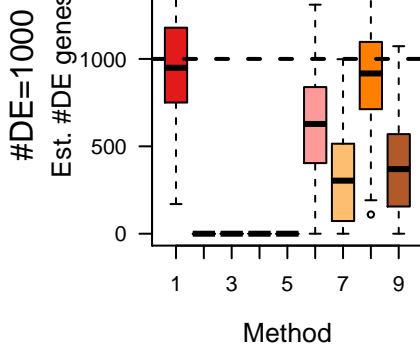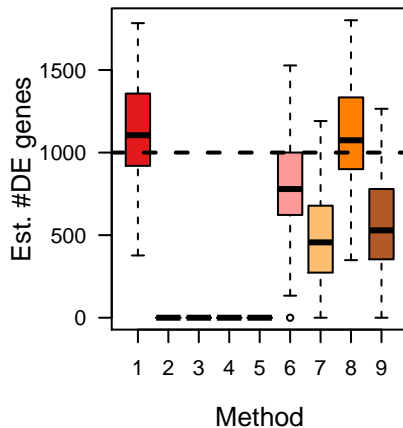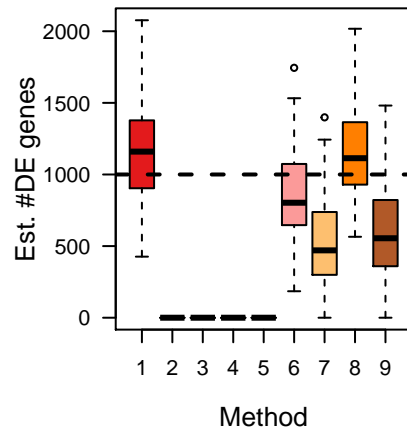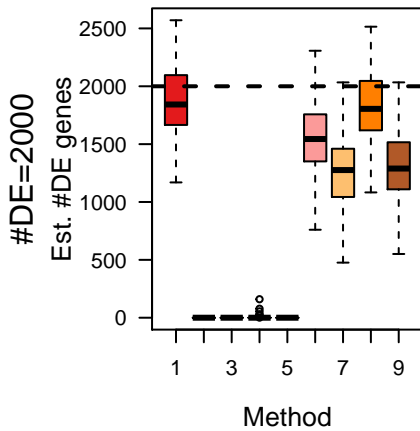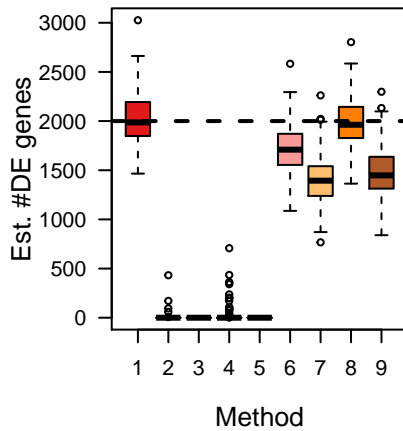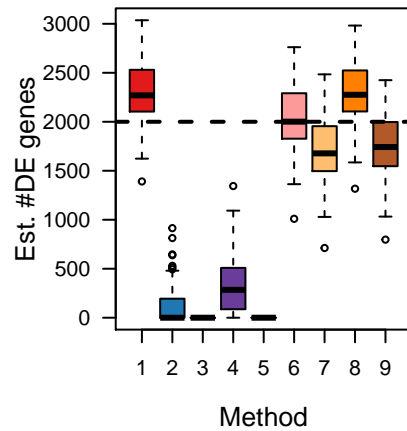

Supplement: Additional file 1 — Scripts. ZIP file (.zip) containing all scripts, in the form of Sweave vignettes, to reproduce the results shown in this paper, including one copy of the resulting PDF file. Please read first through the README file contained in this tar ball in order to understand how to run the scripts. [file 1471-2105-14-254-S1.zip › tweeDEseqSupportingAnalyses/DEsimulations/estNrDEgenes.sdoff0.5.pdf]

Est. #DE genes / True #DE genes

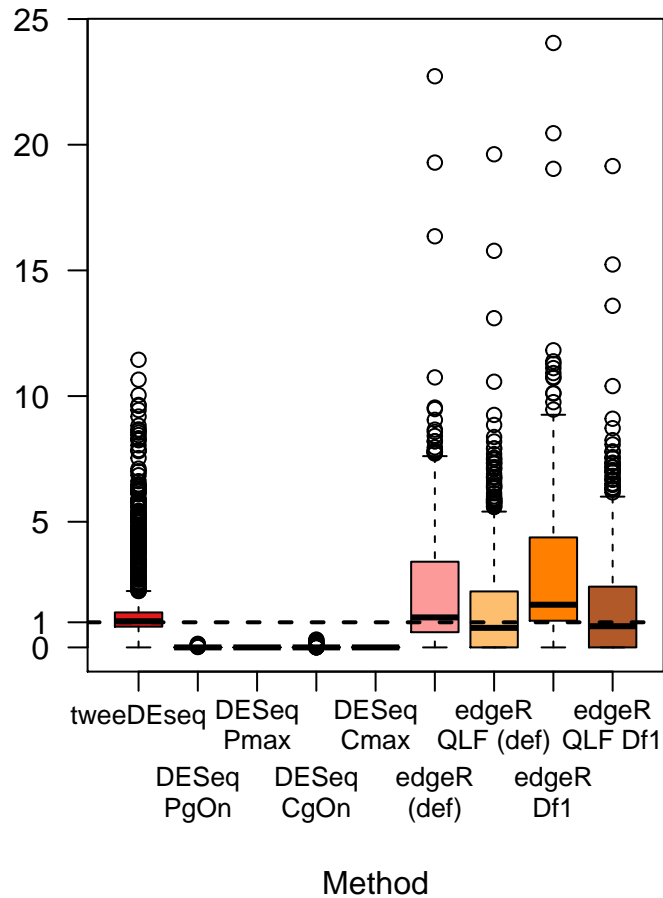

Est. #DE genes / True #DE genes

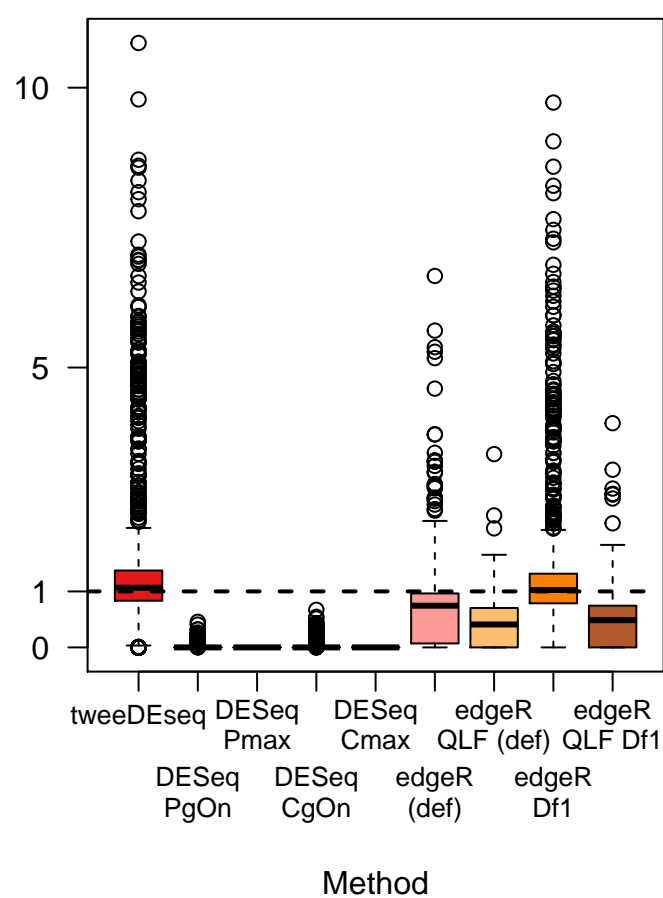

Supplement: Additional file 1 — Scripts. ZIP file (.zip) containing all scripts, in the form of Sweave vignettes, to reproduce the results shown in this paper, including one copy of the resulting PDF file. Please read first through the README file contained in this tar ball in order to understand how to run the scripts. [file 1471-2105-14-254-S1.zip › tweeDEseqSupportingAnalyses/DEsimulations/estRatioNrDEgenes.pdf]

Fold-change 1.5

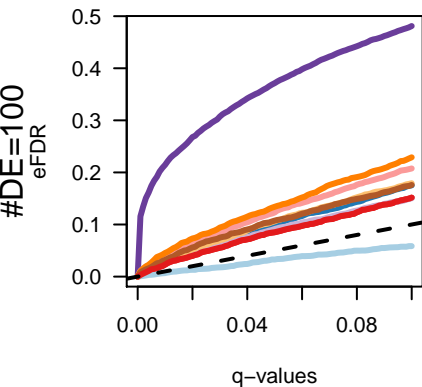

Fold-change 2.0

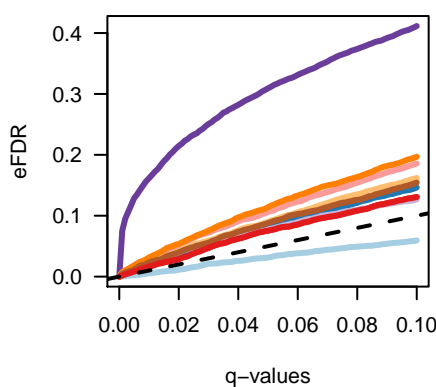

Fold-change 4.0

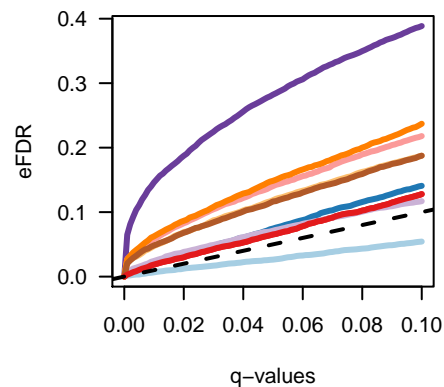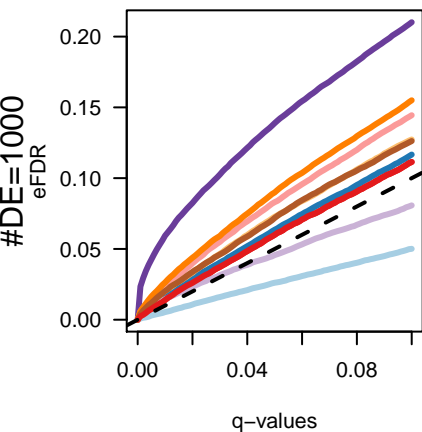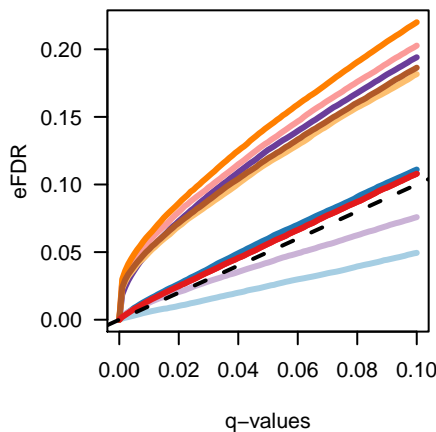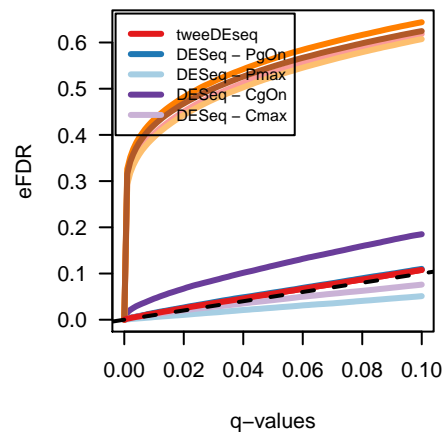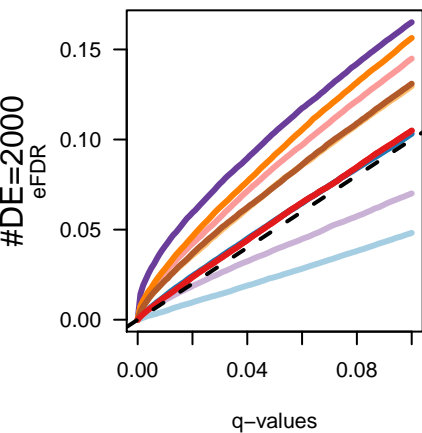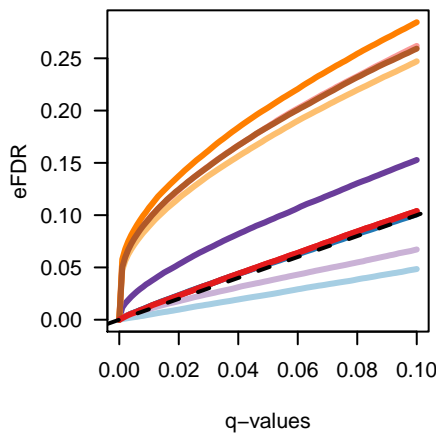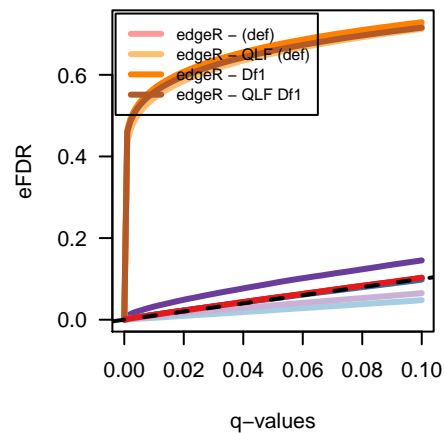

Supplement: Additional file 1 — Scripts. ZIP file (.zip) containing all scripts, in the form of Sweave vignettes, to reproduce the results shown in this paper, including one copy of the resulting PDF file. Please read first through the README file contained in this tar ball in order to understand how to run the scripts. [file 1471-2105-14-254-S1.zip › tweeDEseqSupportingAnalyses/DEsimulations/qvalXeFDR.sdoff0.0.pdf]

Fold-change 1.5

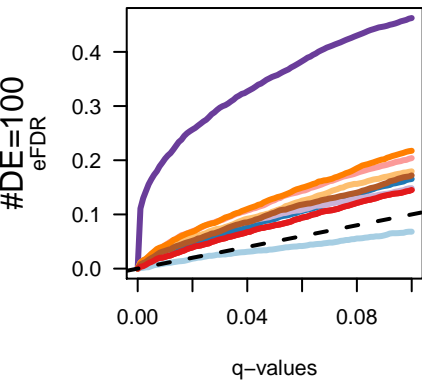

Fold-change 2.0

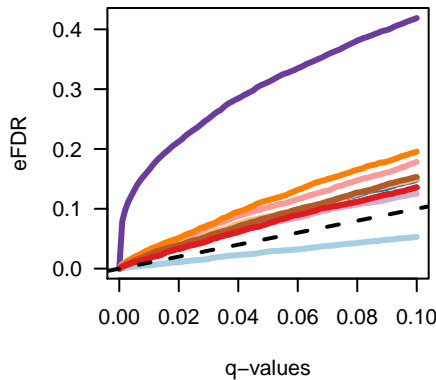

Fold-change 4.0

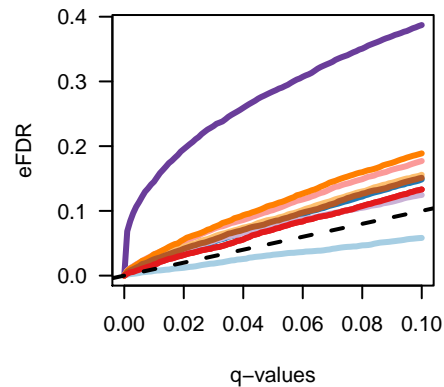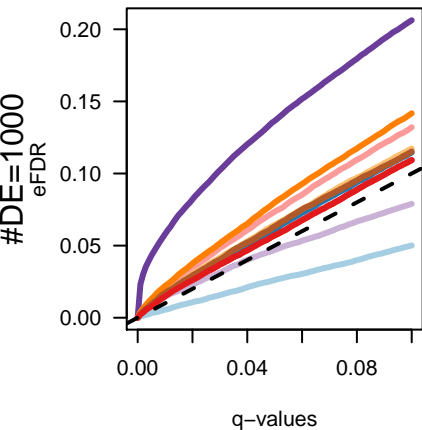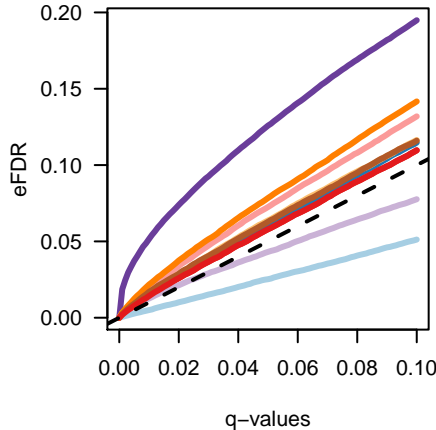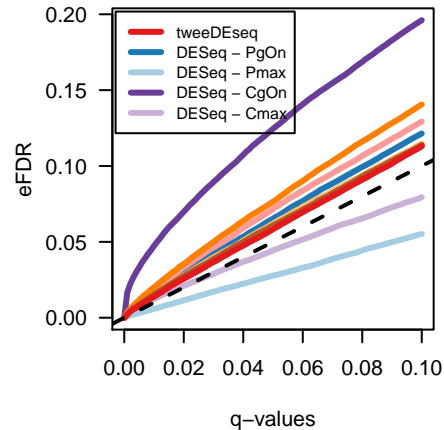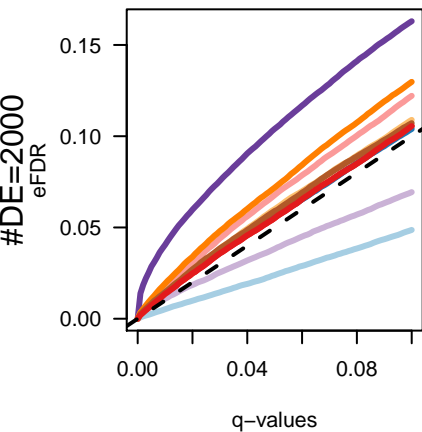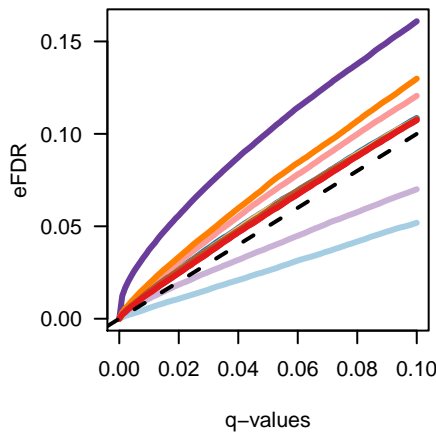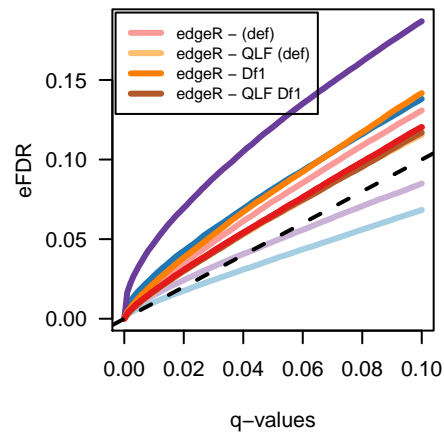

Supplement: Additional file 1 — Scripts. ZIP file (.zip) containing all scripts, in the form of Sweave vignettes, to reproduce the results shown in this paper, including one copy of the resulting PDF file. Please read first through the README file contained in this tar ball in order to understand how to run the scripts. [file 1471-2105-14-254-S1.zip › tweeDEseqSupportingAnalyses/DEsimulations/qvalXeFDR.sdoff0.5.pdf]

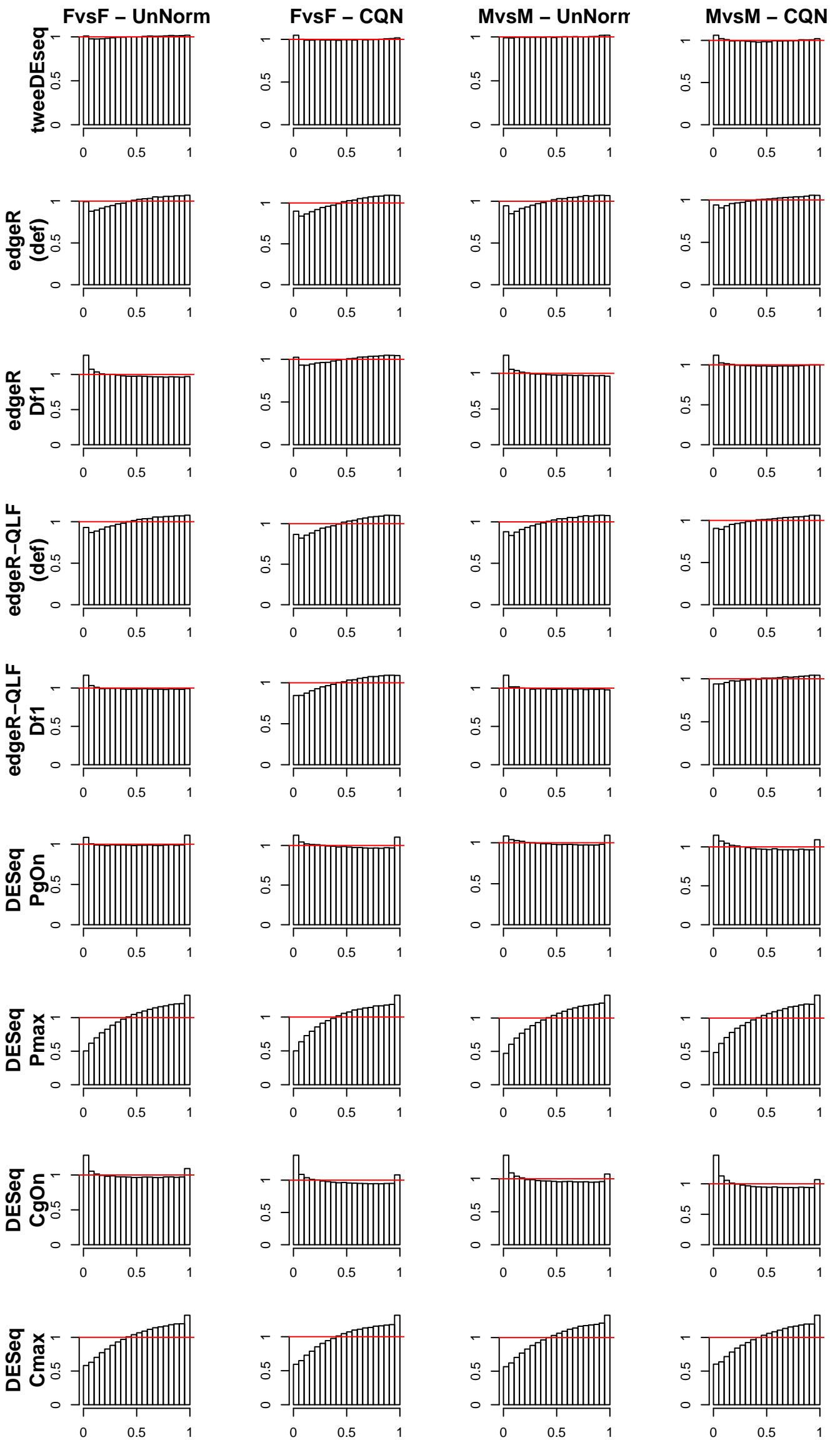

Supplement: Additional file 1 — Scripts. ZIP file (.zip) containing all scripts, in the form of Sweave vignettes, to reproduce the results shown in this paper, including one copy of the resulting PDF file. Please read first through the README file contained in this tar ball in order to understand how to run the scripts. [file 1471-2105-14-254-S1.zip › tweeDEseqSupportingAnalyses/pickrell1simulations/histPickrell1sims.pdf]

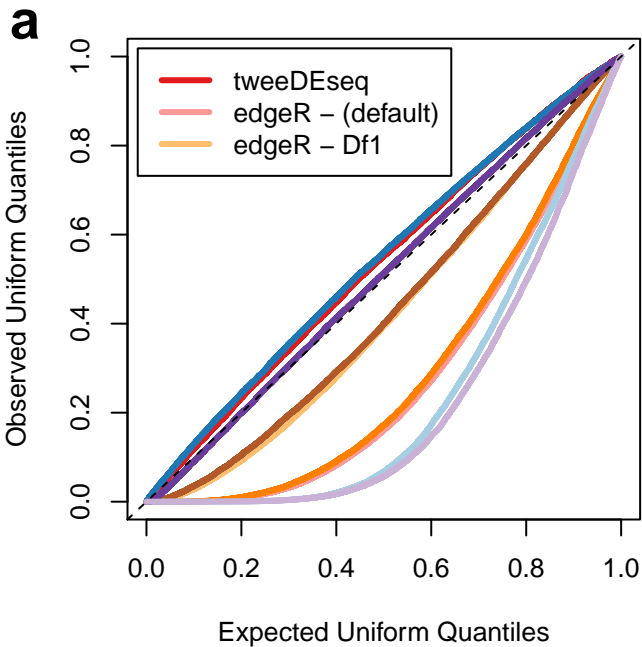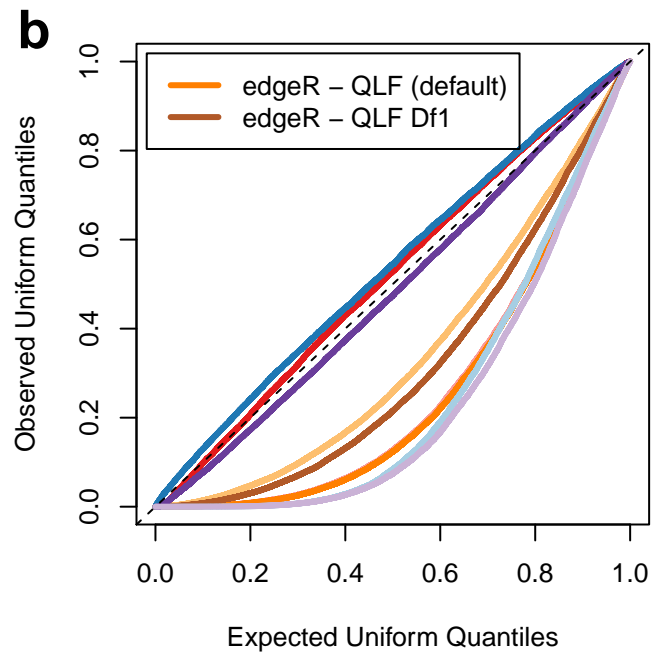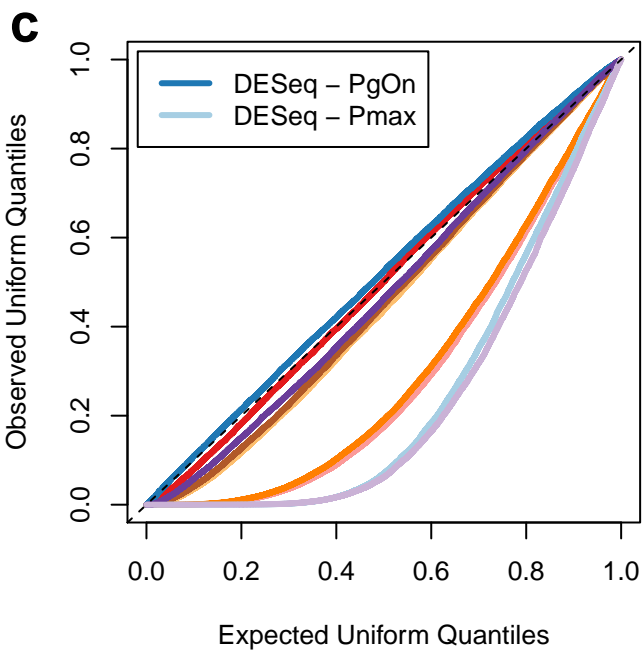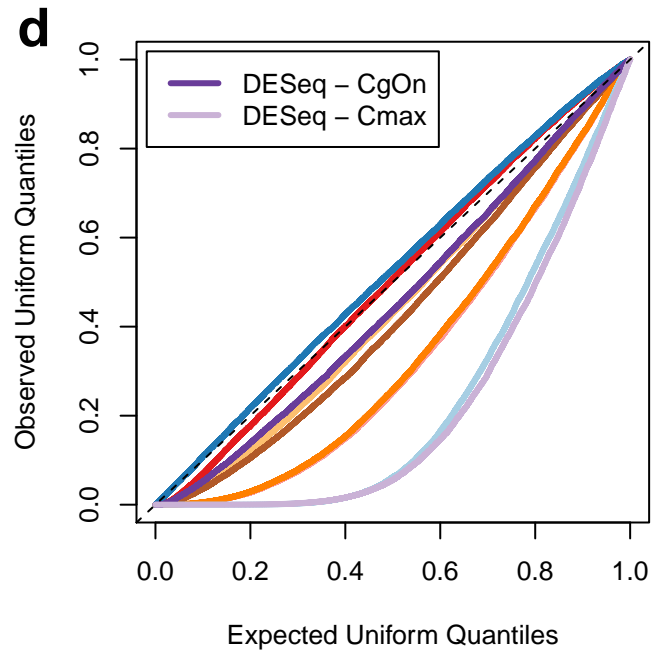

Supplement: Additional file 1 — Scripts. ZIP file (.zip) containing all scripts, in the form of Sweave vignettes, to reproduce the results shown in this paper, including one copy of the resulting PDF file. Please read first through the README file contained in this tar ball in order to understand how to run the scripts. [file 1471-2105-14-254-S1.zip › tweeDEseqSupportingAnalyses/pickrell1simulations/qqPickrell1sims.pdf]
