# Supplementary material for: A flexible count data model to fit the wide diversity of expression profiles arising from extensively replicated RNA-seq experiments
Source: BMC Bioinformatics. 2013 Aug 21;14:254. doi: 10.1186/1471-2105-14-254 (PMC3849762; doi:10.1186/1471-2105-14-254)
Supplement: Additional file 2 — Supplementary materials. PDF file including supplementary figures and tables. [file 1471-2105-14-254-S2.pdf]

Supplementary information

A flexible count data model to fit the wide diversity of expression profiles arising from extensively replicated RNA-seq experiments

Mikel Esnaola[1], Pedro Puig[2], David Gonzalez[3], Robert Castelo[4,5,*] and Juan R Gonzalez[1,2,5,6,*]

1 Center for Research in Environmental Epidemiology (CREAL), Barcelona, Spain

2 Department of Mathematics, Universitat Autònoma de Barcelona (UAB), Barcelona, Spain

3 Center for Genomic Regulation (CRG), Barcelona, Spain

4 Department of Experimental and Health Sciences, Research Program on Biomedical Informatics (GRIB), Universitat Pompeu Fabra, Barcelona, Spain

5 Hospital del Mar Research Institute (IMIM), Barcelona, Spain

6 CIBER Epidemiology and Public Health (CIBERESP), Spain

$*$ Corresponding authors: Juan R Gonzalez (`jrgonzalez@creal.cat`) and R Castelo (`robert.castelo@upf.edu`)

Simulation Studies

Control of the Type-I error rate

In order to compare assess the control of the Type-I error rate among different methods we have chosen the way in which the results are presented in Anders and Huber (2010)[1]. We plot the empirical cumulative distribution function (ECDF) of the P value for each method. By doing so, we can perform direct comparisons between our method and previous approaches. The purpose of these simulation studies is to verify that `tweeDEseq` controls the type-I error at the nominal significance level. Therefore, the proportion of P values below a threshold α must be $\leq \alpha$, that is, the ECDF curve should not lie above the diagonal.

RNA-seq count data were simulated as suggested in Robinson and Smyth (2008)[2]. The library sizes are sampled from a uniform distribution between 30,000 and 90,000. These library sizes are considerably smaller than those available from the current sequencing technologies and can be even more different from those used in the near future. However, increasing the library size to better reflect actual data does not alter the conclusions obtained, because the library size acts, in fact, as a scaling factor. We simulated 20,000 genes, each of them from a PT distribution varying the a parameter to mimic real situations as the ones illustrated in Figure 3 of the main manuscript. In particular, we simulated RNA-seq counts following a PT distribution according to four

scenarios that cover a range of PT models with different parameters a as indicated in Table S2. We simulated data sets for 25 and 50 individuals per group. Results of these simulations can be found in Figures S3 to S7.

Required sample size to control the Type-I error rate

We also performed a simulation study to estimate the number of samples required by our approach. We simulated data under the four scenarios described in Table S2. For each of them we simulated 200 replicates varying the sample size per group in $\{3, 6, 9, 12, 15, 18, 20, 25, 30, 35, 40\}$. Results of these simulations can be found in Figure S7. We can observe that as the sample size increases the empirical Type-I error attains its nominal level in each of the four scenarios. From this simulation, it follows that with a minimum sample size of $n = 15$ a significance level $\alpha = 0.05$ is properly controlled.

Supplementary Tables

Supplementary Table S1: Enrichment of non-NB genes among housekeeping genes through the data of Pickrell *et al.* (2010) processed with different normalization methods.

	HKG	nNByes	nNBno	Total	OR	Pvalue
**UnNorm**	Yes	31	546	577		
	No	187	26355	26542		
	Total	218	26901	27119	8.0	6.56e-17
	HKG	nNByes	nNBno	Total	OR	Pvalue
**edgeR**	Yes	19	558	577		
	No	36	27637	27673		
	Total	55	28195	28250	26.1	8.27e-19
	HKG	nNByes	nNBno	Total	OR	Pvalue
**cqn**	Yes	32	545	577		
	No	139	23265	23404		
	Total	171	23810	23981	9.8	1.49e-19

Supplementary Table S2: Description of four different scenarios for a simulation study assessing the type-I error rate in two-sample tests involving sample groups from different count data distributions.

	Group A	Group B	Distributions
Scenario 1	$PT(\mu = 1, \phi = 30, a = -140)$	$PT(\mu = 1, \phi = 30, a = 0.5)$	Neyman Type I vs PIG
Scenario 2	$PT(\mu = 20, \phi = 100, a = 0.5)$	$PT(\mu = 20, \phi = 200, a = -1)$	PIG vs Pòlya-Aeppli
Scenario 3	$PT(\mu = 200, \phi = 150, a = -1)$	$PT(\mu = 200, \phi = 200, a = 0)$	Pòlya-Aeppli vs NB
Scenario 4	$PT(\mu = 3, \phi = 20, a = 0)$	$PT(\mu = 3, \phi = 20, a = 0)$	NB vs NB

Supplementary Table S3: Differentially expressed genes between female and male Nigerian individuals called by tweeDEseq at 10% FDR. Genes are ordered by their absolute fold-change in $\log_2$ scale. The "Gender specific" column indicates those genes reported in the literature as belonging to the male-specific region of the Y chromosome (MSY[3]) or escaping to the inactivation of the Xi chromosome (XiE[4]).

#	Ensembl Gene Identifier	Gene Symbol	Chr	Description	Log_2 FC	Gender Specific
1	ENSG00000229807	XIST	X	X (inactive)-specific transcript (non-protein coding)	8.39	XiE

Supplementary Table S3 – Continued on next page

Supplementary Table S3 – continued from previous page

2	ENSG00000131002	CYorf15B	Y	chromosome Y open reading frame 15B	-4.44	MSY
3	ENSG00000165246	NLGN4Y	Y	neuroligin 4, Y-linked	-3.90	MSY
4	ENSG00000099749	CYorf15A	Y	chromosome Y open reading frame 15A	-3.87	MSY
5	ENSG00000213318	RP11-331F4.1	16		-3.60	
6	ENSG00000233864	TTTY15	Y	testis-specific transcript, Y-linked 15 (non-protein coding)	-3.54	
7	ENSG00000157828	RPS4Y2	Y	ribosomal protein S4, Y-linked 2	-3.18	MSY
8	ENSG00000230986	RP13-204A15.1	X		-3.05	
9	ENSG00000146938	NLGN4X	X	neuroligin 4, X-linked	-3.04	XiE
10	ENSG00000129824	RPS4Y1	Y	ribosomal protein S4, Y-linked 1	-2.55	MSY
11	ENSG00000243209	AC010889.1	Y		-2.43	
12	ENSG00000198692	EIF1AY	Y	eukaryotic translation initiation factor 1A, Y-linked	-2.21	MSY
13	ENSG00000011201	KAL1	X	Kallmann syndrome 1 sequence	-1.81	
14	ENSG00000183878	UTY	Y	ubiquitously transcribed tetratricopeptide repeat gene, Y-linked	-1.75	MSY
15	ENSG00000241859	KALP	Y	Kallmann syndrome sequence pseudogene	-1.64	

Supplementary Table S3 – Continued on next page

Supplementary Table S3 – continued from previous page

16	ENSG00000067048	DDX3Y	Y	DEAD (Asp-Glu-Ala-Asp) box polypeptide 3, Y-linked	-1.59	MSY
17	ENSG00000232928	RP13-204A15.4	X		-1.42	
18	ENSG00000231535	NCRNA00278	Y	non-protein coding RNA 278	-1.38	
19	ENSG00000012817	KDM5D	Y	lysine (K)-specific demethylase 5D	-1.37	
20	ENSG00000006757	PNPLA4	X	patatin-like phospholipase domain containing 4	1.01	XiE
21	ENSG00000005302	MSL3	X	male-specific lethal 3 homolog (Drosophila)	0.91	
22	ENSG00000101846	STS	X	steroid sulfatase (microsomal), isozyme S	0.89	XiE
23	ENSG00000215520	RP11-401M16.3	1		0.83	
24	ENSG00000239254	RP11-365F18.3	7		0.83	
25	ENSG00000130021	HDHD1	X	haloacid dehalogenase-like hydrolase domain containing 1	0.82	XiE
26	ENSG00000224287	MSL3P1	2	male-specific lethal 3 homolog (Drosophila) pseudogene 1	0.81	
27	ENSG00000198034	RPS4X	X	ribosomal protein S4, X-linked	0.80	XiE
28	ENSG00000239490	RP11-863N1.1	18		0.80	
29	ENSG00000229920	AC016734.3	2		0.80	

Supplementary Table S3 – Continued on next page

Supplementary Table S3 – continued from previous page

30	ENSG00000214541	AL137162.1	20		0.80	
31	ENSG00000242058	RP11-143J12.1	18		0.77	
32	ENSG00000186008	RPS4XP21	19	ribosomal protein S4X pseudogene 21	0.76	
33	ENSG00000162639	HENMT1	1	HEN1 methyltransferase homolog 1 (Arabidopsis)	-0.76	
34	ENSG00000239830	CTD-3116E22.2	19		0.75	
35	ENSG00000243663	RP11-21K20.1	12		0.74	
36	ENSG00000219146	RP11-134L4.1	6		0.73	
37	ENSG00000226948	RP5-1068H6.3	20		0.72	
38	ENSG00000224892	RPS4XP16	13	ribosomal protein S4X pseudogene 16	0.72	
39	ENSG00000244097	RP11-411G7.1	17		0.72	
40	ENSG00000240371	RP11-624G17.1	11		0.71	
41	ENSG00000214203	RP11-135F9.1	12		0.71	
42	ENSG00000218265	RP11-501I19.4	6		0.71	
43	ENSG00000244073	CTD-2284O10.1	5		0.70	
44	ENSG00000234335	RP11-241I20.3	10		0.69	
45	ENSG00000240721	RPS4XP15	12	ribosomal protein S4X pseudogene 15	0.68	
46	ENSG00000205664	RP11-706O15.1	X	HCG1981372, isoform CRA_cNovel protein	0.65	
47	ENSG00000126012	KDM5C	X	lysine (K)-specific demethylase 5C	0.62	XiE
48	ENSG00000229305	RP11-431K24.2	1		0.57	
49	ENSG00000173674	EIF1AX	X	eukaryotic translation initiation factor 1A, X-linked	0.56	XiE

Supplementary Table S3 – Continued on next page

Supplementary Table S3 – continued from previous page

50	ENSG00000114374	USP9Y	Y	ubiquitin specific peptidase 9, Y-linked	-0.51	MSY
51	ENSG00000005889	ZFX	X	zinc finger protein, X-linked	0.49	XiE
52	ENSG00000086712	TXLNG	X	taxilin gamma	0.47	
53	ENSG00000215301	DDX3X	X	DEAD (Asp-Glu-Ala-Asp) box polypeptide 3, X-linked	0.46	XiE
54	ENSG00000067646	ZFY	Y	zinc finger protein, Y-linked	-0.45	MSY
55	ENSG00000147050	KDM6A	X	lysine (K)-specific demethylase 6A	0.43	

Supplementary Figures

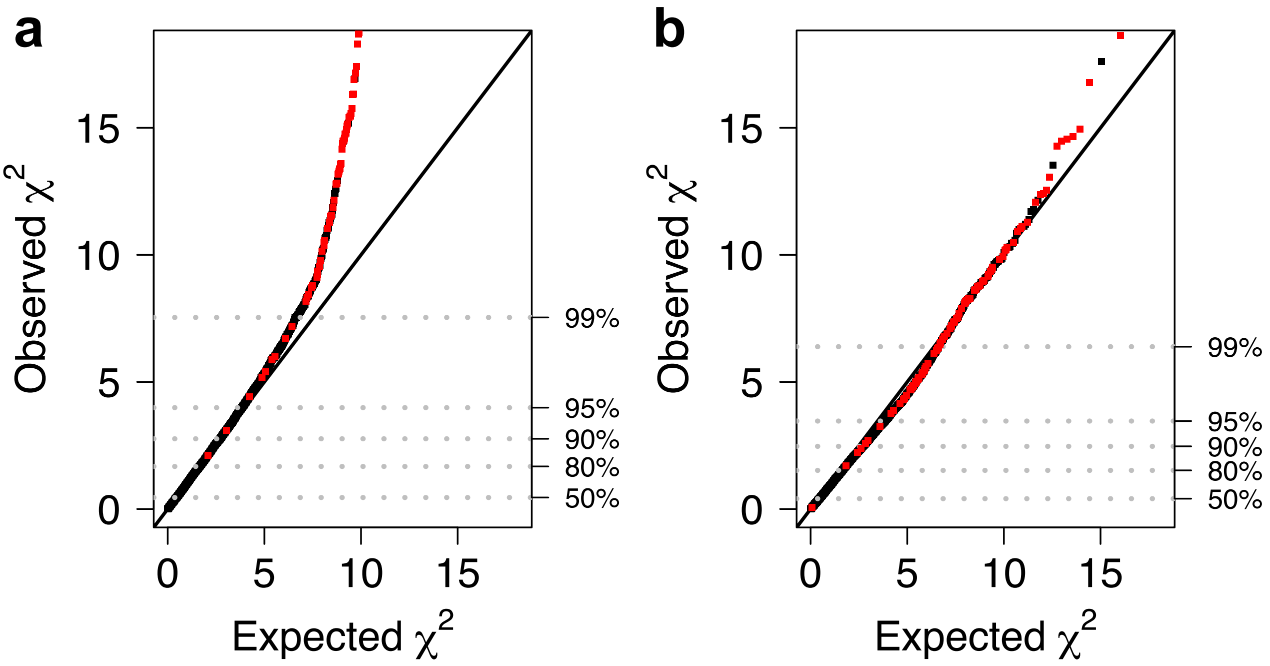

Supplementary Figure S1: Goodness of fit of negative-binomial simulated data to the negative-binomial distribution. Quantile-quantile plots of the χ^2 statistic employed to assess the goodness-of-fit to a negative binomial distribution. The right y-axis indicates the quantile of the observed distribution. (a) Synthetic counts for 23,971 genes and $n = 1,000$ samples simulated from a negative binomial distribution with mean count 32 and dispersion parameter 0.2. Red points correspond to 100 genes which in 42% of the samples had mean count 64, thus simulating them as differentially expressed. (b) Same as (a) but with $n = 69$ samples, thereby reproducing dimensions similar to those of the Pickrell[5] *et al.* (2010) data.

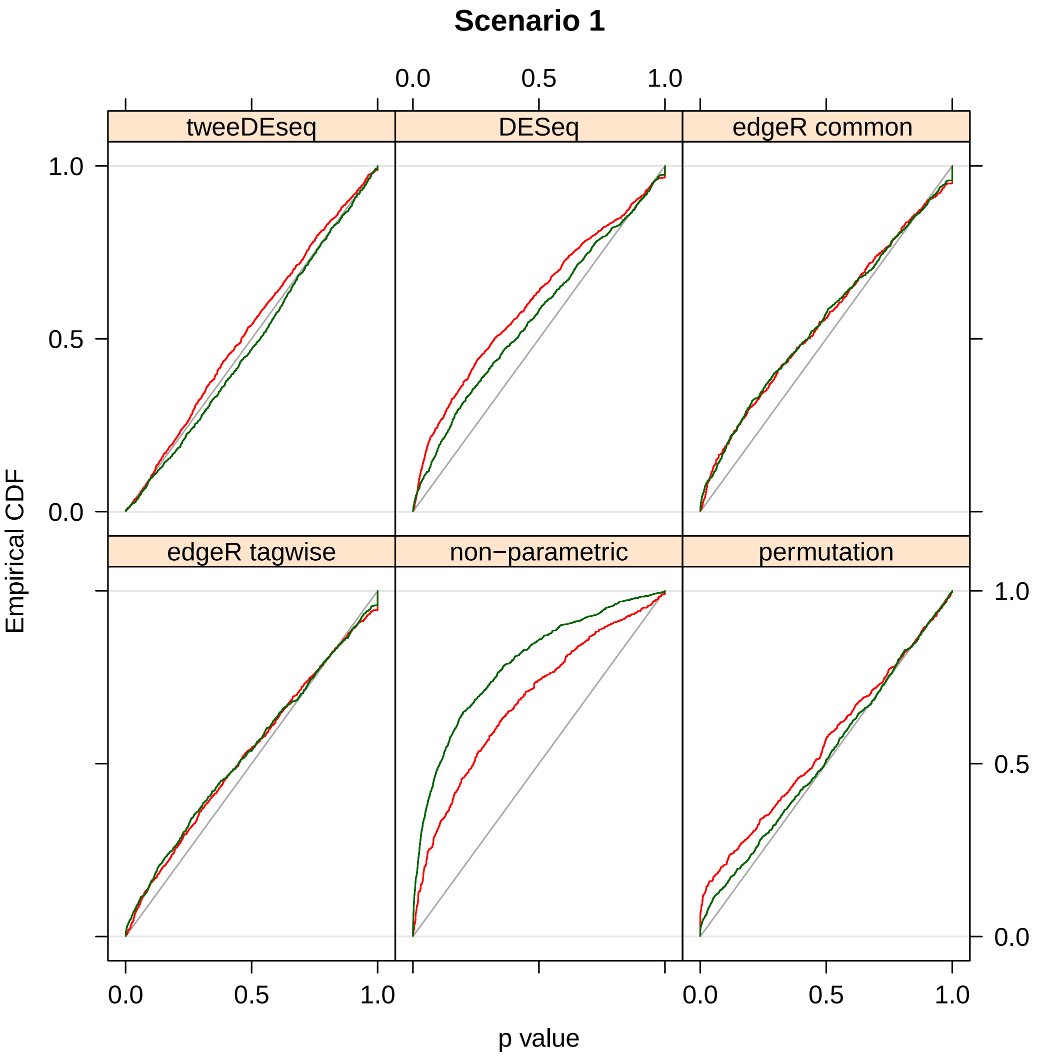

Supplementary Figure S2: Control of the Type-I error under scenario 1 in Table S2. Each panel shows empirical cumulative distribution functions (ECDFs) for p-values when comparing two groups. No genes are truly differentially expressed. Therefore, ECDF curves should remain below the diagonal (gray). Each panel gives the results for different method and they illustrate the performance when simulating data from a Poisson-Tweedie distribution with parameter $a = -140$ and $a = 0.5$ for each group, respectively. Therefore, counts belonging to Group A follow a Neyman Type A distribution, while counts for group B can be modeled using a Poisson Inverse Gaussian. Results for different samples sizes are shown (n=50 green line, n=25 red line). We can observe as `tweeDEseq` controls for type-I error (specially for n=50), while the other methods does not. This means that other approaches can potentially lead to a large number of false positive findings.

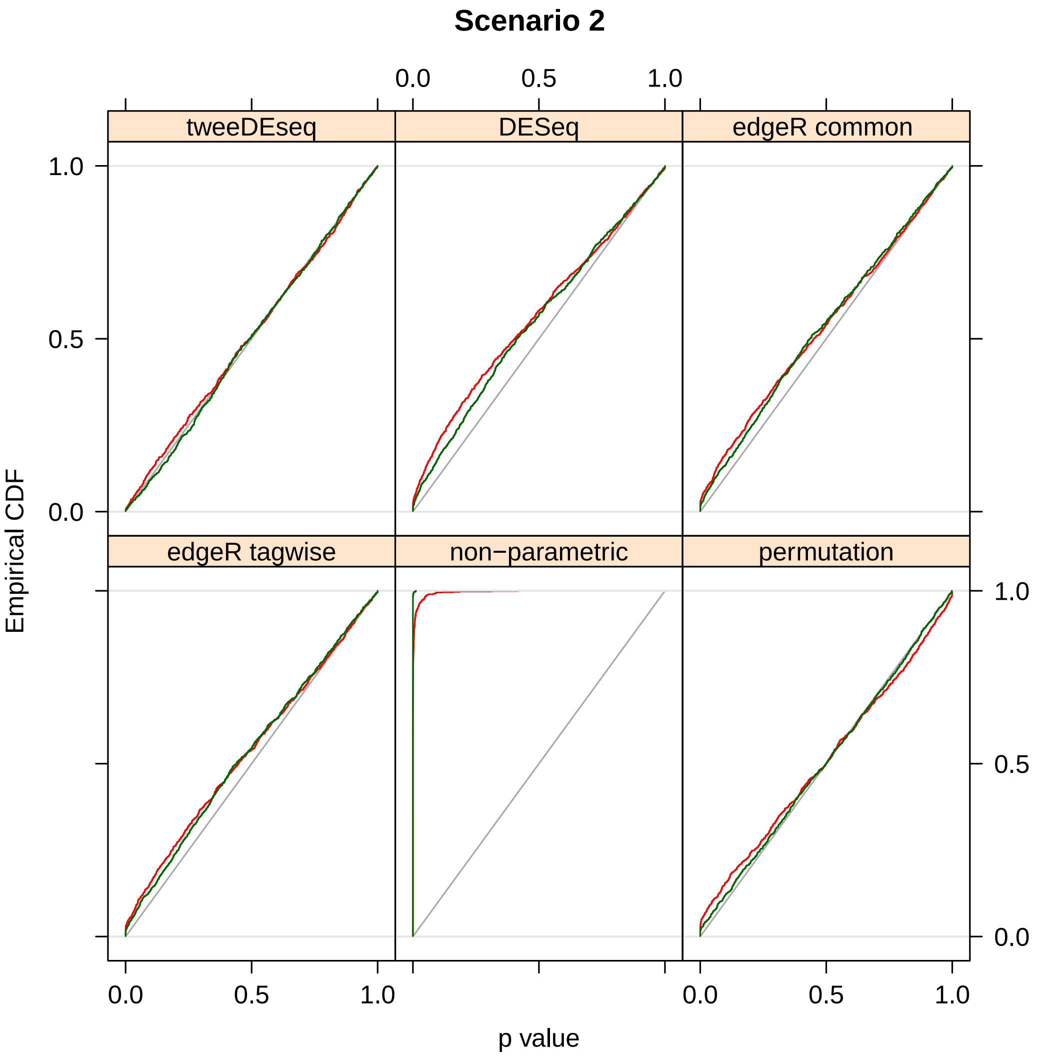

Supplementary Figure S3: **Control of the Type-I error under scenario 2 in Table S2.** Each panel shows empirical cumulative distribution functions (ECDFs) for p-values when comparing two groups. No genes are truly differentially expressed. Therefore, ECDF curves should remain below the diagonal (gray). Each panel gives the results for different method and they illustrate the performance when simulating data from a Poisson-Tweedie distribution with parameter $a = 0.5$ and $a = -1$ for each group, respectively. Therefore, counts belonging to Group A follow a Poisson Inverse Gaussian distribution, while counts for group B can be modeled using a Pòlya-Aeppli model. Results for different samples sizes are shown (n=50 green line, n=25 red line). We can observe as `tweeDEseq` controls for type-I error (specially for n=50), while the other methods (except `edgeR` with tagwise dispersion mode) does not. This means that other approaches can potentially lead to a large number of false positive findings.

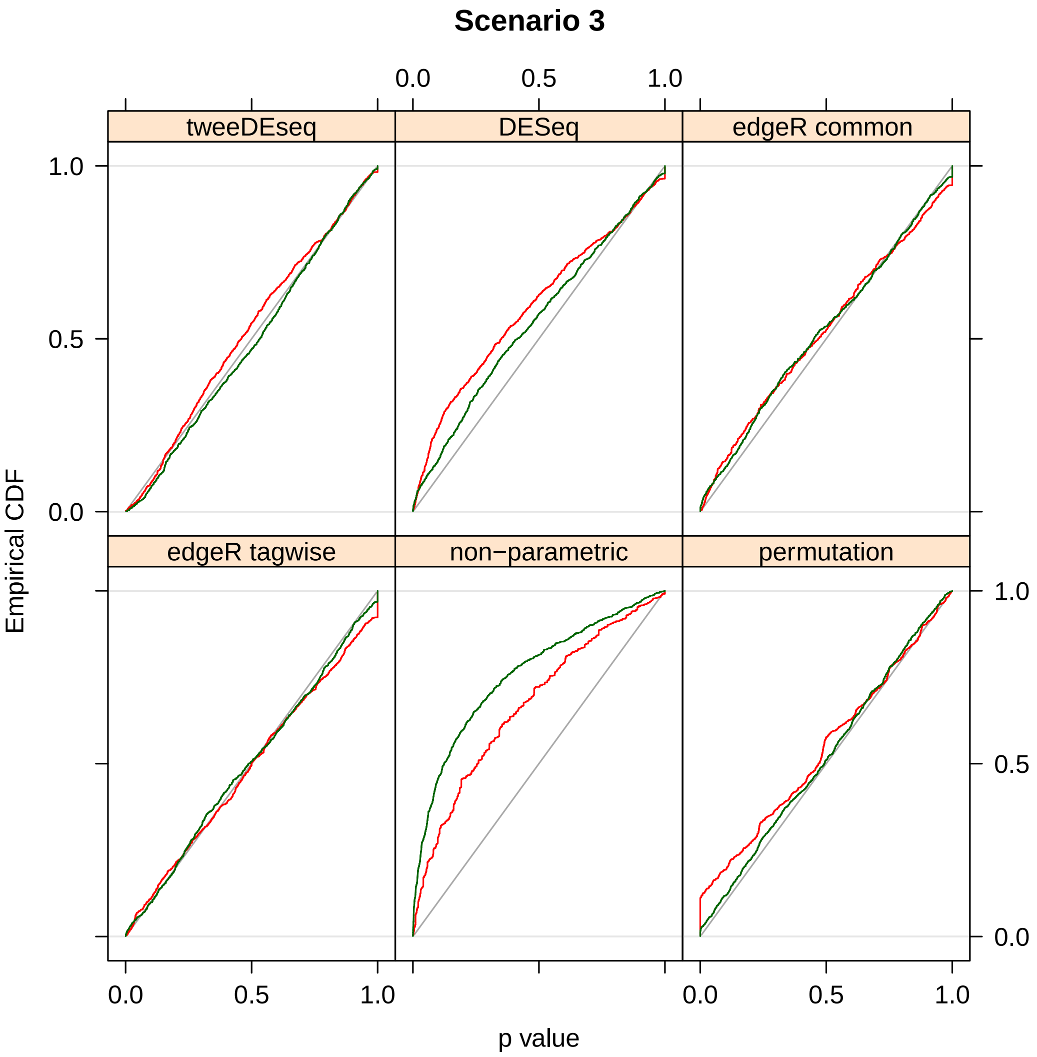

Supplementary Figure S4: **Control of the Type-I error under scenario 3 in Table S2.** Each panel shows empirical cumulative distribution functions (ECDFs) for p-values when comparing two groups. No genes are truly differentially expressed. Therefore, ECDF curves should remain below the diagonal (gray). Each panel gives the results for different method and they illustrate the performance when simulating data from a Poisson-Tweedie distribution with parameter $a = -1$ and $a = 0$ for each group, respectively. Therefore, counts belonging to Group A follow a Pòlya-Aeppli distribution, while counts for group B can be modeled using a NB. Results for different samples sizes are shown (n=50 green line, n=25 red line). We can observe as `tweeDEseq` controls for type-I error (specially for n=50), while the other methods (except `edgeR` with tagwise dispersion mode) does not. This means that other approaches can potentially lead to a large number of false positive findings.

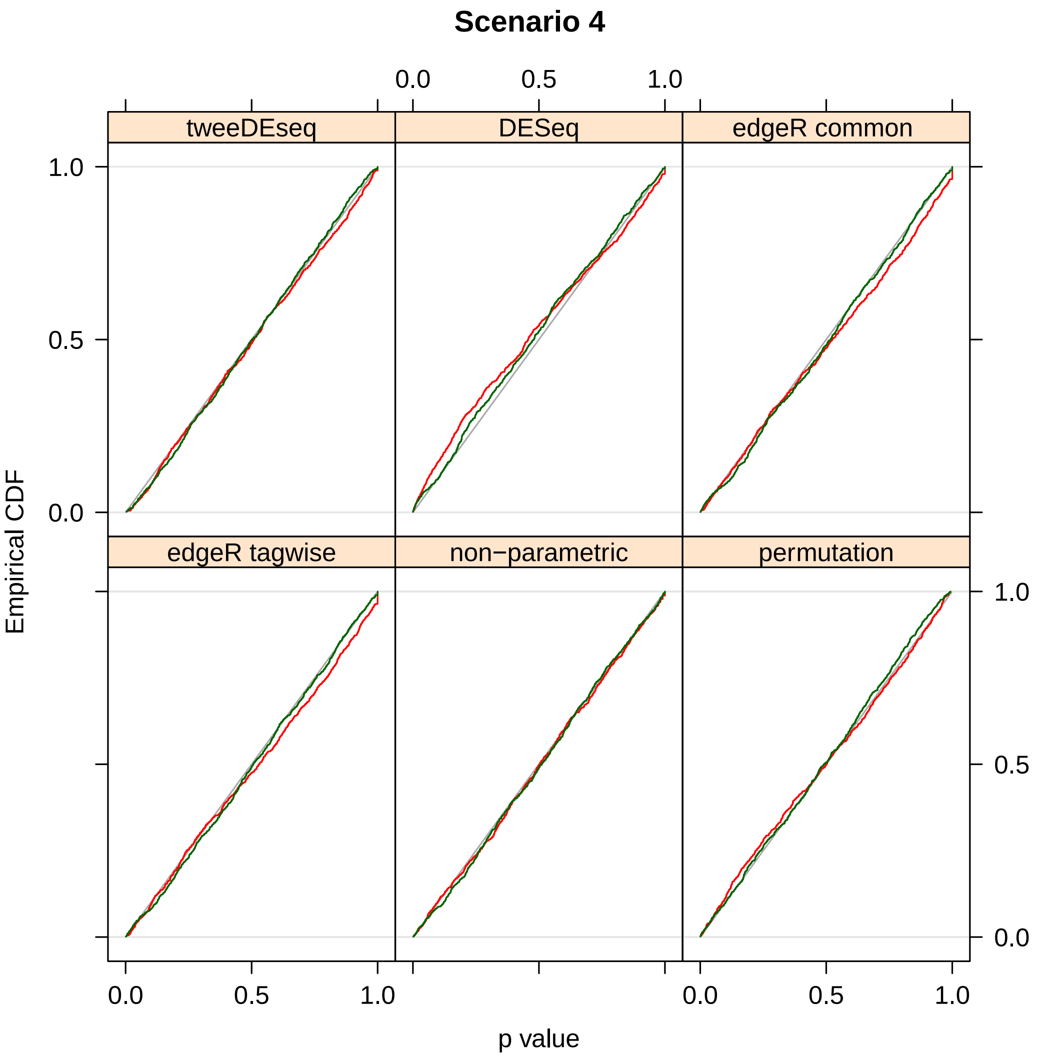

Supplementary Figure S5: Control of the Type-I error under scenario 4 in Table S2. Each panel shows empirical cumulative distribution functions (ECDFs) for p-values when comparing two groups. No genes are truly differentially expressed. Therefore, ECDF curves should remain below the diagonal (gray). Each panel gives the results for different method and they illustrate the performance when simulating data from a Poisson-Tweedie distribution with parameter $a = 0$ and $a = 0$ for each group, respectively. Therefore, counts for each groups are simulated using a Negative Binomial distribution. Results for different samples sizes are shown (n=50 green line, n=25 red line). In this case, as all methods are based on Negative Binomial distribution, hence, the results are quite almost identical.

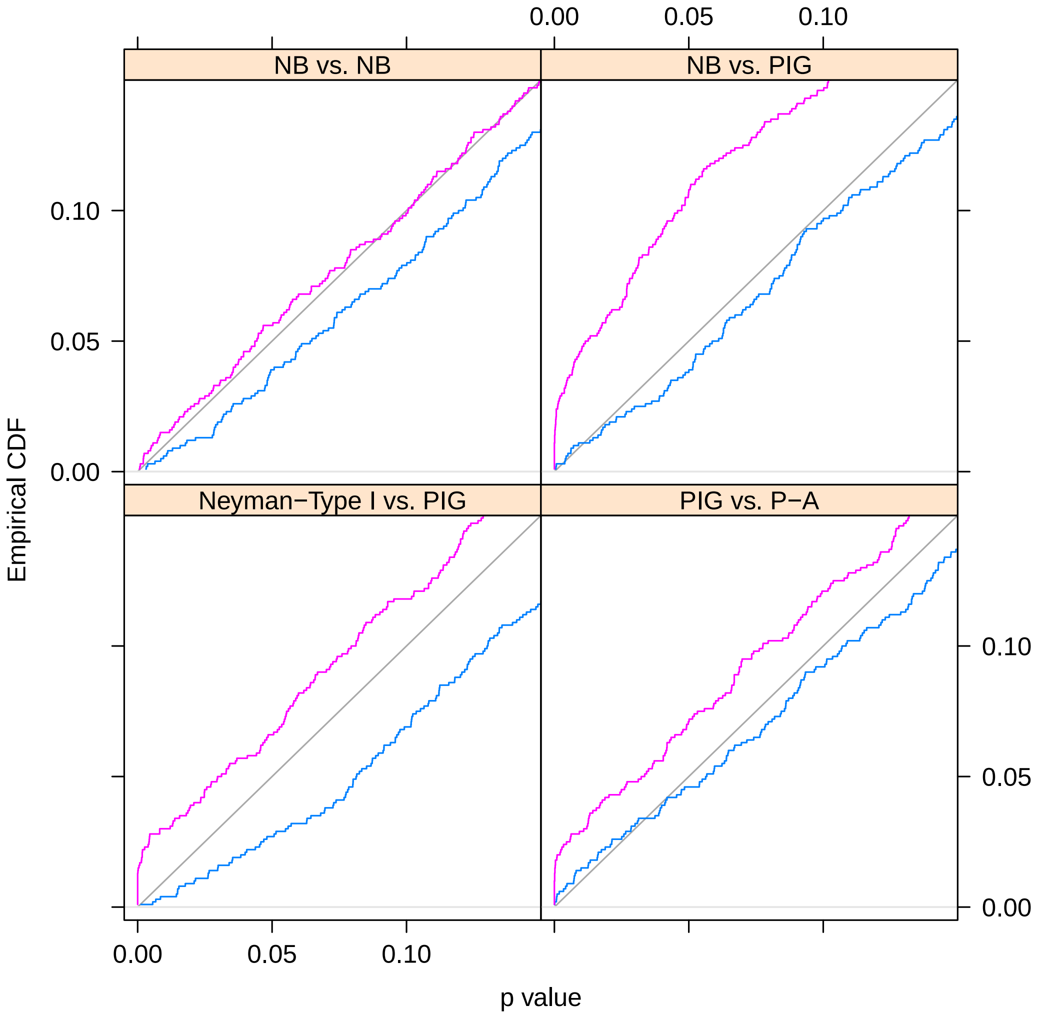

Supplementary Figure S6: **Type I error control in the tail of the distribution.** Each panel shows empirical cumulative distribution functions (ECDFs) for p-values when comparing two groups using `tweeDEseq` (blue line) and permutation testing (pink line) approaches. Results zoom into the range of small p-values. No genes are truly differentially expressed. Therefore, ECDF curves should remain below the diagonal (gray). Each panel gives the results for the Scenarios described in Supplementary Table 1. We can observe how permutation testing does not control the type I error rate, while `tweeDEseq` does.

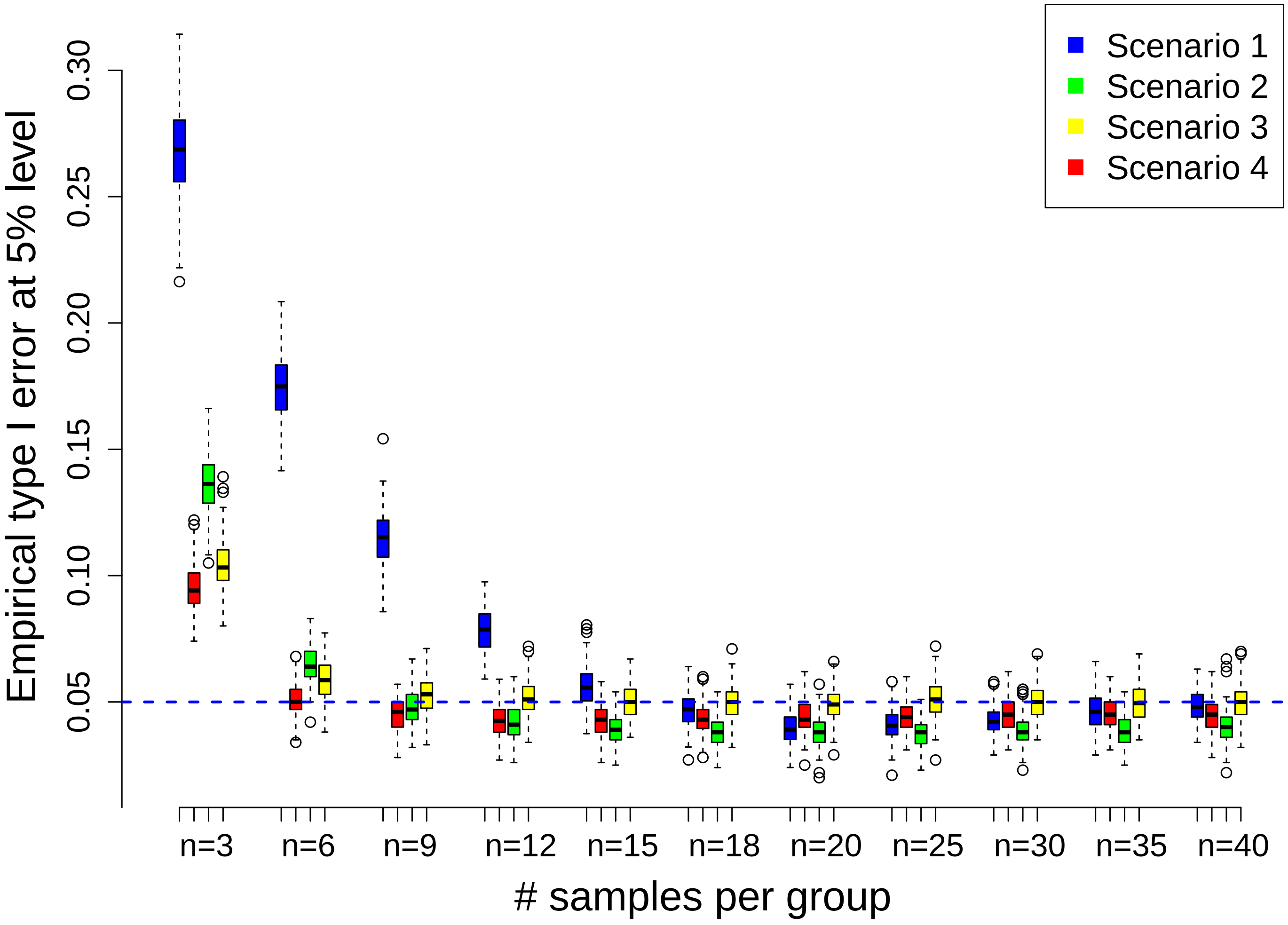

Supplementary Figure S7: Optimal sample size to use `tweeDEseq`. This figure shows the empirical Type-I error on the y-axis as function of the sample size and scenario described in Table S2. The nominal Type-I error rate ($\alpha = 0.05$) is indicated by an horizontal dash line. We simulated data under the null hypothesis that no genes are differentially expressed. Ideally, each boxplot should be centered over the dash line which represents the expected Type-I error at that level.

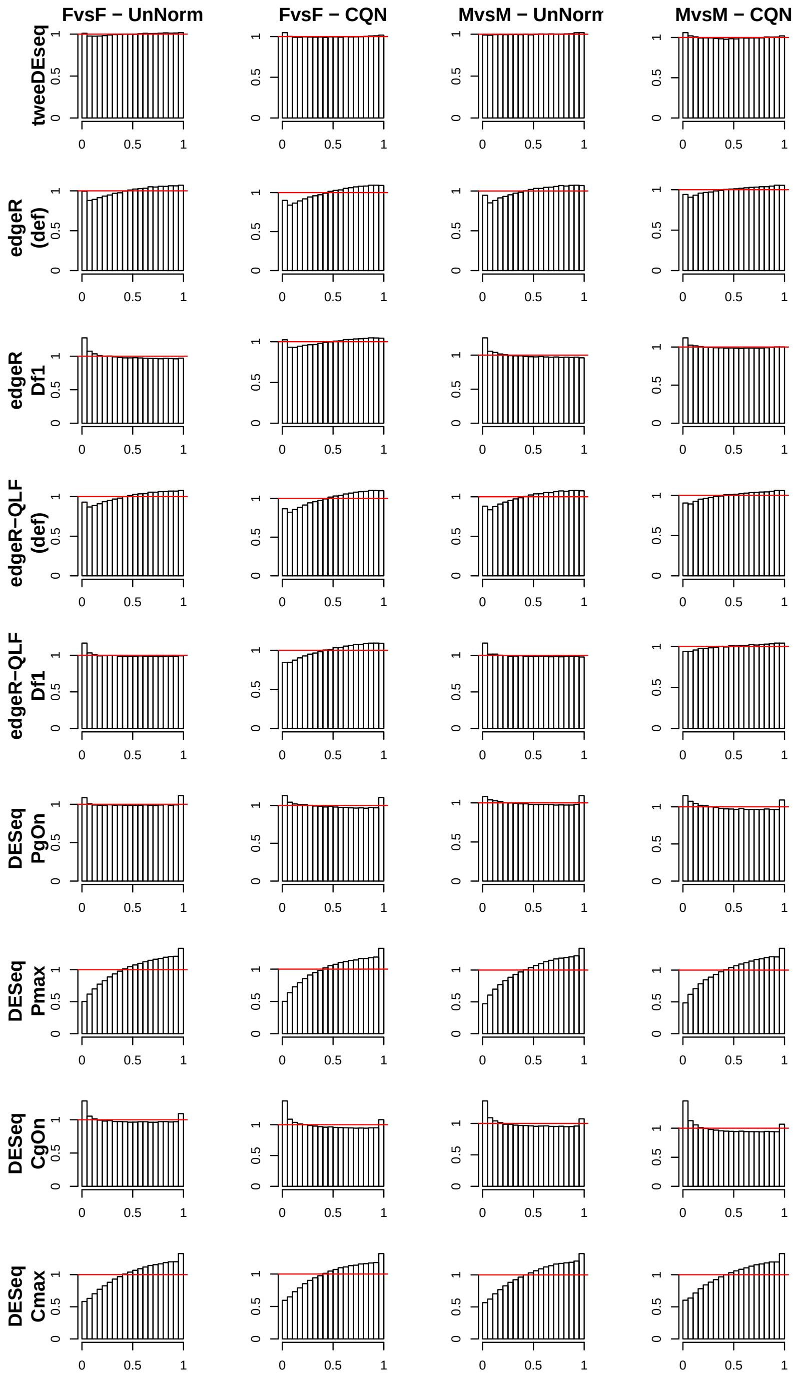

Supplementary Figure S8: Distribution of p-values under the null hypothesis of no differential expression. Histograms indicating the density (y-axis) of raw p-values (x-axis) obtained by every method and parameter configuration indicated on the left edge on 100 data sets bootstrapped separately from male and female samples of the LCL RNA-seq data[5]. An horizontal red line at density one indicates the uniform distribution. Each data set contained 40 female (columns 1 and 2) and male (columns 3 and 4) samples, arbitrarily divided into two equally-sized groups where the two-sample test of each corresponding method was applied. Results in columns 1 and 3 were obtained from the raw un-normalized counts and in columns 2 and 4 from counts normalized with the `cqn` package[6]. No differential expression is expected and thus p-values should be uniformly distributed. In the first row `tweeDEseq` displays the distributions closest to the red line and hence to this criterion.

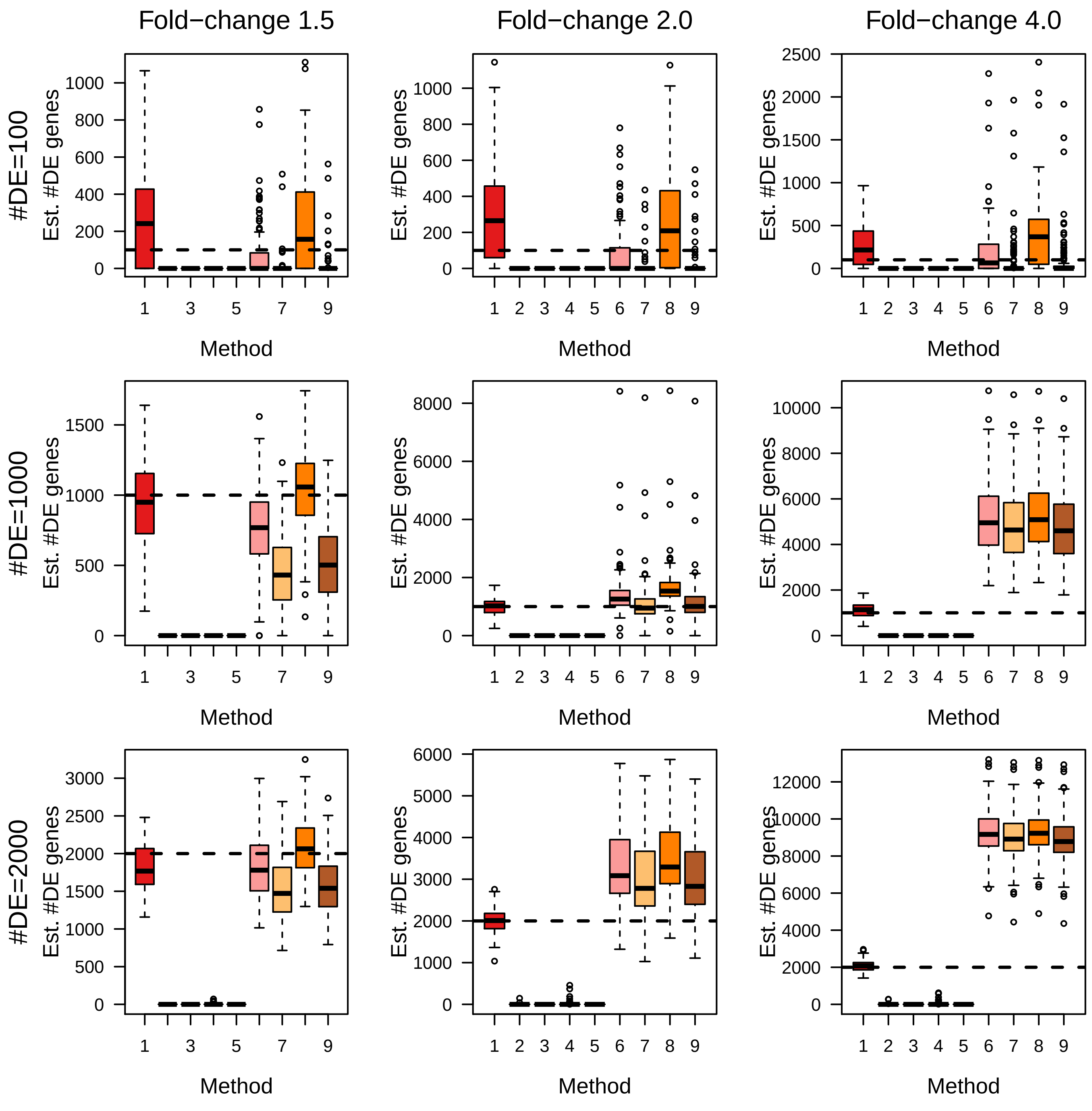

Supplementary Figure S9: Estimation of the number of differentially expressed (DE) genes in simulations with constant library factors. Boxplots of estimated numbers of DE genes obtained from the estimate $\hat{\pi}_0$ of genes that are truly non-DE calculated using the package `qvalue`[7]. Each panel corresponds to simulations using a different number of DE genes (rows) and their fold-change (columns). All simulations draw data from a hierarchical gamma-Poisson model with constant library factors. Horizontal dash lines indicate the true number of DE genes.

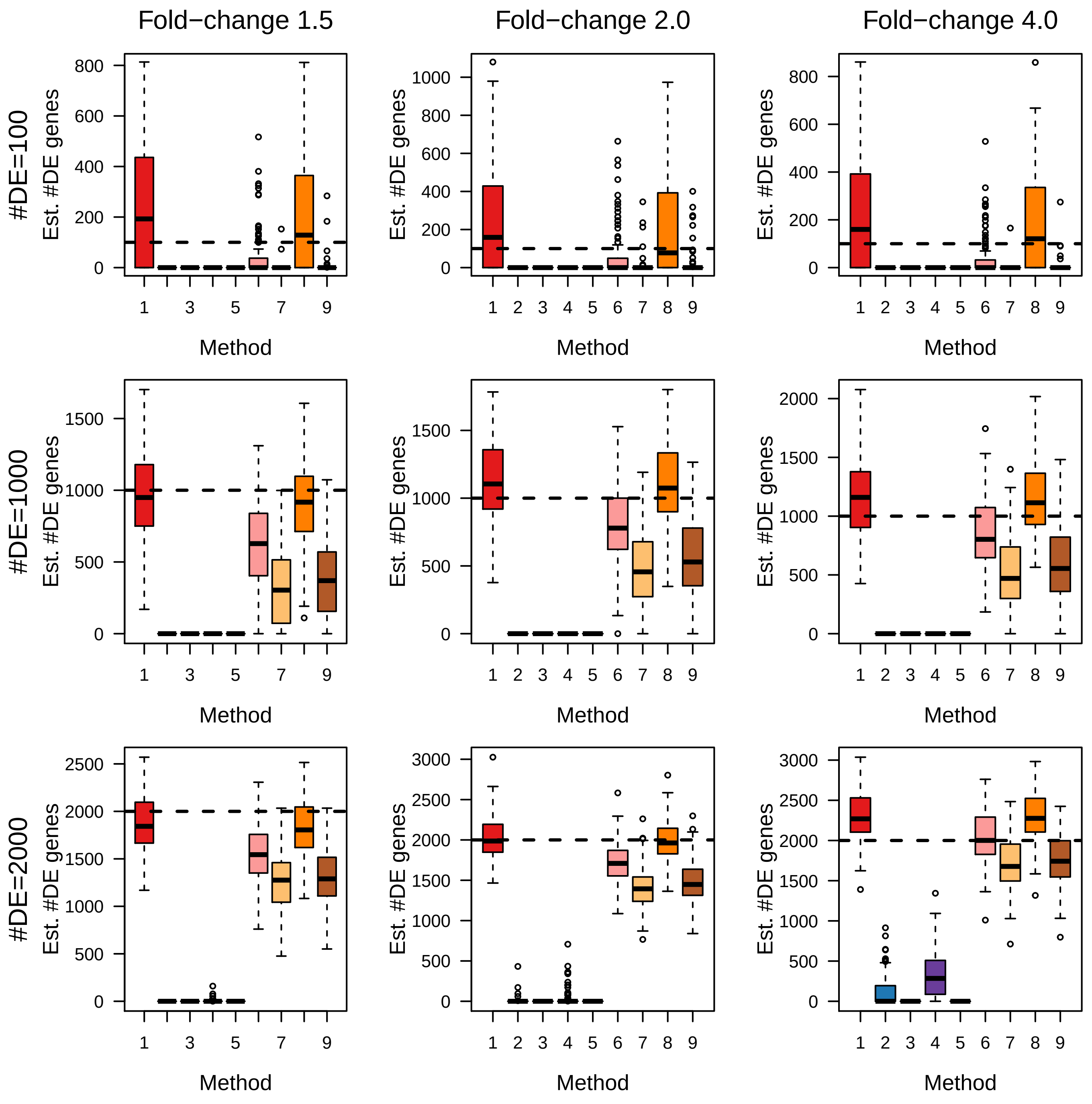

Supplementary Figure S10: Estimation of the number of differentially expressed (DE) genes in simulations with variable library factors. Boxplots of estimated numbers of DE genes obtained from the estimate $\hat{\pi}_0$ of genes that are truly non-DE calculated using the package `qvalue`[7]. Each panel corresponds to simulations using a different number of DE genes (rows) and their fold-change (columns). All simulations draw data from a hierarchical gamma-Poisson model with variable library factors. Horizontal dash lines indicate the true number of DE genes.

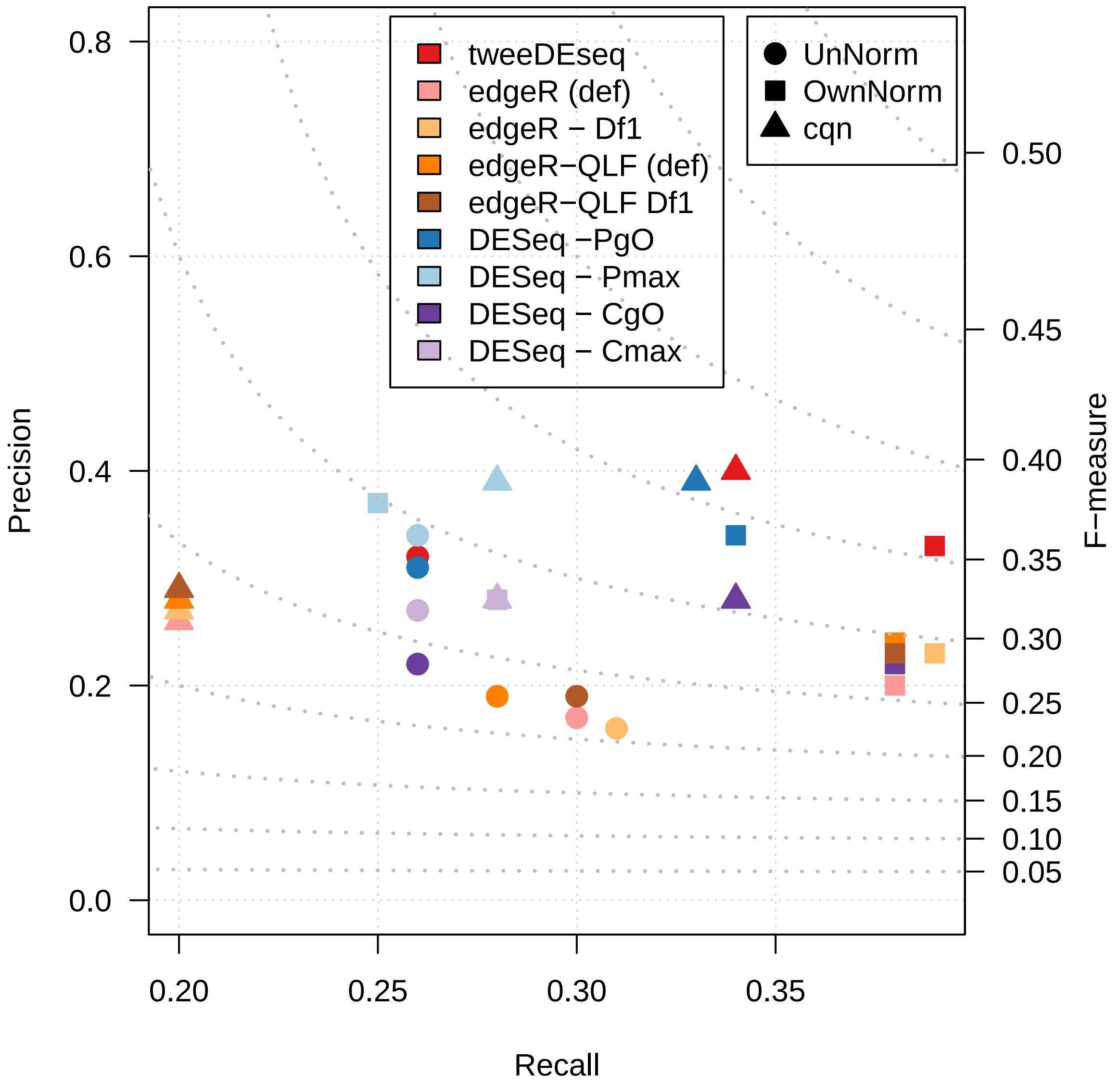

Supplementary Figure S11: **Precision and recall comparison on the LCL RNA-seq data.** Precision (y-axis) and recall (x-axis) values for genes called DE at 10% FDR by different DE detection methods and configuration parameters. The right y-axis indicates values of the F-measure shown by dot lines. As the figure shows, on the normalized data `tweeDEseq` provides higher F-measure values than other methods and configuration parameters indicating a better precision-recall tradeoff. These results were obtained by applying a more stringent filter on lowly expressed genes than the one shown in Figure 10 of the main article.

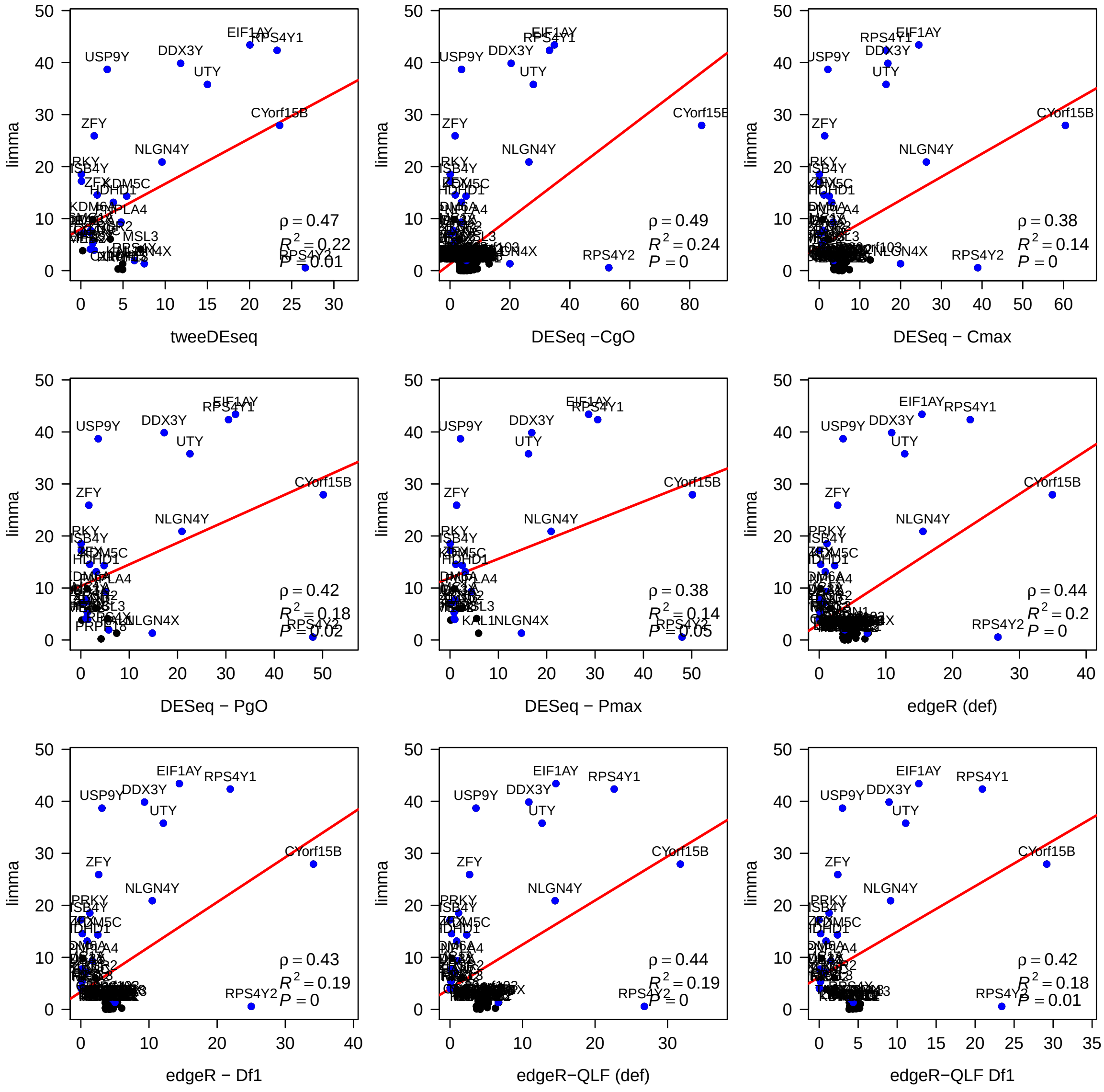

Supplementary Figure S12: Reproducibility of differential expression (DE) between microarray and RNA-seq. Raw p-values of differential expression in $-\log_{10}$ scale for DE genes called at 10% FDR by either limma (y-axis), from microarray data, or the other compared DE detection method applied on RNA-seq data (x-axis). A regression line is depicted in red and blue points denote genes with documented sex-specific expression. On the bottom-right corner of each panel, ρ indicates the Pearson correlation whereas R^2 and P indicate, respectively, the coefficient of determination and p-value of the test for zero regression coefficient, of the $-\log_{10} p$-values of limma as function of those from the compared RNA-seq method. Even though the relationships are significant in all comparisons, the low R^2 values indicate a poor level of reproducibility between microarray and RNA-seq DE analysis, irrespectively of the method employed for detecting DE genes in RNA-seq data. A large fraction of irreproducible DE is due to genes that are called DE by one technology but not by the other. Blue dots indicate genes with documented sex-specific expression.

References

[1] Anders S, Huber W: **Differential expression analysis for sequence count data**. *Genome Biol* 2010, **11**(10).

[2] Robinson MD, Smyth GK: **Small-sample estimation of negative binomial dispersion, with applications to SAGE data**. *Biostatistics* 2008, **9**(2):321–332.

[3] Skaletsky H, Kuroda-Kawaguchi T, Minx P, Cordum H, Hillier L, Brown L, Repping S, Pyntikova T, Ali J, Bieri T, Chinwalla A, Delehaunty A, Delehaunty K, Du H, Fewell G, Fulton L, Fulton R, Graves T, Hou SF, Latrielle P, Leonard S, Mardis E, Maupin R, McPherson J, Miner T, Nash W, Nguyen C, Ozersky P, Pepin K, Rock S, Rohlfing T, Scott K, Schultz B, Strong C, Tin-Wollam A, Yang SP, Waterston R, Wilson R, Rozen S, Page D: **The male-specific region of the human Y chromosome is a mosic of discrete sequence classes**. *Nature* 2003, **423**:825–837.

[4] Carrel L, HF W: **X-inactivation profile reveals extensive variability in X-linked gene expression in females**. *Nature* 2005, **434**:400–404.

[5] Pickrell J, Marioni J, Pai A, Degner J, Engelhardt B, Nkadori E, Veyrieras J, Stephens M, Gilad Y, Pritchard J: **Understanding mechanisms underlying human gene expression variation with RNA sequencing**. *Nature* 2010, **464**:768–772.

[6] Hansen KD, Irizarry RA, Wu Z: **Removing technical variability in RNA-seq data using conditional quantile normalization**. *Biostatistics* 2012, **13**(2):204–16.

[7] Storey JD, Tibshirani R: **Statistical significance for genomewide studies**. *Proc Natl Acad Sci U S A* 2003, **100**(16):9440–5.
